# Accelerated Endosomal Escape of Splice-Switching Oligonucleotides Enables Efficient Hepatic Splice Correction

**DOI:** 10.1021/acsami.4c19340

**Published:** 2025-01-28

**Authors:** Silvia Weiss, Simon Decker, Christoph Kugler, Laura Bocanegra Gómez, Helene Fasching, Denise Benisch, Fatih Alioglu, Levente Ferencz, Theresa Birkfeld, Filip Ilievski, Volker Baumann, Alina Duran, Enes Dusinovic, Nadine Follrich, Sandra Milenkovic, Dajana Mihalicokova, Daniel Paunov, Karla Singeorzan, Nikolaus Zehetmayer, Dejan Zivanonvic, Ulrich Lächelt, Auke Boersma, Thomas Rülicke, Haider Sami, Manfred Ogris

**Affiliations:** † Faculty of Life Sciences, Department of Pharmaceutical Sciences, Laboratory of Macromolecular Cancer Therapeutics (MMCT), University of Vienna, Josef-Holaubek-Platz 2, 1090 Vienna, Austria; ‡ Institute of In-Vivo and In-Vitro Models, Biomodels Austria, Department of Biomedical Sciences, University of Veterinary Medicine Vienna, Veterinärplatz 1, A-1210 Vienna, Austria; § Department of Biomedical Sciences and Pathobiology, University of Veterinary Medicine Vienna and Ludwig Boltzmann Institute for Hematology and Oncology, Veterinärplatz 1, A-1210 Vienna, Austria

**Keywords:** splice correction, crosslinked polyethylenimine, intravenous, optical tomography, phosphorothioate, endosomal escape, biological barriers

## Abstract

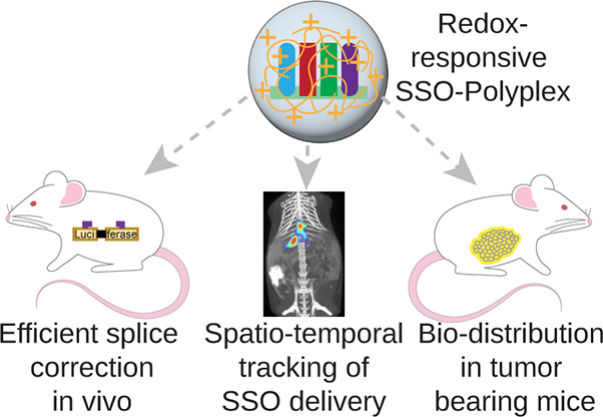

Splice-switching oligonucleotides (SSOs) can restore protein functionality in pathologies and are promising tools for manipulating the RNA-splicing machinery. Delivery vectors can considerably improve SSO functionality in vivo and allow dose reduction, thereby addressing the challenges of RNA-targeted therapeutics. Here, we report a biocompatible SSO nanocarrier, based on redox-responsive disulfide cross-linked low-molecular-weight linear polyethylenimine (cLPEI), for overcoming multiple biological barriers from subcellular compartments to en-route serum stability and finally in vivo delivery challenges. Intracellularly responsive cross-links of cLPEI significantly accelerated the endosomal escape and offered efficient SSO release to the cell’s nucleus, thereby leading to high splice correction in vitro. In vivo performance of cLPEI-SSOs was investigated in a novel transgenic mouse model for splice correction, spatiotemporal tracking of SSO delivery in wild-type mice, and biodistribution in a colorectal cancer peritoneal metastasis model. A single intravenous application of 5 mg kg^–1^ cLPEI-SSOs induced splice correction in liver, lung, kidney, and bladder, giving functional protein, which was validated by RT-PCR. Near-infrared (NIR) fluorescence imaging and X-ray computed tomography revealed improved organ retention and reduced renal excretion of SSOs. NIR microscopy demonstrated the accumulation of SSOs in angiogenic tumors within the pancreas. Successful nuclear delivery of SSOs was observed in the hepatocytes. Thus, cLPEI nanocarriers resulted in highly efficient splice correction in vivo, highlighting the critical role of the enhanced SSO bioavailability.

## 1 Introduction

**Figure 1:**
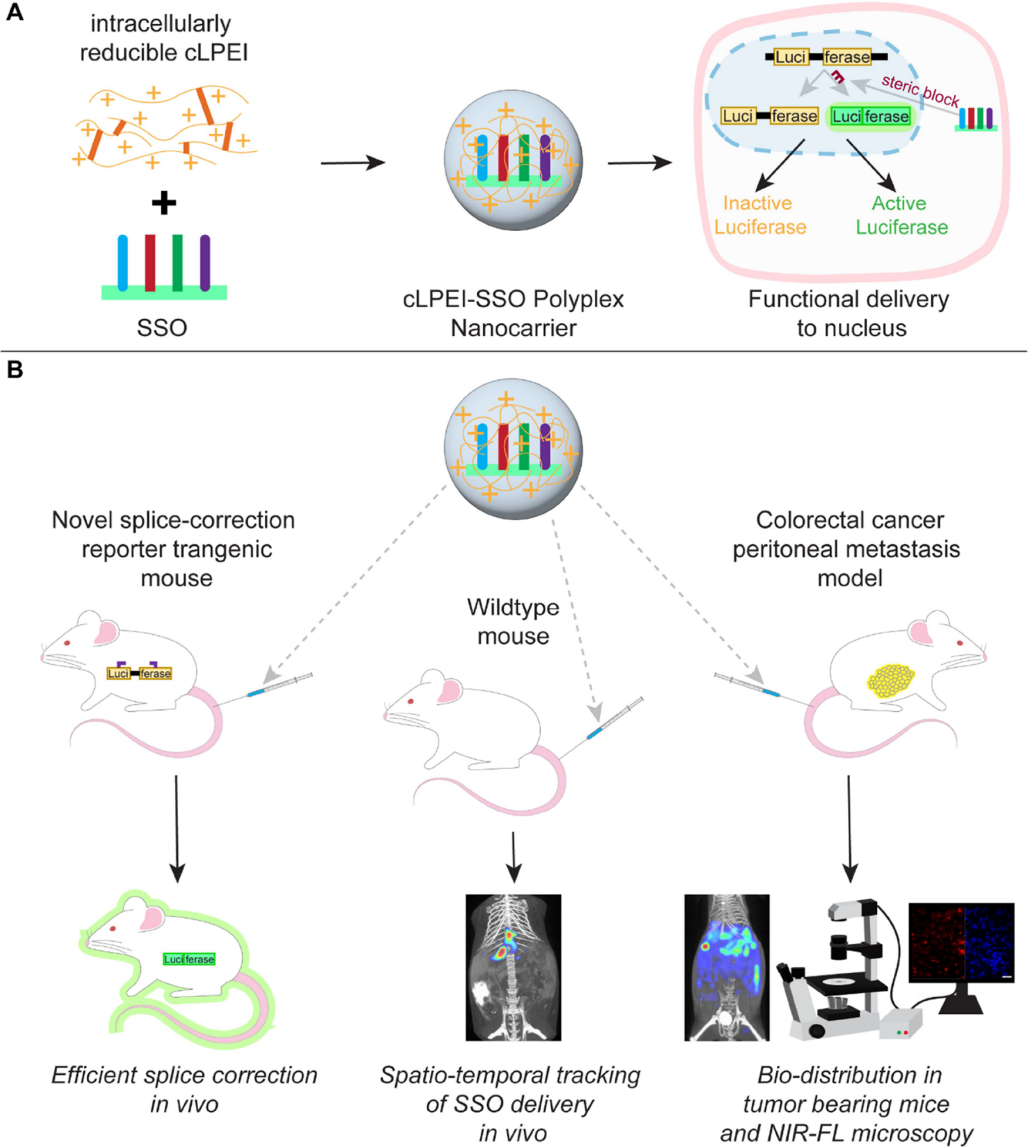
Schematic overview of intracellular-environment responsive cLPEI-polyplex-based SSO delivery in vitro and in vivo. (A) cLPEI complexes SSO into cLPEI-SSO polyplex nanocarriers possessing intracellularly degradable linkages, which are then cleaved during intracellular processing, thereby releasing functional SSO into its target organelle. cLPEI-SSO polyplex demonstrated successful splice correction in a split-luciferase reporter model in vitro where the luciferase transgene is interrupted by mutated ß-globin intron 2 producing aberrant and inactive enzyme (Luci-ferase). After successful nuclear delivery, SSOs mediated sequence-defined steric block of the ß-globin splicing site corrected the splicing from aberrant to functional (Luciferase) enzyme. (B) In vivo performance of cLPEI-SSO polyplexes was studied in three different murine models: (a) A novel transgenic split-luciferase reporter mouse, based on the above-described in vitro model, which employs bioluminescence imaging of corrected luciferase as an organ-level read-out of successful splice correction in vivo, (b) wild-type mice where in vivo delivery of 2′OMe-PS SSOs was compared between cLPEI-SSO polyplex and naked SSOs by spatiotemporal tracking using NIR fluorescence and X-ray computed tomography FLIT/CT imaging, and (c) colorectal cancer bearing mice to evaluate SSO biodistribution in tumor and organs by NIR-FLI imaging and FLIT/CT and on a cellular level using NIR-FLM.

Frameshift or nonsense mutations are among the most devastating ones causing not only monogenic diseases but also specific cases of cancer. Here, gene replacement therapy, applied ex vivo or in vivo, or, more recently, site-directed repair, e.g., in stem cells applying genome engineering, like CRISPR/Cas or related approaches can be considered.^1^ However, many of these strategies require changes on the genomic level. An alternative is the repair of primary RNA transcripts. In the case of mutations limited to a specific exon, splice-switching oligonucleotides (SSOs) can selectively block a splice site, thereby triggering removal of an aberrant exon. Here, one of the first clinical applications was to treat Duchenne muscular dystrophy (DMD). After successful preclinical and clinical evaluation, FDA approval was obtained, although being rejected by EMA due to limited efficacy.^2^ Meanwhile, SSOs have been developed for a range of monogenic diseases. In cases which require systemic application to reach affected tissues and cells, the pharmacokinetics of SSOs often exhibit low target-binding rates resulting in high and multiple doses of SSOs in the range of 30 mg kg^–1^ administered weekly.^3^ While degradation of SSOs by nucleases can be halted by applying chemically stabilized versions, e.g., with a phosphorothioate (PS) backbone or morpholino oligomers,^4,5^ improving pharmacokinetics requires formulation of SSOs. Similar as for other nucleic acids, polymer- or lipid-based strategies can be utilized.^6^ Here, biocompatibility of the carrier and its ability to enhance the in vivo bioavailability of SSOs are of utmost importance. Polycations enable the formation of small nanoparticulate structures with various nucleic acids. While the size of the nucleic acid cargo requires subtle optimization concerning structure and molecular weight (MW) of the polycation, a minimum number of positive charges per molecule is necessary to prevent premature disassembly of a polyelectrolyte complex before being internalized by the target cell.^7^ Polyethylenimine (PEI) is the polycation with the highest positive charge density and often referred to as the “gold standard” in polycation-mediated transfection. Also here, a minimum MW of PEI is necessary to enable efficient transfection.^8^ While the increased MW of the polycation increases the stability and transfection efficiency of polyelectrolyte complexes, it also raises cellular and systemic toxicities.^9^ To address this, bioresponsive polymers have been developed, where, for example, low-MW PEIs are cross-linked via reversible linker molecules, like ester linkages^10^ or disulfide bridges.^11,12^ In this study, we synthesized a biocompatible and endosome-escapable polycationic carrier with an intracellular-environment responsive function of enhanced SSO release to the nucleus, thereby achieving functional SSO delivery both in vitro and in vivo (Figure 1). Low-MW linear polyethylenimine cross-linked with intracellularly degradable disulfide linkages, termed cLPEI, was employed to successfully complex SSOs into cLPEI-SSO polyplex nanocarriers (Figure 1A). These cLPEI-SSO polyplexes were then comprehensively characterized in vitro for their biophysical properties and penetration of multiple biological barriers including endosomal escape, nuclear entry, and splice correction efficiency. As shown in Figure 1B, the optimized cLPEI-SSO polyplex nanocarrier was then employed for in vivo studies within (a) a novel transgenic split-luciferase reporter mouse model for splice correction studies, (b) wild-type mice for visualizing in vivo SSO delivery at the organ level from a spatiotemporal biodistribution point of view, and (c) a colorectal peritoneal metastasis model for biodistribution and SSO delivery at the cellular level by near-infrared fluorescence microscopy (NIR-FLM) of tissue sections of tumor-bearing mice.

## 2 Results

**Figure 2:**
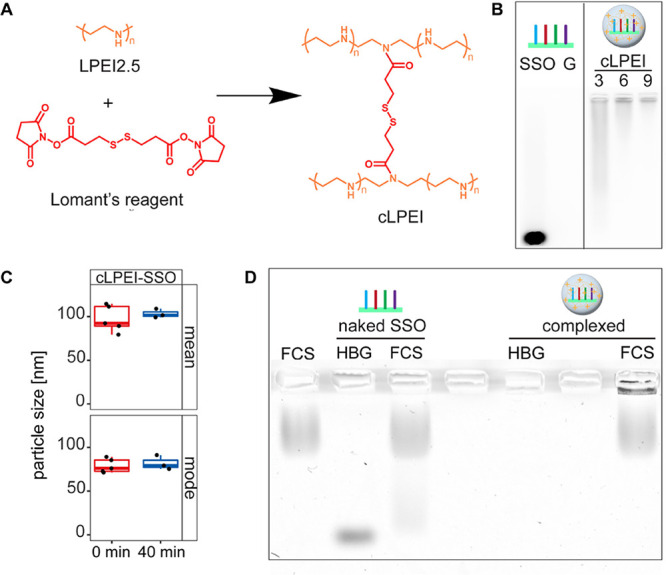
cLPEI polyplexes for efficient encapsulation, high loading, and serum stability of SSOs. Intracellular-environment responsive polyplex formation by electrostatic complexation of disulfide cross-linked cLPEI and SSOs. (A) Schematic representation of cLPEI synthesis from low-MW linear polyethylenimine (LPEI 2.5 kDa) using dithiobis(succinimidyl propionate) (Lomant’s Reagent). (B) Gel retardation assay of AF647-labeled SSO after complexation with cLPEI at indicated N/P ratios (3, 6, 9) on a 1.5% agarose gel. Naked SSO (SSO) and glycerol (G) were used as controls. (C) NTA of cLPEI-SSO polyplexes generated in HBG buffer for in vivo application at SSO concentration of 400 μg mL-1 (cLPEI-SSO-Luc, N/P 9) and measured immediately or after 40 min incubation at 25 °C; both mean and mode of the particle size distribution are shown. (D) Serum stability of N/P 9 cLPEI-SSO polyplexes. Gel retardation assay of naked SSO versus cLPEI-SSO after incubation with serum (FCS) or HBG buffer for 30 min and subsequent running on a 1.5% agarose gel. 400 ng of SSO were loaded per well; only FCS was also loaded as a control.

Electrostatic complexation of SSOs into cLPEI polyplex nanocarriers was investigated using gel retardation and light-scattering techniques. First, as depicted in Figure 2A, cLPEI was prepared by cross-linking 2.5 kDa LPEI (referred to as LPEI 2.5) by homobifunctional *N-*hydroxysuccinimide ester and characterized for cross-linking by copper assay and Ellman’s assay to estimate the cross-linking ratio at 8.3% on an average (Figure S1).

### 2.1 Cross-Linked LPEI Enables Efficient SSO Complexation, High Loading, and Stability Against Serum

Complexation of SSOs, crucial for achieving high loading, was studied at different N/P ratios from 3 to 12 (Figures 2B and S2A). N/P 3 SSO polyplexes demonstrated the presence of majorly uncomplexed SSOs, while N/P 6 SSO polyplexes gave better complexation. At N/P ratio of 9 and above (Figure S2A), complete complexation of SSOs was achieved, indicating the need of excess cLPEI for assuring 100% SSO loading. When comparing disulfide cross-linked polymer (cLPEI) with its non-cross-linked version (LPEI 2.5) for SSO complexation behavior, both polymers showed similar complexation at higher N/P ratios of 6 and 9 (Figure S3). However, at the N/P ratio 3, cLPEI seems to complex SSOs slightly more than LPEI2.5 (Figure S3). The cationic nature of polyplexes was further confirmed by zeta potential characterization, where N/P 9 cLPEI-SSO polyplexes showed a positive mean zeta potential (mean ± SD) of 21.08 ± 8.16 mV. Further, we subjected the N/P 9 cLPEI-SSO polyplex to nanoparticle tracking analysis (NTA) for estimating their size at two polyplexing concentrations: 20 μg mL^–1^ for in vitro polyplexes and 400 μg mL^–1^ for in vivo polyplexes. In the case of cLPEI-SSO polyplexes at 20 μg mL^–1^, the hydrodynamic diameter was 126.1 nm ±22.6 (mean size, mean ± sd). Toward the goal of systemic SSO delivery, cLPEI-SSO-based polyplexes were prepared at a high SSO concentration of 400 μg mL^–1^ and subjected to NTA in HBG buffer (physiologically relevant for in vivo administration) for size and concentration measurements at two dedicated time points. cLPEI-SSO polyplexes possessed an average size of 97.4 ± 15.1 nm (Figure 2C, 0 min, means ± sd), which is in a similar size range as for in vitro polyplexes. Importantly, in vivo cLPEI-SSO polyplexes appeared to be stable in HBG buffer for 40 min after complexation, as is visible from their size distribution and concentration profiles over time (Figures 2C and S2B, respectively). Values do not show a statistically significant difference between both time points using a two-sided *t* test (*p* > 0.05). Further, we tested if the presence of serum can destabilize the SSO polyplexes and lead to release/degradation of SSOs, which is relevant for systemic administration applications. This was investigated by incubating naked SSOs and cLPEI-SSO polyplexes with serum for 30 min or 4 h and then subjecting them to gel retardation assays for visualizing the possible release of SSO from polyplexes. SSOs seem to be well-protected within polyplexes for both the durations of 30 min (Figure 2D) and 4 h (Figure S2C) of exposure to serum. We also tested if cross-linking has an effect on serum stability of SSO polyplexes. As visible in Figure S4, at a N/P ratio of 9, both cLPEI-SSO and LPEI 2.5-SSO polyplexes do not exhibit SSO release on exposure to serum.

### 2.2 Disulfide Cross-Links Mediate Faster Endosomal Escape Kinetics and Higher Nuclear Delivery

**Figure 3:**
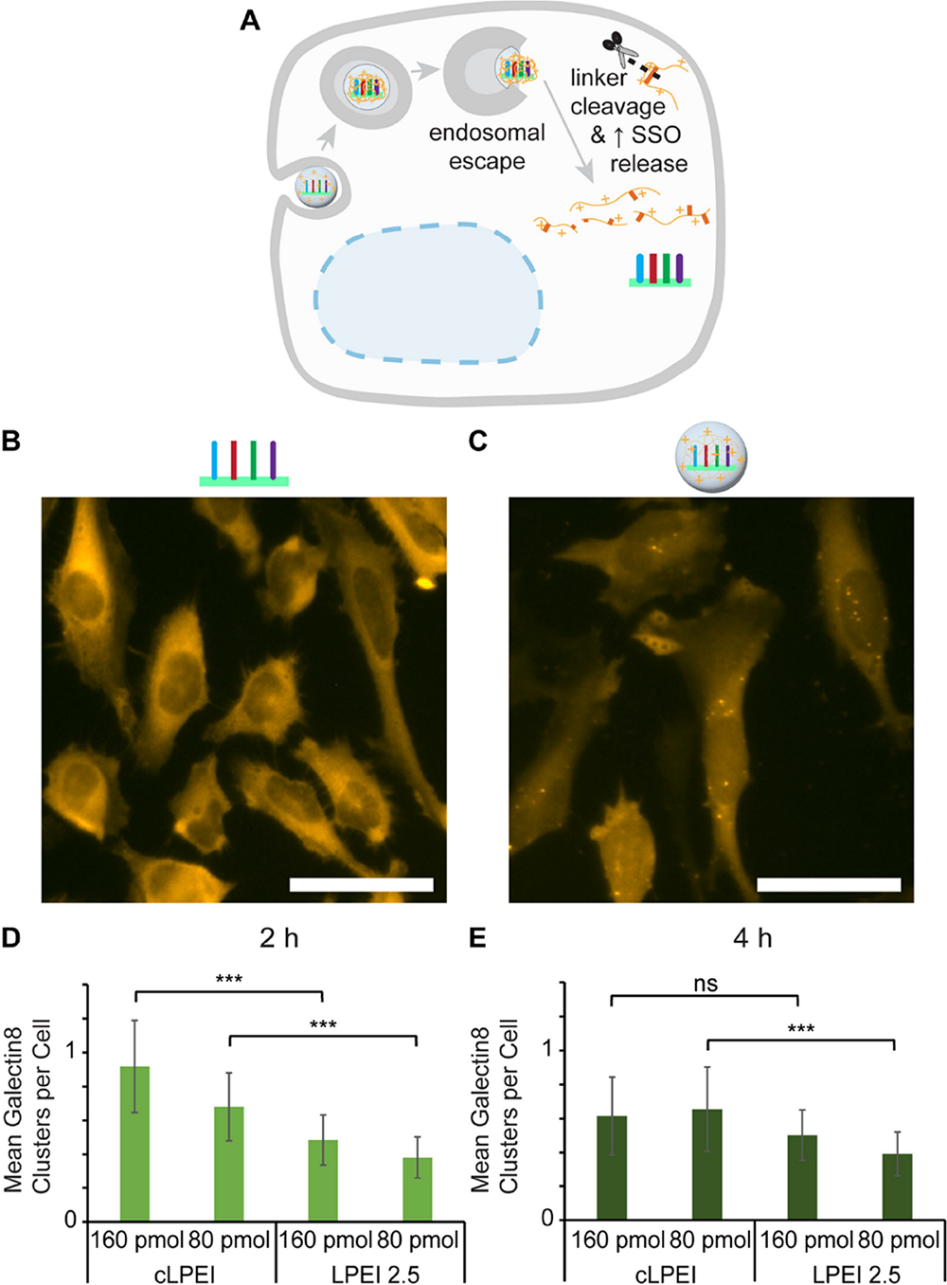
Disulfide cross-links of cLPEI enhance endosomal escape of polyplexes. (A) Schematic overview of cLPEI polyplex uptake by cells, subsequent intracellular processing, and endosomal escape in Hela mRuby-3 galectin 8 (Hela-Gal8) reporter cells, which form galectin 8 clusters, as a function of endosomal membrane disruption and imaged by FLM. FLM-based visualization of galectin 8 clustering in Hela-Gal8 cells after 4 h of incubation with 160 pmol of (B) naked SSO-NC or (C) cLPEI-SSO-NC N/P 9 polyplex. Both panels show magnified sections of images displayed in Figure S6; scale bar: 50 μm. FLM-based galectin 8 cluster quantification in Hela-Gal8 cells treated with cLPEI-SSO-NC (cLPEI) or LPEI-SSO-NC (LPEI 2.5) polyplexes at two doses of 160 or 80 pmol for treatment duration of 2 h (D) or 4 h (E). Statistics were calculated using a Welch’s *t* test (two-tailed, ***: *p* < 0.001, ns: not significant; data shown as mean ± SD.

Considering efficient endosomal escape as a critical bottleneck in nucleic acid delivery, the HeLa mRuby-3 galectin8 cell model^13,14^ was employed for studying the effect of disulfide cross-linking on endosomal escape kinetics. First, the HeLa-Gal8 model was validated for its functionality by employing chloroquine, which is a known lysosomotropic agent and works by destabilizing endosomal vesicles. When HeLa-Gal8 cells were treated with 40 μM chloroquine, characteristic Gal8 clusters were visible as fluorescent dots, indicating disruption of endosomal membranes, as can be seen in Figure S5A,B. These HeLa-Gal8 cells were then used for uptake studies with naked SSOs and cLPEI-SSO polyplexes to visualize Gal8 clustering, thereby indicating their endosomal escape behavior (Figure 3A). Cells were incubated with high doses (160 pmol) of naked SSO-negative control (SSO-NC) or cLPEI-SSO-NC polyplex for 4 h and subjected to FLM for the detection of Gal8 clusters indicating disruption of endosomal vesicles. While no clusters were present in naked SSO-NC-treated cells (Figures 3B and S6A), they could clearly be seen in cLPEI-SSO-NC polyplex-treated cells (Figures 3C and S6B). Following endosomal escape confirmation of the cLPEI-SSO polyplex, HeLa-Gal8 cells were next used to study the effect of disulfide cross-linking on endosomal escape kinetics of polyplex nanocarriers. Toward this, HeLa-Gal8 cells were incubated with different dosages (160 or 80 pmol) of either cLPEI-SSO-NC polyplexes (disulfide cross-linked polyplexes) or LPEI2.5-SSO-NC polyplexes (non-cross-linked polyplexes) and subjected to a subsequent FLM-based read-out. ImageJ was used to calculate the number of Gal8 clusters per cell, and the mean was plotted for different incubation times (Figure 3D,E). It is striking that both concentrations of cLPEI-based polyplexes depict a significantly higher number of mean Gal8 clusters per cell, when compared to their non-cross-linked LPEI 2.5-based polyplexes for the early time point of 2 h, as shown in Figure 3D. However, after 4 h, this is only true for the lower concentration of 80 pmol as presented in Figure 3E. Of note, a negative control of SSO (SSO-NC) was employed for these experiments since the used Gal8 cells did not harbor any splice-switching related transgene.

**Figure 4:**
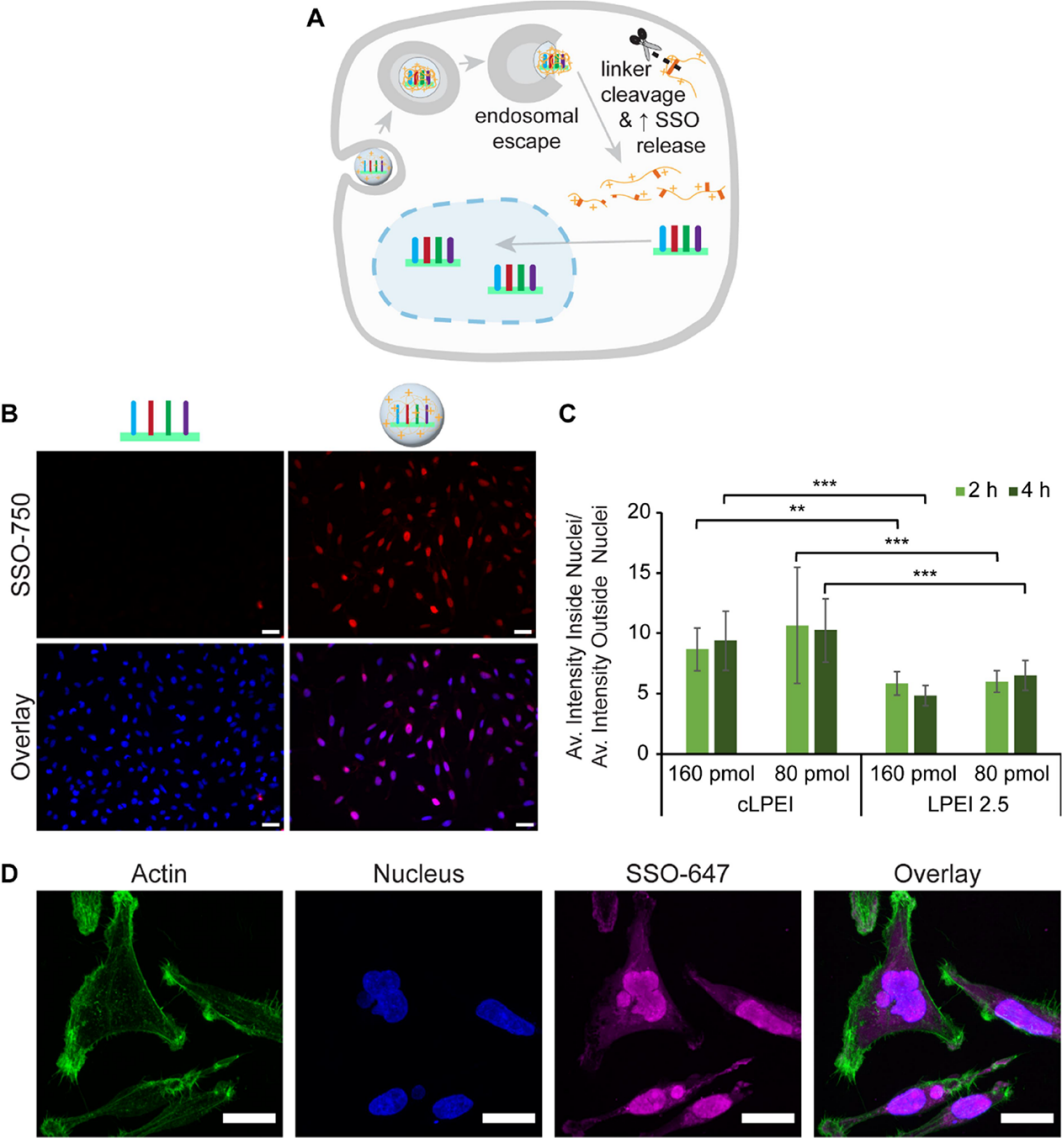
Enhanced intracellular SSO delivery to target organelle via cLPEI polyplexes. (A) Schematic overview of cLPEI polyplex uptake by cells, subsequent intracellular processing, and endosomal escape, followed by SSO release and nuclear entry, which was studied by FLM. FLM images of Hela-Gal8 cells after 4 h of incubation with 160 pmol of AF750-labeled SSOs as (B, left) naked SSO-NC-750 or (B, right) cLPEI-SSO-NC-750 N/P 9 polyplex for imaging intracellular localization of SSOs. Nuclei were stained with DAPI, and overlay with AF750-SSO is shown. Scale bar: 50 μm. (C) FLM image analysis-based AF750-SSO signal quantification in Hela-Gal8 cells treated with cLPEI-SSO-NC (cLPEI) or LPEI-SSO-NC (LPEI 2.5) polyplexes at two doses of 160 or 80 pmol for treatment duration of 2 or 4 h. AF750 intensity inside as well as outside of nuclei was measured and divided, which results in the corresponding ratio. Statistics were calculated using a Welch’s *t* test (two-tailed, **: *p* < 0.01, ***: *p* < 0.001); data shown as mean ± SD. (D) CLSM maximum intensity projection of z-stacks (63X, oil) of HeLa pLuc 705 cells after 24 h of treatment with 160 pmol of cLPEI-SSO-NC-647 N/P 9 polyplex (AF647-labeled SSO, magenta). After treatment, cells were stained with phalloidin (green, actin) and DAPI (blue, nuclei). Scale bar: 25 μm.

After demonstrating disulfide-enhanced endosomal escape of cLPEI-SSO polyplexes, we tested their ability to deliver SSOs to the target organelle, i.e., nucleus (Figure 4A). For these studies, fluorescent labels were employed to track them and visualize their intracellular localization. Alexa Fluor 750 (AF750) labeled negative control SSOs (SSO-NC-750) were used as either naked SSO-NC or cLPEI-SSO-NC polyplexes. After an incubation time of 4 h, cells were analyzed using FLM; the same exposure time was used for different treatment groups. As visible in Figure 4B, while no SSO-derived signal could be detected for cells incubated with naked SSO-NC-750, cLPEI-SSO-NC-750 polyplex-treated cells showed an intense AF750-SSO signal in almost all nuclei, as proven by the overlay with the nuclear stain 4′,6-diamidino-2-phenylindole (DAPI). Since it is known from literature that naked SSO should also be able to enter mammalian cells on their own, the exposure time was increased from 100 ms to a maximum of 5 s, to be able to visualize any minute amounts of SSOs present in the nucleus of naked SSO-treated cells (Figure S7). As expected, a nuclear signal could then be detected, albeit a weak one, while a possible autofluorescence signal could be excluded due to the lack of any similar signal in buffer-treated cells. Next, the effect of disulfide cross-links on nuclear delivery of SSOs was studied by comparing cLPEI-SSO-NC polyplexes (disulfide cross-linked polyplexes) with LPEI2.5-SSO-NC polyplexes (non-cross-linked polyplexes) for AF750-SSO signal quantification in nucleus from FLM images. ImageJ program was used to calculate the percentage of AF750-positive nuclei (Figure S8A) as well as to measure the AF750 signal intensity inside nuclei (Figure S8B) and outside of nuclei (Figure S8C). Ratio of AF750 signal intensity inside the nuclei to that outside the nuclei (Figure 4C) was calculated because of the apparent differences in AF750 signal localization within cells treated with cLPEI-SSO or LPEI2.5-SSO polyplexes (Figure S9). While with both formulations the percentage of AF750-positive cells is similar, in the case of cLPEI polyplexes, AF750 signal appears to be mainly in the nucleus. However, for LPEI2.5 polyplexes, with the same treatment duration as that of cLPEI polyplexes, the signal was also within the extra-nuclear space (Figure S9). Therefore, the ratio of both averages was plotted for cells incubated with different dosages (160 or 80 pmol) of either cLPEI-SSO-NC-750 or LPEI2.5-SSO-NC-750. At both time points of 2 and 4 h, cLPEI-based polyplexes showed significantly higher ratios compared to LPEI2.5-based polyplexes (Figure 4C). Apparently, as the total cellular uptake of cLPEI-SSO-NC-750 and LPEI2.5-SSO-NC-750 seems similar, there are significantly more SSOs reaching the nucleus when applying cLPEI-SSO-NC-750 polyplexes, which is in line with its improved endosomal release.

To additionally validate nuclear delivery, confocal laser scanning microscopy (CLSM) was employed for later time points of cellular uptake. Therefore, cLPEI-SSO-NC polyplexes were labeled with the fluorophore Alexa Fluor 647 (AF647, suitable for CLSM) and added to HeLa pLuc 705 cells. After 24 h of incubation, cells were subjected to CLSM. The polyplexes not only entered the cells but also mediated efficient nuclear delivery of SSO as visible in the maximum intensity projections (Figures 4D and S10A) and middle-most section of cells (Figure S10B).

### 2.3 Cross-Linked LPEI Enable Functional Oligo Delivery to Facilitate Enhanced Splice Correction

**Figure 5:**
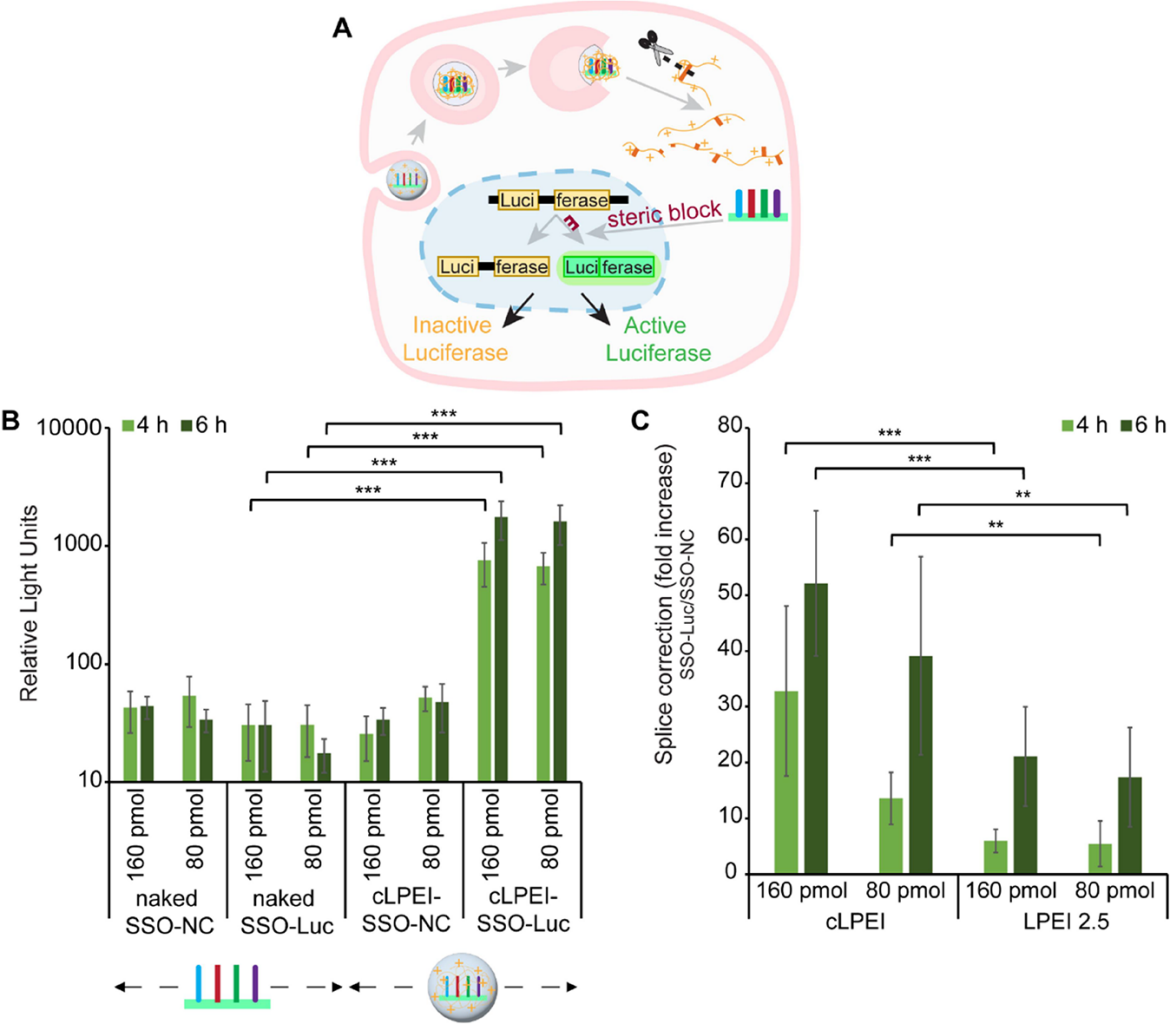
CLPEI mediated SSO complexation enhances functional oligo delivery in vitro. (A) Schematic overview of HeLa pLuc 705 reporter cells (which express split-luciferase, i.e., luciferase interrupted by mutated ß-globin intron 2, producing aberrant and inactive enzyme) showing intracellular processing of cLPEI polyplexes to release SSOs to the nucleus for steric-blocking of the ß-globin splicing site, thereby correcting the splicing from aberrant (Luci-ferase) to functional enzyme (Luciferase). Luciferase assay-based luminescence is thus used as a read-out of SSO-mediated splice correction. (B) Luminescence measurements from HeLa pLuc 705 cells after treating with 160 or 80 pmol of naked SSOs or cLPEI-SSO polyplexes. (C) Luminescence measurements from HeLa pLuc 705 cells after treating with 160 or 80 pmol of cLPEI-SSO or LPEI2.5-SSO polyplexes. Both luciferase-correcting SSOs (SSO-Luc) or negative control SSOs (SSO-NC) were used. Firefly luciferase assay was performed after 4 or 6 h and either RLUs were plotted directly (as shown in B) or splice correction was calculated as fold increase by dividing the luminescence from Luc-SSO treatments by that from NC-SSO treatments (as shown in C). Statistics were calculated using a Welch’s *t* test (two-tailed, **: *p* < 0.01, ***: *p* < 0.001); data shown as mean ± SD.

To reach a high splice correction efficiency, both effective intracellular delivery and splice correction function of SSOs are of importance. One possible read-out to measure the splice-switching capacity is via bioluminescence because of its sensitivity. Toward this, we utilized the HeLa pLuc 705 cell-based split-luciferase model,^15^ which employs firefly luciferase assay-based luminescence as a read-out of functional SSO delivery and splice correction, as shown in Figure 5A. Before using the HeLa pLuc 705 model for our studies, we first investigated the transgenic HeLa cells for the assay sensitivity and background splice correction levels. These cells showed slightly higher luminescence when compared to the same cell number of wild-type HeLa cells (Figure S11), indicating the presence of some basal levels of splice correction. However, when the relative light units (RLU) of Hela pLuc 705 were normalized to the protein amount of the corresponding cell number, the luminescence read-out was similar for all cell numbers. Therefore, this cell line could be used as a sensitive reporter assay for splice correction, as has been routinely used in other studies. To validate the sequence specificity of SSOs for steric-block-mediated splice correction (Figure 5A), luciferase-correcting SSOs (SSO-Luc) or sequence-scrambled control SSOs (SSO-NC) were used for all splice correction experiments. First, the splice correction efficiency of cLPEI polyplexes was compared with naked SSOs. As shown in Figure 5B, both the concentrations and incubation times of cLPEI-SSO-Luc lead to a statistically significant increase in the luminescence signal compared to both cLPEI-SSO-NC polyplexes or naked SSOs of Luc and NC sequences.

**Figure 6:**
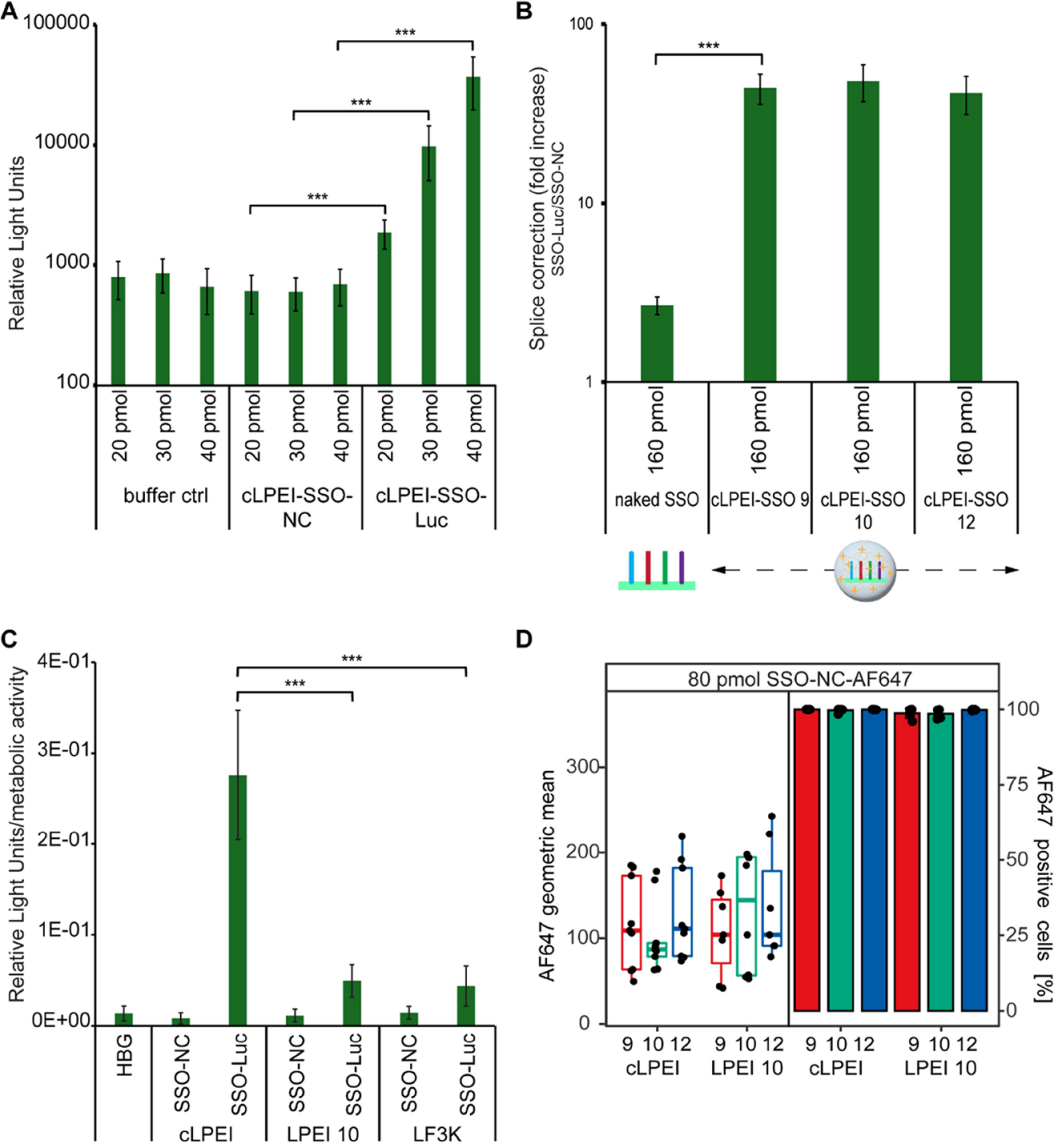
CLPEI-SSO polyplexes for dose-modulated splice correction and cell uptake efficiency. (A) Luminescence measurements from HeLa pLuc 705 cells 24 h after treatment with cLPEI-SSO polyplexes loaded with different doses of SSOs (20, 30, and 40 pmol). As a control, buffer was used in amounts corresponding to the different treatments; *n* = 9. (B) Luminescence measurements from HeLa pLuc 705 cells 24 h after treatment with naked SSO or cLPEI-SSO polyplexes of different N/P ratios (N/P 9, 10, and 12). Both luciferase-correcting SSOs (SSO-Luc) and negative control SSOs (SSO-NC) were used. Firefly assay was performed, and splice correction was calculated as fold increase by dividing the luminescence from Luc-SSO treatments by that from NC-SSO treatments, *n* = 9. (C) Comparison of cLPEI polyplexes with LPEI 10 and lipofectamine 3000 based formulations for transfection ability and biocompatibility profile. Luminescence measurements normalized by cell viability (based on metabolic activity) from HeLa pLuc 705 cells 24 h after treatment with different SSO polyplexes, as indicated, are shown. The dose of SSO, either NC or Luc, was kept constant at 160 pmol, while different complexing agents were used: cLPEI, LPEI 10, and lipofectamine 3000 (LF3K). As a control, buffer was used in amounts corresponding to the different treatments; *n* = 12. (D) Cellular association of SSO-NC-647 (80 pmol) polyplexes with HeLa pLuc 705 cells 24 h after treatment. Both cLPEI and LPEI 10 were used at different N/P ratios (N/P 9, 10, and 12), and geometric mean of AF647 (left) as well as percentage of AF647 positive cells (right) are shown, *n* = 5. Statistics were calculated using a Welch’s *t* test (two-tailed, **: *p* < 0.01, ***: *p* < 0.001); data shown as mean ± SD.

To investigate if disulfide-enhanced endosomal escape and nuclear delivery also translates into increased splice correction functionality, HeLa pLuc 705 cells were treated with N/P 9 cLPEI-SSO (disulfide cross-linked polyplexes) or LPEI2.5-SSO polyplexes (non-cross-linked polyplexes) at two concentrations (80 or 160 pmol SSOs) and for two treatment durations of 4 and 6 h. Shorter incubation times were selected, similar to those of endosomal escape and nuclear entry experiments, to correlate the results from different studies. Interestingly, the splice correction efficiency of cLPEI polyplexes was significantly higher than that of LPEI2.5 polyplexes at all tested SSO doses and incubation times (Figure 5C). To test the enhanced efficiency of cLPEI polyplexes, cells were treated with low doses of 20, 30, or 40 pmol of cLPEI-SSO-NC or cLPEI-SSO-Luc for 24 h. As a control, HBG buffer was added in corresponding amounts to the cells. cLPEI-SSO-NC-treated cells did not show any increase in luminescence and had values similar to buffer control, irrespective of the dosage of the SSO treatment (Figure 6A). On the other hand, cLPEI-SSO-Luc-treated cells showed significantly increased luminescence and thereby splice correction already at the lowest tested dose of 20 pmol and thus demonstrated a dose-modulated splice correction response. Higher N/P ratios were also tested to see whether excess cLPEI can further increase the splice correction levels. cLPEI-based SSO-NC or SSO-Luc polyplexes prepared at different N/P ratios of 9, 10, and 12 were added to HeLa pLuc 705 cells, and splice correction was measured by firefly assay-based luminescence measurements. Splice correction values for cells treated with cLPEI-SSO-Luc were similar among the different N/P ratios of 9, 10, and 12 (Figure 6B). Notably, cLPEI-based SSO polyplexes showed a splice correction that was significantly higher than naked SSOs. At an SSO dose of 160 pmol (0.8 μM), 50–100-fold higher splice correction was achieved by cLPEI-SSO polyplexes in comparison to naked SSOs. Considering similar splice correction values at different N/P ratios and better performance of cLPEI when compared with LPEI 2.5 (Figures 3–5), it was decided to continue with mainly cLPEI-based polyplexes for all further experiments. To compare the in vitro performance of cLPEI with other widely used cationic formulations, cLPEI-SSO polyplexes were directly compared with LPEI 10 and lipofectamine 3000 (LF3K) based SSO formulations for transfection ability and biocompatibility profile (Figures 6C and S12). cLPEI-SSO-Luc-treated HeLa pLuc 705 cells show significantly higher firefly luciferase luminescence values, both when normalized by cell viability of the respective treatment (Figure 6C) and when analyzed as a fold increase (Figure S12A), in comparison to LPEI 10-SSO-Luc and LF3K-SSO-Luc treated cells. When comparing the cell association and uptake efficiency, flow cytometry data at 40 pmol (0.2 μM) and 80 pmol (0.4 μM) SSOs showed efficient cell association for both cLPEI-SSO and LPEI 10-SSO polyplexes (Figures 6D and S13). Cell association data are plotted as geometric mean fluorescence intensities and percentage positive cells for AF647-SSO. Here, it is clearly visible that there were no apparent differences in the cell association of SSO polyplexes across the two polymers and also no major differences for different N/P ratios within the same polymer type (Figures 6D and S13A).

### 2.4 Split-Luciferase Transgenic Mouse Model for Splice Correction Efficiency Investigation In Vivo

To study the in vivo splice correction efficiency of SSOs, we developed a transgenic reporter mouse model expressing a split-luciferase transgene under the constitutively active Rosa26 promoter, which should show functional luciferase activity wherever SSOs are functionally delivered. Of the three transgenic lines obtained, one had a Y-chromosomal integration of the transgene and was therefore abandoned. The two remaining lines were comprehensively characterized for split-luciferase transgene presence in genomic DNA (Figure S14A1), basal bioluminescence (for background splice correction, if any) (Figure S14A2), and organ level split-luciferase mRNA expression (Figure S14A3). One line was selected for further investigation and designated B6N-*Tyrc*-Tg(ROSA-Luc705)740Biat.

The mutant line was kept hemizygous for the transgene, and PCR-based genotyping was performed from genomic DNA across different generations to distinguish between transgene-positive and wild-type offspring (Figure S14B1). Ex vivo organ bioluminescence imaging (BLI) of mice carrying the split-luciferase transgene showed basal levels of bioluminescence signals which were slightly higher than organs of wild-type mice but not statistically different (Figure S14B2). After imaging, organs of split-luciferase transgenic mice were further characterized for the presence of split-luciferase mRNA by reverse transcription PCR (RT-PCR) (Figure S14B3). The established RT-PCR workflow demonstrated that split-luciferase mRNA was expressed in all organs of interest, namely, heart, lungs, liver, spleen, kidneys, and bladder of all transgene-positive mice across different generations, thereby confirming the suitability of the transgenic model for further splice correction studies. After RNA isolation and storage (RNA quality control by a ratio of 28*S*/18S rRNA), the RT-PCR workflow was optimized for the specific detection of splice correction on an mRNA level of organs of transgenic mice. Two different primer sets were employed: one for the detection of split-luciferase mRNA (luciferase sensitive primer LSP) and another for the detection of splicing (splice sensitive primer SSP) in the organs of split-luciferase transgenic mice. Annealing temperatures were optimized for these primer pairs as shown in Figure S15.

### 2.5 In Vivo Splice Correction Mediated by cLPEI Polyplexes in Split-Luciferase Transgenic Mice

**Figure 7:**
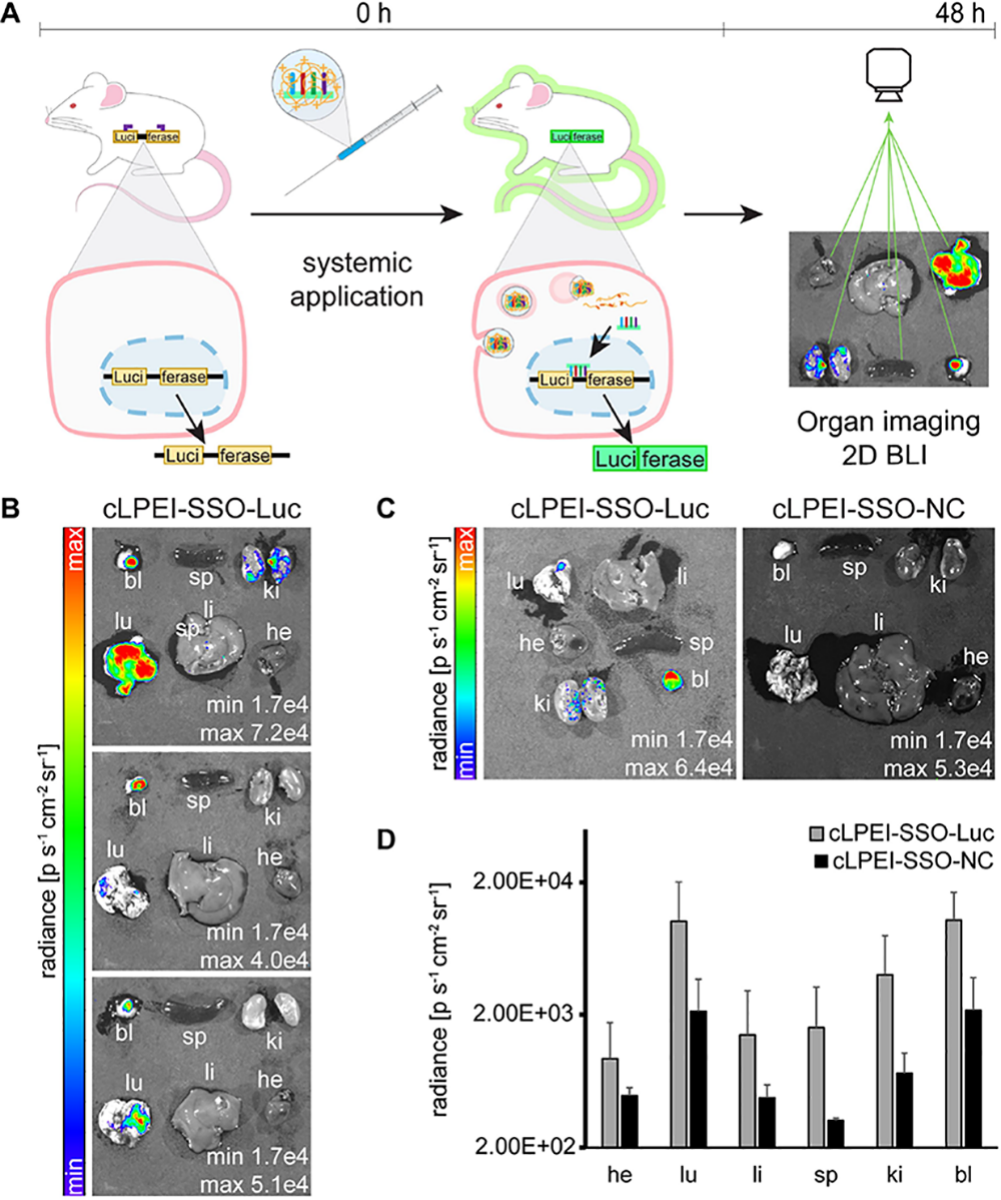
In vivo splice correction by cLPEI-SSO polyplexes produces functional luciferase protein. (A) Schematic overview of a novel transgenic reporter mouse line ubiquitously (under constitutive Rosa26 promoter) expressing split-luciferase, i.e., luciferase interrupted by mutated ß-globin intron 2, similar to HeLa pLuc 705 in vitro model, thereby producing an aberrant and inactive luciferase enzyme (Luci-ferase) and its corresponding primary RNA transcript. Successful in vivo delivery of SSO-Luc (luciferase-correcting SSO) to the nucleus corrects this aberrant split-luciferase mRNA transcript, which results in the production of functional luciferase enzyme (Luciferase) and can be visualized by 2D BLI of organs. This split-luciferase transgenic mouse was used to indicate the in vivo splice correction performance of cLPEI-SSO polyplexes after 48 h of systemic administration. (B) 2D BLI images of organs from three split-luciferase transgenic mice 48 h after systemic application of N/P 9 cLPEI-SSO-Luc polyplexes at an SSO dosage of 5 mg kg^–1^. (C) Representative 2D BLI images and (D) BLI signal quantification of organs from split-luciferase transgenic mice treated with N/P 9 cLPEI polyplexes either loaded with luciferase-correcting SSO (cLPEI-SSO-Luc) or negative control SSO (cLPEI-SSO-NC), treatment dosage of 5 mg kg^–1^ SSO, and treatment duration of 48 h; *n* = 2–3. he: heart, lu: lung, li: liver, sp: spleen, ki: kidney, and bl: bladder; data shown as mean ± SD.

Toward the goal of systemic SSO delivery, N/P 9 cLPEI-based polyplex treatment was prepared at a dose of 5.0 mg kg^–1^ SSO (which corresponds to a cLPEI dose of 5.9 mg kg^–1^). 48 h after intravenous administration of cLPEI-SSO-Luc into split-luciferase transgenic animals, mice were subjected to in vivo whole body as well as ex vivo organ 2D BLI (Figure 7A). In this specific case, BLI values above a certain threshold (radiance of 1.7E+04 ps^–1^ cm^–2^ sr^–1^) correspond to the functional luciferase enzyme as a result of successful intracellular delivery of SSOs to the nucleus in vivo. However, within whole body BLI, it was not possible to overcome the mentioned threshold after cLPEI-SSO-Luc treatment and, as a result, measured values were not significantly different from untreated or control (cLPEI-SSO-NC)-treated animals. Nevertheless, once the mice were sacrificed and organs were imaged by 2D BLI, functional luciferase could be detected in all animals receiving N/P 9 cLPEI-SSO-Luc polyplexes (Figure 7B). cLPEI-polyplex-mediated in vivo splice correction was further validated by taking split-luciferase positive transgenic mice and treating them with negative control SSO-based cLPEI polyplexes for comparison of BLI signals with cLPEI-SSO-Luc polyplex-treated mice. As visible in Figure 7C, cLPEI-SSO-NC-treated mouse organs show no BLI signal. When these 2D BLI signals were quantified, it was clearly visible that the radiance values for all organs for cLPEI-SSO-Luc polyplex-treated mice were higher than those of cLPEI-SSO-NC-treated mice (Figure 7D). Of all mice treated with cLPEI polyplexes, no acute toxicity was observed.

**Figure 8:**
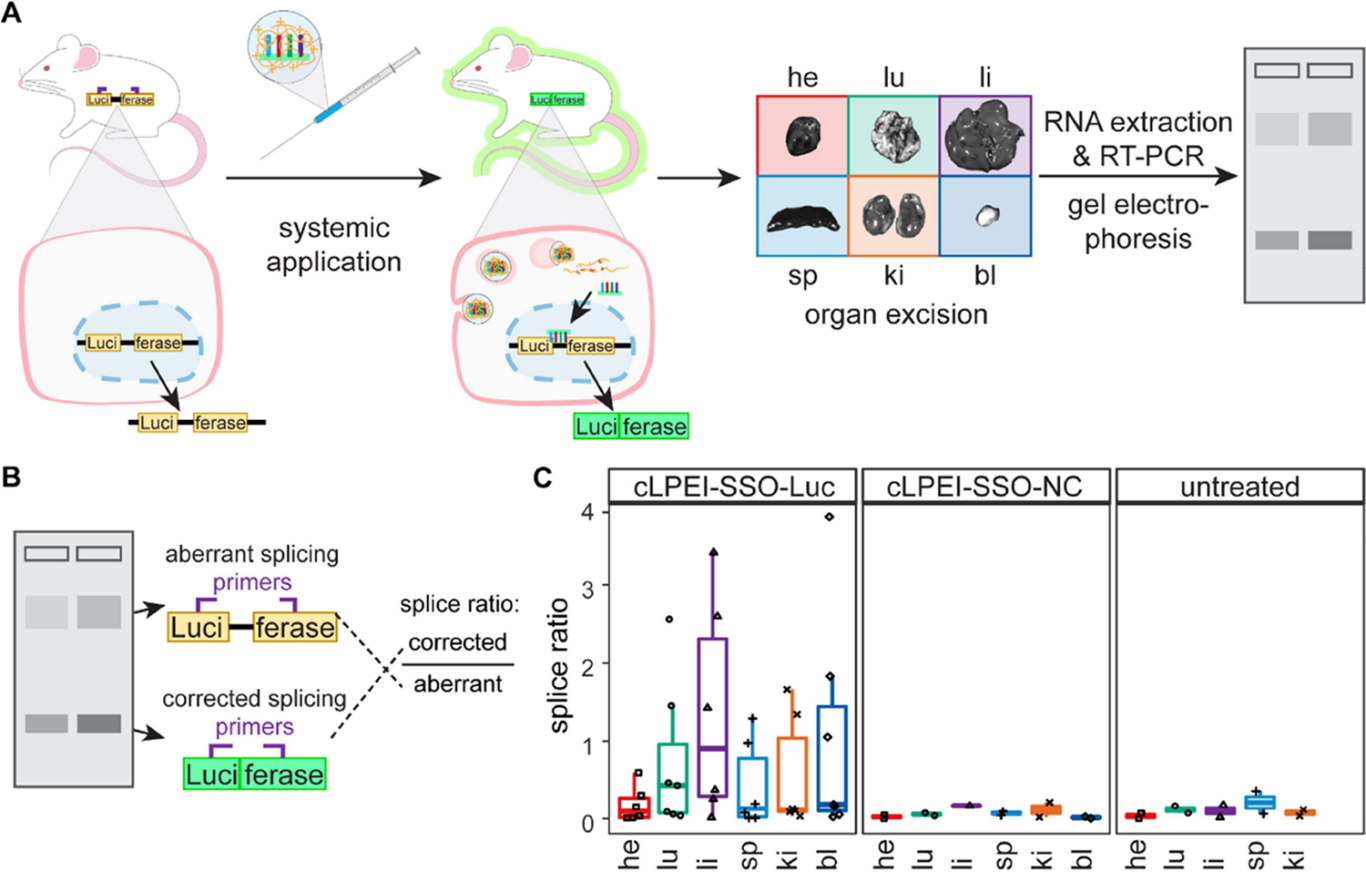
Validation of cLPEI-SSO polyplex-mediated in vivo splice correction by RT-PCR. (A) Schematic overview of split-luciferase transgenic mice ubiquitously expressing primary mRNA transcript of aberrant luciferase (Luci-ferase), which gets splice-corrected to uninterrupted luciferase primary mRNA transcript (Luciferase) in organs where luciferase-correcting SSOs are successfully delivered to the nucleus by cLPEI polyplexes. RNA of split-luciferase mice organs was subjected to RT-PCR with an intron-spanning primer pair to analyze the aberrant and corrected luciferase transcripts, with different amplicon lengths, by gel electrophoresis. (B) Schematic of agarose gel electrophoresis read-out showing the two amplicons that are separated because of different lengths, corresponding to aberrant luciferase and corrected luciferase. Gel image analysis was done to quantify band intensities and calculate the splice ratio by dividing the corrected amplicon band intensity by the aberrant amplicon band intensity. (C) Splice ratios calculated from gel electrophoresis and image analysis of organs from Figure 7D, i.e., organs from split-luciferase transgenic mice treated with N/P 9 cLPEI polyplexes either loaded with luciferase-correcting SSO (cLPEI-SSO-Luc) or negative control SSO (cLPEI-SSO-NC) or untreated. Treatment dosage of 5 mg kg^–1^ SSO and treatment duration 48 h. he: heart, lu: lung, li: liver, sp: spleen, ki: kidney, and bl: bladder; data shown as mean ± SD.

To obtain a quantitative measure for splice correction on the mRNA level and validate the BLI-based read-out of cLPEI-SSO polyplex for in vivo transfections, RNA was isolated from the tissues of untreated or either cLPEI-SSO-Luc or cLPEI-SSO-NC treated split-luciferase transgenic mice (Figure 8A). The organs in question were heart, lung, liver, spleen, kidney, and bladder. Probes were then subjected to RT-PCR with intron-spanning primer pair (Figure S15) to analyze the aberrant and corrected luciferase transcripts, with different amplicon lengths, by gel electrophoresis, where image analysis was used to estimate the ratios of correct to aberrant splicing and in vivo splice correction efficiency presented either as the splice ratio or the percentage splice correction (Figure S16). The exposure time for gel image acquisition was optimized to avoid pixel saturation for proper detection of splice-corrected bands (data not shown). The image analysis software then calculated the intensity of aberrant as well as corrected luciferase bands and their ratio (corrected:aberrant) as presented in Figure 8B. Organ samples with very low signal intensities for any luciferase band would have led to very high but incorrect splice ratio and were therefore excluded from analysis. As visible in Figure 8C, each analyzed organ of animals treated with cLPEI-SSO-NC polyplex, i.e., NC-SSO polyplex, has similar splice ratios as the corresponding organ of untreated mice. Interestingly, cLPEI-SSO-Luc polyplex-treated animals depicted high splice ratios for almost all investigated organs, with liver, lungs, and bladder showing the highest increase in splice ratio compared to untreated split-luciferase positive transgenic animals.

### 2.6 CLPEI Complexation Alters SSO Biodistribution In Vivo: Spatiotemporal Tracking of Delivery

**Figure 9:**
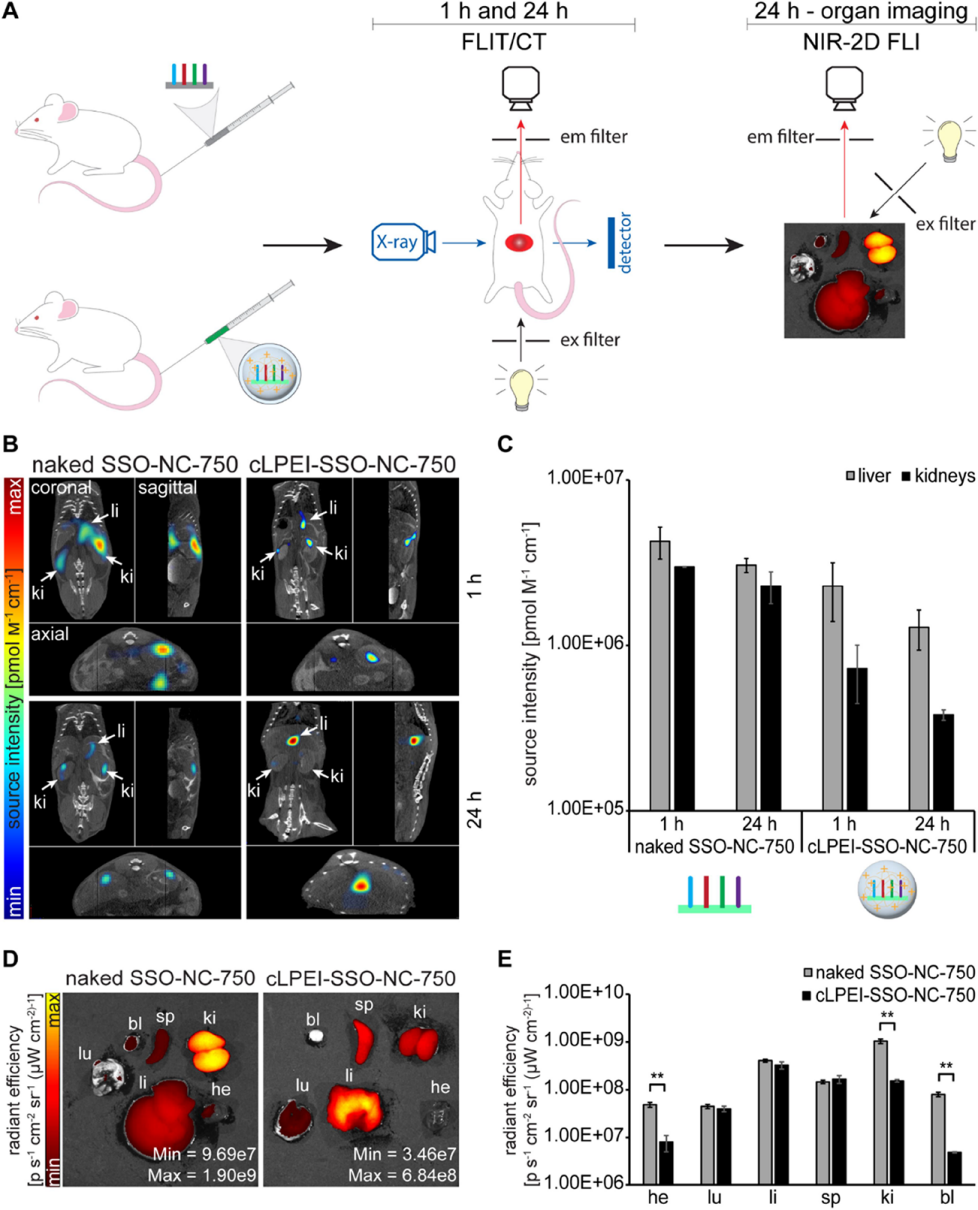
Spatiotemporal tracking of biodistribution differences between naked 2′OMe-PS SSOs and cLPEI-complexed 2′OMe-PS SSOs. (A) Schematic showing wild-type animals after systemic administration of NIR dye AF750 labeled naked SSO (naked SSO-NC-750) or cLPEI-SSO polyplex (cLPEI-SSO-NC-750) and biodistribution events followed noninvasively after 1 and 24 h using FLIT/CT (presented in B and C), followed by ex vivo organ imaging using 2D epifluorescence imaging (presented in D and E). AF750-SSO-NC (negative control SSO labeled with AF750) was employed for these studies with *n* = 3 per treatment. (B) Representative FLIT/CT images after systemic administration of naked SSO-AF750 or cPLEI-SSO-750 polyplexes. AF750 signals in liver (li) and kidneys (ki) are indicated by white arrows. (C) AF750 signal quantification from FLIT/CT, presented in part B. (D) Representative 2D FLI organ images 24 h after systemic administration. (E) Fluorescence signal quantification from 2D FLI of organs presented in D. Statistics- two-sided *t* test (**: *p* < 0.01). he: heart, lu: lung, li: liver, sp: spleen, ki: kidney, bl: bladder; data shown as mean ± SD.

Wild-type B6-albino mice were intravenously injected with NIR dye AF750 labeled 2′OMe-PS SSOs either as naked SSOs (SSO-NC-750) or cLPEI-SSO polyplexes (cLPEI-SSO-NC-750) and subjected to noninvasive spatiotemporal tracking via fluorescence imaging tomography/X-ray absorption computed tomography (FLIT/CT) (Figure 9A) for investigating their biodistribution differences in vivo. Here, CT-contrast agents aided in organ delineation for better visualization.^16^ In vivo imaging was performed twice, 1 and 24 h after SSO-NC-750 administration. Afterward, the mice were euthanized, and NIR-based 2D FLI organ imaging was conducted. Complexation seemed to alter the biodistribution pattern of SSO-NC-750 when compared to naked SSO-NC-750 (Figure 9B). While naked SSO-NC-750 were clearly present in kidneys and liver, kidney signals were seemingly reduced in cLPEI-SSO-NC-750-treated mice after 1 h of administration. After 24 h, the ratio between kidney and liver signals was consistent for naked SSO-NC-750; however, with cLPEI-SSO-NC-750, it seemed shifted with an even lower kidney signal and an apparent accumulation in the liver. This trend is also supported by the semiquantitative analysis shown in Figure 9C. FLIT/CT results were further validated by the 2D FLI of explanted organs (Figure 9D). For heart, kidneys, and bladder, radiant efficiency signals were significantly reduced for cLPEI-SSO-NC-750 polyplexes when compared to naked SSO-NC-750 (Figure 9E), thus confirming differences in the biodistribution pattern with a faster renal excretion of the uncomplexed version of SSOs.

### 2.7 CLPEI Polyplexes Facilitate SSO Accumulation in Pancreatic Tumor Nodules and Successful Nuclear Delivery to Hepatocytes

**Figure 10:**
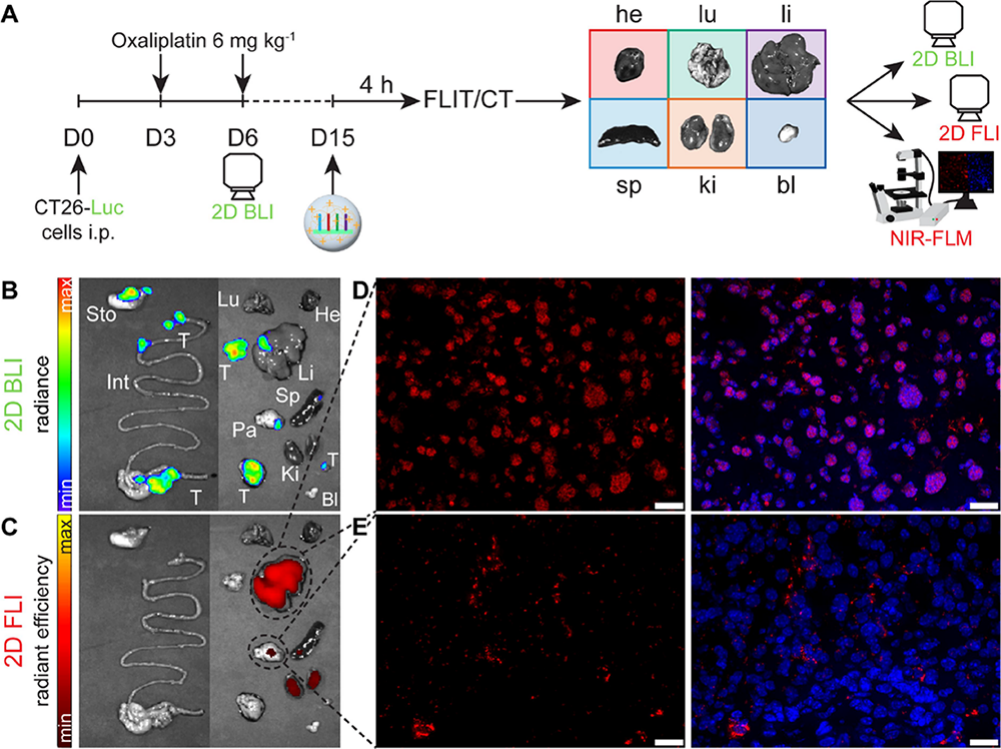
Biodistribution of SSO in colorectal metastasis tumor-bearing mice after systemic administration of cLPEI-SSO polyplex nanocarrier. (A) Schematic showing tumor implantation and treatment schedule including CT26-Luc tumor imaging by firefly luciferase bioluminescence imaging (2D BLI). On day 15, CT26-Luc tumor-bearing mice received i.v. injection of cLPEI-SSO polyplexes at SSO-NC dose of 5 mg kg^–1^ (part of total dose contained AF750 labeled SSOs, SSO-NC-750, for NIR fluorescence tracking), and SSO biodistribution followed noninvasively after 4 h using FLIT/CT. Ex vivo organ imaging was performed to visualize tumor nodules via 2D BLI and SSO presence by 2D FLI for organ-level investigation. The same set of organs is shown for both tumor presence using 2D BLI (B) and SSO accumulation using 2D FLI (C). Selected organs were then subjected to NIR-FLM analysis (presented in parts D and E) for visualizing nuclear delivery of SSO. Representative NIR images of (D) liver and (E) pancreatic tumor nodules are shown. The left panels show AF750-labeled SSO signal (red), while the right panels show the overlay with DAPI-stained nuclei (blue). Scale bar: 20 μm. he: heart, lu: lung, li: liver, sp: spleen, ki: kidney, bl: bladder, sto: stomach, t: tumor, and int: intestines.

Biodistribution of SSO was additionally evaluated in mice bearing intraperitoneal colorectal tumors. In brief, CT26-Luc cells were intraperitoneally injected into BALB/c mice (Figures 10A and S17A). Oxaliplatin was administered on days 3 and 6 after implantation; homogeneous tumor growth was confirmed in all 7 animals (M1 to M7) by BLI after subcutaneous luciferin administration on day 6 (Figure S17B). On day 15, CT26-Luc tumor-bearing animals were divided into different treatment groups (Figure S18B). M1 was untreated control animal. Naked SSO-NC-750 (M2, M3, and M4) or cLPEI-SSO-NC-750 (M5, M6, and M7) were systemically administered and FLIT/CT performed 4 h thereafter. Subsequently, mice were euthanized, and 2D organ BLI as well as 2D NIR-FLI was performed. Tissue sections of selected organs were later subjected to histological analysis by hematoxylin and eosin staining along with NIR-FLM of the AF750-SSO signal (Figure S18A).

FLIT/CT results (Figure S19A) showed the presence of naked SSO-NC-750 in kidneys and liver, while cLPEI-SSO-NC-750 was mainly concentrated in the liver area (Figure S19B). This was supported by the semiquantitative analysis (Figure S19C) and consistent with findings in wild-type animals, with the exception that in the 4 h experiment, the kidney signal was even lower for cLPEI-SSO-NC-750-treated mice. When comparing 2D BLI (Figure 10B) to 2D FLI (Figure 10C), it is visible that SSOs did not accumulate in individual tumor nodules located within the lower abdomen, but within the pancreas, where also tumor lesions can be seen via 2D BLI, the fluorescence signal from SSOs was obvious. Similar results were observed in other mice of the respective treatment group (Figure S20).

For histological evaluation and NIR-FLM, liver and lung were selected since those organs showed high values regarding splice ratio in transgenic animals, while the pancreas and individual tumor nodules were chosen from biodistribution in tumor aspects (Figure S22). Analysis by NIR-FLM revealed nuclear accumulation in hepatocytes of all cLPEI-SSO-NC-750-treated mice (Figures 10D and S23), which could not be observed for animals receiving naked SSO-NC-750 (Figure S23). Analysis of lung, nontumorigenic areas of the pancreas, and individual tumor nodules showed no nuclear accumulation of SSO-NC-750 (Figures S24–S26, respectively). Nevertheless, significant tissue accumulation but extra-nuclear signals could be detected within pancreatic tumor nodules of cLPEI-SSO-NC-750-treated mice (Figures 10E, S27 and S28). Finally, the overall morphology of lung, liver, and pancreas was evaluated by hematoxylin and eosin, and whole-organ scans presented no difference to the organs of untreated animals, thereby confirming that cLPEI did not induce any morphologically visible changes (Figures S29–S31, respectively).

## 3 Discussion

Our overall aim here was a comprehensive approach to identify extracellular and intracellular barriers for the delivery of SSO and how to overcome these by vectorization with the biocompatible carrier cLPEI. Intracellular delivery, in vivo pharmacokinetics, and both in vitro and in vivo splice correction efficiency of SSO were studied by applying a very broad range of techniques to identify any potential bottlenecks, which could otherwise preclude successful splice correction within the target tissue. While chemical modifications can improve the stability of SSO, vectorization is capable of changing their pharmacokinetics and enhancing the tissue cellular uptake.^17^ Lipid nanoparticles,^18^ peptides together with cationic lipids,^19^ or dendrimers^20^ improved the transfection efficiency in vitro, in vivo, and ex vivo. PS SSO can, due to its negative charge, electrostatically interact with polycations like PEI.^21^ Initial studies with plasmid DNA and LPEI of different MWs revealed that lower-MW LPEI 2 kDa does not induce any transgene expression.^8^ We employed low-MW LPEI 2.5 cross-linked with the homobifunctional cross-linker DSP, introducing intracellularly reducible disulfide bonds. The resulting cLPEI, due to its biocompatible and potentially biodegradable nature, should also allow systemic delivery in vivo because of enhanced bioavailability. A similar approach for cross-linking of lower-MW LPEI has first been described by Breunig and colleagues, who utilized the compound for in vitro transfection of plasmid DNA.^11^ Cross-linking of a low-MW polycation introducing disulfide bonds has meanwhile been unitized for delivery of not only plasmid DNA but also mRNA and siRNA.^22−24^ In the present manuscript, the goal was to exploit intracellular redox responsiveness of cLPEI for achieving highly efficacious SSO delivery to target organelles in vitro by overcoming multiple biological barriers via accelerated endosomal escape, high nuclear delivery, and functional splice correction. Further, its performance was tested in multiple in vivo models for demonstrating splice correction and nuclear accumulation of SSOs at organ- and cellular-levels in vivo. We measured a rather low degree of cross-linking, i.e., one out of 12 LPEI molecules is cross-linked. No significant influence on the nucleic acid-binding capability was observed: SSO retardation occurred between N/P 3 and 6, which is in line with the observations made by others.^25^ Due to the low cross-linking ratio, no major reduction of positively charged amines occurred, which are mainly responsible for retardation. We further used polyplexes with an excess of cLPEI at N/P 9, where particles were considerably small (<100 nm, also at high in vivo concentrations), remained stable over time, and were bearing a net positive charge. PS SSOs have been described to bind to stabilin-1 and −2 and are then internalized via clathrin-mediated endocytosis.^26^ In contrast, polyplexes with a net positive surface charge bind to heparan sulfate proteoglycans and are subsequently internalized via syndecan-actin triggered membrane internalization.^27^ Once internalized, release from the endosome is a major obstacle in nucleic acid therapeutics. While for PS SSO, intrinsic cellular mechanisms have been described involving distinct proteins,^28,29^ release is slow and might be less efficient when compared to transfection enhancers. Also, protection from enzymatic degradation in the late endosome/lysosome has to be considered. We could unambiguously demonstrate that plasmid DNA transfected with LPEI remains intact in endosomes also after prolonged incubation (24 h), whereas degradable polycations, like poly-l-lysine do not protect from degradation.^30^ Albeit the 2′-*O*-methyl (2′OMe) PS SSOs used in this study are less prone to enzymatic degradation, they can still be degraded in endosomes/lysosomes^31^ pointing out the importance of using transfection enhancers for SSO. Both limited or delayed endosomal release and enzymatic degradation can explain why 2′OMe-PS SSO only reach the nucleus in significant numbers when rather high concentrations are applied.^32^ While there was no major difference in biophysical properties between LPEI 2.5 and cLPEI polyplexes, intracellular performance was significantly different. Two hours after incubation, cLPEI polyplexes triggered the rupture of significantly more endosomes when compared to LPEI 2.5, whereas after 4 h, this difference was less pronounced. At this time (4 h), there was also no difference in the number of SSO positive nuclei or the fluorescence intensity per nucleus when using either of the polycations, but with LPEI 2.5, there was apparently more fluorescent signal remaining in the cytoplasm. We hypothesize that due to accelerated endosomal escape by cLPEI polyplexes (when compared to LPEI2.5k), the SSO-750 is present less in the endo/lysosomal compartments in case of cLPEI and thereby less visible in the extra-nuclear areas of the cell after 4 h of intracellular processing (Figure S9). In the case of LPEI 2.5k, the proportion of SSO-750 within endo/lysosomal compartments is thus more due to slower endosomal escape (when compared to cLPEI), thereby giving more signal in the “outside nucleus” area, as is visible in Figure S9. This was then quantitatively validated as shown in Figure S8C. As the total amount of SSO is the same in both polymer treatments, once cLPEI ensures higher endosomal escape, SSO bioavailability after endo/lysosomal processing would be higher for cLPEI, and this might translate, in principle, into higher nuclear delivery and higher splice correction. As it is difficult to quantify the throughput of “functional SSOs” coming out of endo/lysosomal processing via imaging, we compared the “splice correction status” at early intracellular processing time points, which then indirectly indicated functional nuclear delivery, as is presented in Figure 5C. It is clearly visible that cLPEI outperforms LPEI 2.5k significantly with log unit differences, indicating better nuclear delivery of splice-functional SSOs (Figure 5C). Thus, enhanced uptake and accelerated endosomal release also translated into strongly improved splicing activity leading to functional protein, demonstrating the efficient crossing of multiple biological barriers. We evaluated the splice correction efficiency using the well-established HeLa pLuc 705 cell line, which allows sensitive and quantitative analysis of splice correction.^15^ Our in vitro SSO concentrations used are somewhat higher (160 and 80 pmol/well correspond to 800 and 400 nM, respectively) compared to related work, where concentrations of 50 and 100 nM were used but rather high concentrations of transfection enhancers.^21,33^ Here we aimed at using a rather low N/P ratio of 9 (corresponding to an SSO/PEI w/w ratio of 1/1.17), which should also be applicable for systemic delivery in vivo. Also, the splice correction efficiency does not necessarily correlate between in vitro and in vivo, where several other factors are involved influencing the process of splice correction.^34^ Not unexpectedly, polyplexing allowed efficient splice correction at both concentrations, whereas naked SSO remained inactive, correlating with the differences in cellular uptake. Intriguingly, despite similar nuclear uptake, cLPEI triggered a 2 to 10-fold higher splice correction efficiency when compared to non-cross-linked LPEI 2.5. We conclude from these observations that the intracellular release kinetics plays a decisive role in the success or failure of SSO delivery. Concerning the mechanism, we can at least speculate that the earlier release from the endosome might halt the degradation of SSO, and hence, a higher fraction of SSO released to the cytoplasm enters the nucleus in a fully active state.

To investigate the splice correction activity of cLPEI within an in vivo setting, we developed a transgenic reporter mouse line carrying the same pLuc 705 in all organs and tissues as in HeLa pLuc 705 but under the control of the constitutive active ROSA26 promoter. Generation of transgenics by pronuclear injection of the (linearized) expression cassette into the male pronucleus can lead to concatemerization or mosaicism, where the latter is responsible for varying transgene expression levels in distinct organs/tissues.^35,36^ Nevertheless, we demonstrated stable integration in all transgenic generations and also detected pLuc 705 mRNA in all tested organs. Still, some variations in the background luciferase activity of untreated transgenic mice were observed, which can be due to intrinsic splice correction. Hence, in further studies, we always used scrambled control SSOs as a comparison treatment control. Of note, we did not include naked SSOs for in vivo splice correction studies. With such low doses and single injection of SSOs, usually no splice correction can be expected as has been already indicated in the literature (e.g., in Bauman et al. with four injections of 2.4 or 10 mg/kg naked SSO^18^). Due to this fact and the fact that we are bound to the 3R-principle when conducting animal experimentation, we did not include the naked SSO group. With a single dose of 5 mg kg^–1^ cLPEI-complexed SSO, we could visualize functional restoration of luciferase by BLI in explanted organs, i.e., heart, lung, liver, spleen, kidney, and bladder. Of note, we could not unambiguously detect a BLI signal in living mice when compared to control, i.e., scrambled SSO-treated animals. We expect this to be due to the lower enzymatic activity of the splice-corrected luciferase version and the absorption of photons emitted by the superficial tissue and skin layers. In the p705 vector, the wild type version of the luciferase gene is used^15^ and for sake of comparison, we used the same expression cassette to generate the transgenic reporter mice. To obtain a quantitative measure of splice correction, we have, after thorough optimization of the RNA isolation and RT-PCR protocol, analyzed the level of splice correction in respective organs.^37^ High levels of aberrant:corrected splice ratio (>1 in multiple mice) were achieved with cLPEI in liver and lung, meaning we could achieve >50% splice correction in those organs. In most organs, we also found a positive correlation between functional luciferase protein (BLI activity) and splice correction when treating with cLPEI/Luc-SSO. We have chosen only one time point to measure luciferase splice correction, i.e., 48 h. Persistence of splice correction depends also on the type of SSO used (e.g., up to a month using Locked-nucleic-acid based SSO^38^), although this is also dictated by the target, as in tumor tissue splice correction with a fast onset, but limited duration might be sufficient in the case of rapidly dividing cells. Recently, Dang and colleagues could demonstrate very effective splice correction in lung and other organs after systemic application of a cationic, cell-penetrating peptide conjugated, morpholino-based SSO in the GFP-654 transgenic mouse model.^39^ When combined with a small molecule-based oligonucleotide enhancing compound,^40^ the splice correction rate could be boosted to approximately 25%. This also points to the importance of proper vectorization to achieve significant splice correction also at lower doses.

We expect several factors that allow this rather high level of splice correction and first evaluated the pharmacokinetic profile. Here, vectorization with cLPEI considerably decreased secretion via the kidney. Using FLIT, we could also unambiguously identify organ accumulation of both naked and formulated SSO already 1 h after injection, namely, liver and kidney. This is also in line with studies using radiolabeled antisense (AS) oligonucleotides and SPECT, where liver and kidney accumulation was shown 3 h after subcutaneous injection of 100 mg kg^–1^ of AS oligonucleotides.^41^ It has been demonstrated that liver nucleases can also efficiently degrade PS SSO.^42^ When applied subcutaneously, naked PS SSO were mainly found within endolysosomes of macrophages (as observed for heart and liver) and tubular cells (in kidney), without reaching the nucleus.^43^ We were also investigating if our vectorization improves intracellular distribution within organs, and we first investigated the liver tissue. The considerable accumulation in hepatocyte nuclei could confirm that proper intracellular trafficking is also important for in vivo splice correction. SSO-NC-750 delivery to hepatocyte nuclei was demonstrated by first observing the SSO-NC-750 signal in liver in ex vivo organ imaging, as shown in Figure 10C. Then NIR-FLM was performed on liver cryo-sections (Figure S22) for cellular level visualization, where SSO-NC-750 signal colocalized with the nuclear DAPI signal of hepatocytes in all three injected mice, as shown in Figures 10D and S23, confirming delivery of SSOs to nuclei in hepatocytes. To investigate if passive accumulation of SSO in the tumor tissue can in principle be achieved with cLPEI in Balb/c mice bearing intraperitoneally disseminated CT26 colon cancer tumors, tumor lesions were investigated for potential SSO accumulation. The murine CT26 colon cancer carcinomatosis model using firefly luciferase-labeled CT26 cells has been used by us and other laboratories for therapeutic studies.^44^ As these cells grow quite aggressive, leading to a multitude of intraperitoneal lesions, two rounds of intraperitoneal oxaliplatin are needed to reduce the total number of tumor nodules, making them better imageable and trackable longitudinally in a noninvasive manner.^44^ Also, here, SSO could be detected in tumor lesions within the pancreas, which are reportedly well vascularized and can in principle be reached by the parenteral route.^45^ SSO-NC-750 signal was observed in pancreatic tumor nodules (Figure 10C), which was validated by the presence of the SSO-NC-750 signal in pancreatic nodule cryo-sections by NIR-FLM analysis (Figure 10E). However, in contrast to liver tissue, no clear nuclear accumulation was seen. Among other organs, although lung accumulation of cLPEI-SSO polyplexes was observed in all three tested mice (Figure S24), no nuclear release was seen at the investigated time point, which is a critical requirement for therapeutic splice correction. Tumor heterogeneity in this model^45^ in vascularization could be one of the reasons for lack of SSO accumulation in individual tumor nodules, as has been observed in other studies.^46^ Here, the total amounts of SSO and cLPEI taken up by the cell might have been limited, as reportedly also free LPEI can promote the endosomal release process.^47^ For this, additional approaches, i.e., the implementation of targeting ligands is necessary.^48,49^ We and others have successfully utilized the epidermal growth factor receptor (EGFR) as a viable target to enhance nucleic acid delivery to tumors.^50^ Also, for colon cancers, this would be applicable, and we aim to follow this up with our recently developed EGFR-targeting peptides with improved targeting ability together with shielding polymers like PEG to improve the circulation time and tumor accumulation.^51^ Concerning potential toxic effects with cLPEI, we did not observe any acute response in any of the treated animals. In tumor-bearing Balb/c mice, no morphological changes could be observed in any of the investigated organs. PEI has so far been used successfully also in many human clinical trials without exhibiting negative side effects.^52^ Nevertheless, the final fate of internalized PEI has not yet been clarified in detail. Initial studies by Lecocq et al. indicated that it remains in the liver within the lysosomal compartment,^53^ although this also depends on the MW and branching degree of the PEI used. Depending on the uptake route, endosomal/lysosomal content could in principle also be recycled and secreted again by the cell, as assumed for the nucleic acid part.^40^

## 4 Conclusions

Exon skipping is applicable not only for monogenic diseases but also other malignancies including cancer.^54^ The polycation cLPEI presented in this study allows to reduce the effective in vivo dose by more than an order of magnitude, thus bringing it into a more appropriate range for further clinical applications and potentially preventing side effects of high SSO doses like inflammation and nephro- and hepatotoxicities, which are especially of concern when considering the necessity of multiple or continuous dosing.^55^ This study also highlights the importance of monitoring all biological barriers and enhancing SSO bioavailability to the splicing machinery for overcoming the challenges of RNA-targeted therapeutics. An additional layer of selectivity can be the active targeting of vectorized nucleic acids. With a simple coupling chemistry, cell-targeting peptides can be covalently attached to PAMAM dendrimers^48^ or LPEI,^51^ enabling and improving the cell-targeting effect. Such technologies can be easily adapted to further improve the splice-correcting activity of SSO.

## 5 Experimental Section

### 5.1 Materials

All oligonucleotides used in this study were kindly supplied by GlaxoSmithKline (GSK, UK). Both luciferase-correcting SSO (SSO-Luc) and scrambled negative control (SSO-NC)oligonucleotides were 2′OMe modified and synthesized as phosphorothioates with the following sequences: SSO-Luc: 5′-CCUCUUACCUCAGUUACA-3′ (*M*_W_ = 6472.61 Da); SSO-NC: 5′-CUUGUUAUACCACUUACA-3′ (*M*_W_ = 6496.64 Da). Labeled scrambled control oligos were generated by coupling *N*-hydroxysuccinimide-functionalized fluorescent dyes (AF647 or AF750) to the 3′ end of the oligonucleotide.

All reagents used, if not mentioned otherwise, were purchased from Sigma-Aldrich (Darmstadt, Germany). Type 1 pure water (Milli-Q water) was used in all applications, purified using Sartorius Arium Pro (Sartorius, Göttingen, Germany).

### 5.2 Synthesis and Characterization of LPEI and cLPEI

LPEI2.5 was purchased from Polysciences (art. no. 24313, Polysciences Europe, Hirschberg, Germany) and analyzed similarly. LPEI was then cross-linked using dithiobis(succinimidyl propionate) (DSP, Lomant′s reagent) in a molar ratio of 2 LPEI (83.33 μmol):1 DSP (41.5 μmol) and termed as cLPEI. Synthesis was run under water-free conditions in absolute ethanol at 50 °C under constant stirring overnight. Free base was precipitated using sodium hydroxide solution (10 M) and washed until neutral using Milli-Q water. Remaining unreacted linker was removed via ion exchange chromatography using cation exchange resin (MacroPrep High S, Biorad, Vienna, Austria) with a linear gradient of sodium chloride (0.5–3 M) over 30 min (ÄKTA pure FPLC, GE healthcare, Vienna, Austria). Fractions containing cLPEI were pooled and dialyzed against water using 1 kDa MWCO tubing (Spectra/Por 6, ThermoFisher, Regensburg, Germany).

Degree of cross-linking was evaluated according to eqs 1 and 2:
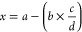
1where *x* is the molar amount of thiols stemming from disulfides after reduction, *a* is the amount of free thiols calculated from a standard curve, *b* is the molar amount of thiols before reduction (i.e., not taking part in cross-linking), *c* is the molar amount of LPEI present before reduction, and *d* is the molar amount of LPEI after reduction. This is expressed as the degree of cross-linking in percent following eq 2:
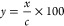
2

Thiols were quantified using Ellman’s assay after reducing cLPEI with resin bound 3,3′,3″-phosphanetriyltripropanoic acid (immobilized TCEP-reducing gel, ThermoFisher) following the vendor’s protocol. After 1 h of reaction time at 50 °C, the amount of free thiols was calculated from a standard curve (a). To exclude free thiols present in the sample before reduction, and therefore an overestimation of cross-linking, an Ellman’s assay was also run from the same sample before adding TCEP resin (b). LPEI was quantified in the sample before (c) and after reduction (d) using the copper assay.^47^ This was necessary as there was 55–70% less sample present after reduction as some cLPEI remained bound to the resin (for details see Figure S1). LPEI 10, i.e., higher-MW polyethylenimine was synthesized from poly(2-ethyl-2-oxazoline) by acidic hydrolysis, as described previously.^49^

#### 5.2.1 SSO Polyplex Generation

Polyplexes were prepared by mixing SSO-Luc or SSO-NC and the indicated polymer, i.e., LPEI 2.5 or cLPEI, by flash pipetting technique, as described earlier.^11^ In brief, the needed amounts of the desired polymer and nucleic acid were diluted separately in HBG (pH 7.4) and mixed at the desired N/P ratio. This ratio describes the molar ratio of nitrogen groups (N) in polyethylenimine subunits compared with phosphate groups (P) in the nucleic acid. The prepared polyplexes were then immediately used for the intended experiments.

### 5.3 Gel Retardation Assay for SSO Complexation

cLPEI-SSO-NC-647 polyplexes were prepared at 60 μg mL^–1^ SSO at N/P ratios of 3–12 as described above. Polyplexes containing SSO (200 pmol) were then mixed with 10% glycerol and loaded per well in a 1.5% agarose gel for electrophoresis. SSO complexation was compared between cLPEI and LPEI 2.5 by mixing SSO-NC with cLPEI or LPEI 2.5 at N/P 9 and subjecting it to agarose gel electrophoresis. The gel was run for 1 h at 80 V and imaged using ChemiDoc XRS+ Imaging System (BioRad Laboratories Inc., California USA).

### 5.4 Size and Zeta Potential Measurement of SSO Polyplexes

Polyplexes were prepared at 20 μg mL^–1^ (size), 200 μg mL^–1^ (zeta), and 400 μg mL^–1^ (in vivo) SSO concentrations at N/P ratio 9. Size of the formed nanoparticles was evaluated by NTA and zeta by dynamic light scattering; both are described elsewhere.^11^ In brief, polyplexes were generated as described above and then diluted in HBG buffer to give a concentration range of 10^8^–10^9^ particles per mL, i.e., 10–100 particles in the field of view (FOV). These samples were finally measured using NTA with a 488 nm laser on a NanoSight NS500 (Malvern Panalytical, Malvern, UK) instrument. For the zeta measurement, polyplexes were diluted in 2.5 mM NaCl to 1.0 mL and measured in the zetasizer NANO ZS (Malvern). Results were analyzed using Zetasizer Software 7.13 (Malvern) exporting only good quality ones as per the software.

### 5.5 Effect of Serum on SSO Complexation

SSO-NC oligos were prepared in HBG buffer (naked) or polyplexed using cLPEI at N/P ratio 9 and directly incubated with fetal calf serum (FCS) or HBG buffer for 30 min and 4 h, respectively. Each sample (400 ng) was run on a 0.75% or 1.5% agarose gel stained with EtBr. Images were taken at 3 s exposure time. Effect of serum on polyplex stability was compared between cLPEI and LPEI 2.5 based SSO-NC polyplexes by incubating N/P 9 polyplexes in FCS or HBG for 30 min and subjecting to agarose gel electrophoresis.

### 5.6 Cell Culture

HeLa pLuc 705 cells^12^ (obtained within the IMI collaborative project COMPACT, grant agreement no. 115363) were grown in Dulbecco’s modified Eagle medium (DMEM) high glucose supplemented with 10% FCS (Biowest, Riverside, MO, USA), 1% penicillin-streptomycin (Sigma-Aldrich), 2% l-glutamine (Sigma-Aldrich), and 200 μg mL^–1^ hygromycin B. HeLa wild-type (CCL2, ATCC, Manassas, USA) and HeLa mRuby-3 galectin 8 cells (see section 5.7) were grown in the same medium without hygromycin B. CT26-Luc cells were cultured in DMEM/F12 medium (1:1) supplemented with 10% FCS, 1% penicillin-streptomycin, and 1% l-glutamine; luciferase expression was confirmed on the day of injection using the GloMax Navigator System (GM2010, Promega). Medium without any supplements added is later referred to as the basal medium (BM), and supplemented medium is referred to as the complete medium (CM). All cell lines were kept under standard cell culture conditions at 37 °C in a humidified atmosphere with 5% CO2.

### 5.7 Endosomal Escape and Nuclear Entry Studies in HeLa mRuby-3 Galectin 8

HeLa cells with stable expression of mRuby-3 galectin 8 from the plasmid PB-CAG-mRuby-3-Gal8-P2A-Zeo (gift from Jordan Green; Addgene plasmid #150815; http://n2t.net/addgene:150815; RRID:Addgene_150815) were used.^13,14^ HeLa mRuby-3 galectin 8 cells were detached using Tryp-LE (Gibco Tryp-LE express enzyme 1X phenol red) as per vendor’s protocol, resuspended in CM, and counted using a Neubauer improved hemocytometer (Paul Marienfeld GmBH & Co. KG). Cells were seeded in flat transparent 96-well plates (Greiner CELLSTAR, Greiner Bio-One, Kremsmünster, Austria) at a seeding density of 10 000 cells per well and incubated for 24 h. CM was removed, and different treatments were added in the BM to reach a total volume of 100 μL per well. The different treatments were 80 or 160 pmol cLPEI-SSO-NC-750 or LPEI2.5-SSO-NC-750 (all N/P 9) or naked SSO-NC-750. As a negative control, HBG was used, while chloroquine was the positive control for endosomal escape. After 2 or 4 h of incubation, the medium was removed, and the cells were washed twice with Dulbecco’s phosphate-buffered saline (DPBS) and fixed using 4% (w/v) paraformaldehyde for 40 min. Cells were washed two more times, and nuclei were stained using DAPI at 2 μg mL^–1^ for 20 min. After washing, cells were covered in DPBS (100 μL) and imaged with a 20-fold magnification on an Olympus IX73 inverted microscope with an Olympus XM10 digital microscope camera (Olympus K.K., Shinjuku, Tokio, Japan). Images of three FOVs were taken for each well. Nuclei were visualized with an exposure time of 400 ms, while for Gal8 clusters, an exposure time of 2 s was chosen. AF750 was imaged using an acquisition time of 100 ms.

#### 5.7.1 Quantitative Analysis of Endosomal Escape

For each FOV, the number of Gal8 clusters was normalized by the number of nuclei; both were counted using ImageJ via Fiji (v. 1.53a, fiji.sc). First, the RGB image of Gal8 clusters was converted into a 16-bit grayscale image, the background was subtracted to reduce the blur, and the Gal8 clusters were defined as regions of interest by setting an intensity threshold. Overlapping clusters were separated into individual ones using the watershed function, and all clusters were counted. Similarly, the RGB image of nuclei was converted into a 16-bit grayscale image as described above. The intensity threshold was adjusted, and the overlapping nuclei were separated as described. Finally, the nuclei were counted, and the average number of Gal8 clusters per cell was calculated.

#### 5.7.2 Quantitative Analysis of Nuclear Entry

The percentage of AF750-positive nuclei was calculated by first counting the nuclei, as described above. The number of nuclei containing an AF750 signal above a certain intensity was then identified. Therefore, a lower threshold was chosen for each image individually to select nuclei with a relatively high AF750 signal. To include only whole nuclei, the count was limited to roughly circular objects. Finally, the percentage was calculated.

Additionally, the ratio of the mean AF750 intensity within nuclei to the mean AF750 intensity outside nuclei was calculated as follows: To obtain the mean intensity within nuclei, the DAPI image was first converted into a 16-bit greyscale image. An intensity threshold was then set to define the nuclei, a dark background chosen, and the selected nuclei defined as regions of interest. Then, the 16-bit AF750 image was overlaid with the outlines of the nuclei, and the mean intensity was measured within the nuclei. For determining the mean intensity outside the nuclei, the same steps as above were repeated, with the exception that a white background was selected to limit the measurement to the outside. Finally, the ratio of the mean AF750 intensity within nuclei to the mean AF750 intensity outside nuclei was calculated.

### 5.8 CLSM-Based Nuclear Delivery in HeLa pLuc 705

The study was carried out in principle as described previously.^35^ In brief, 20 000 HeLa pLuc 705 cells were seeded in 8-well chamber slides (Nunc Lab-Tek II, Cat No: 155409; ThermoFisher) 24 h prior treatment. The medium was changed to BM, and the cells were treated with 0.8 μM SSO-NC-647 polyplexes (160 pmol, N/P 9) for 4 h. CM was added, and the cells were incubated for 20 more hours. Then, the cells were washed three times with DPBS, fixed using 4% (w/v) paraformaldehyde for 40 min, permeabilized with 0.1% Triton × 100 for 5 min, and washed two more times. Actin was stained using AF488-phalloidin (ThermoFisher) as per vendor guidelines and washed three times, and subsequently, nuclei were stained using DAPI at 2 μg mL^–1^ for 5 min. Last, each well was covered with DPBS (200 μL) and directly imaged on a Leica TCS SPE microscope (Leica, Germany). DAPI was excited using a 405 nm laser, phallodin-AF488 with 488 nm, and SSO-NC-647 at 635 nm. Signal detection parameters were set initially for all fluorophores and each magnification separately and then kept constant for all samples investigated across the whole experiment. For each treatment, Z-stacks of three FOVs were recorded using a 20× air and 63× oil immersion objective. Z-stacks were ranging from top to bottom of visible AF488-phalloidin signal in each FOV with a slice resolution of 2 μm and 340 nm each, respectively. Data were acquired using LasX software (version 3.1.2.16221) and analyzed using FIJI.

### 5.9 In Vitro Splice Correction in HeLa pLuc 705 by Firefly Luciferase Assay

Splice correction was studied via the firefly luciferase assay of HeLa pLuc 705 cells. In brief, 10,000 cells per well were seeded in white flat-bottom 96-well plates (Greiner) 24 h before treatment. Media was exchanged to BM, and the cells were treated with SSO-Luc or SSO-NC based polyplexes. However, the treatment dosage was kept the same for each treatment. Noncomplexed naked SSOs were included as a control treatment. After 4 h, CM was added, and the cells were left to incubate for a total of 24 h of treatment. Afterward, the cells were washed once with DPBS and lysed using passive lysis buffer (PLB 5×, Promega, Mannheim, Germany, 30 μL), and firefly luciferase assay was performed using 100 μL of LBL buffer on a Promega GloMax, as previously described.^49^ For the 4 h time point, cells were left in BM; for the 6 h time point, CM was added after 4 h. However, after the incubation time, cells of the respective plate were washed using DPBS and covered in 30 μL of DPBS. Luminescence was directly measured as described. For the splice correction read-out, the splice correction fold increase was calculated by dividing the RLU from SSO-Luc by the RLU from SSO-NC for the same type of treatment.

Splice correction and cell viability profile of SSO polyplexes based on cLPEI, LPEI 10, and lipofectamine 3000 was investigated by firefly luciferase assay and resazurin assay as described below. HeLa pLuc 705 cells were seeded as described above 24 h prior to treatment. Media was exchanged to BM, and the cells were treated with SSO-Luc or SSO-NC based polyplexes (N/P 9) of different formulations: cLPEI, LPEI 10, or lipofectamine 3000 (according to manufacturer’s protocol). HBG was included as a buffer control. After four h, complete medium was added, and the cells were further incubated. Powdered resazurin (Sigma-Aldrich, ST. Louis, MO, USA) was dissolved in DPBS to reach a final concentration of 0.1 mg/mL and sterile filtered. After 23 h of incubation with polyplexes, the medium was partly removed, and resazurin solution with a final concentration of 0.01 mg/mL was added. Wells without cells but CM were treated the same way and used as a blank. Cells were incubated for another hour with polyplexes to complete 24 h of treatment schedule, after which the supernatant was resuspended and transferred to a transparent 96-well plate (Cell Star, Greiner Bio-One, Kremsmünster, Austria) for reading metabolic activity via resazurin conversion. Cells were washed using DPBS and covered in 30 μL of DPBS and used for firefly luciferase assay. Luminescence was directly measured as described above. For splice correction read-out, splice correction fold increase was calculated by dividing the RLU from SSO-Luc by the RLU from SSO-NC for the same type of treatment. As resazurin assay read-out, fluorescence was measured on a Tecan infinite M200PRO (Tecan Trading AG, Mannedorf, Switzerland) with an excitation of 560 nm and an emission maximum of 590 nm.^56^

### 5.10 Cell Association and Uptake of SSO Polyplexes by Flow Cytometry

HeLa pLuc 705 cells were detached using Versene (ThermoFisher) as per vendor’s protocol, resuspended in DPBS (ThermoFisher) and counted using a MACSQuant Analyzer 10 (Miltenyi Biotec, Bergisch Gladbach, Germany) for seeding. HeLa pLuc 705 cells were seeded in a flat transparent 96-well plate (Greiner CELLSTAR, Greiner Bio-One, Kremsmünster, Austria) at a seeding density of 35,000 cells per well 24 h before treatment, and the medium was exchanged to BM. Polyplexes (40 or 80 pmol) from the intended polymer were prepared at indicated N/P ratios and added to each well resulting in 0.2 and 0.4 μM final concentration of the formulation. Cells were automatically stained using DAPI (1 μg mL^–1^ final concentration), gated for main cell population using FSC-A vs SSC-A, and then live–dead gated using FSC-A vs V1-A (ex. 405 nm, 450/50 nm band-pass filter), and geometric mean in channel R1 (ex. 635 nm, 693/38 nm band-pass filter), corresponding to AF647 dye, was recorded. Data analysis was done using FlowJo v. 10.8.1 (FlowJo LLC, Ashland, OR, USA).

### 5.11 Animals

For all in vivo experiments, mice were kept in individually ventilated cages (Type 2L, Tecniplast, Hohenpeißenberg, Germany) under specified pathogen-free conditions according to FELASA recommendations (room temperature 21 ± 1 °C [mean ± SEM]; relative humidity 40%–55%; photoperiod 12L:12D) and water and food (standard rodent diet; SSniff, Soest, Germany) were provided ad libitum. The welfare of animals was assessed daily. All procedures involving animals were discussed by the institutional ethics committee at the University of Veterinary Medicine Vienna and the University of Vienna, and approved by the Austrian Federal Ministry for Education, Science and Research (approval numbers BMWF-68.205/0023-II/3*b*/2014 and BMWF-66.006/0010-WF/V/3*b*/2016). Minimum 10 days prior to any imaging experiment, food was switched to low-fluorescent diet (EF AIN 76A rodent diet, Purified Diet, Ssniff Spezialdiäten, Soest, Germany).

### 5.12 Split-Luciferase Transgenic Mouse Model Generation and Characterization

The split-luciferase transgene was provided by Life Technologies by assembling synthetic oligonucleotides and/or PCR products as per the sequence information on split-luciferase gene in the already reported Hela pLuc 705 cell line.^12,49^ The provided split-luciferase transgene, interrupted by human β-globin intron 2 (GenBank accession number L48219.1; 664..1512 (intron), T1367G), was flanked by SalI restriction sites and cloned under the human ROSA promoter into pBROAD2-mcs plasmid (Invivogen, Toulouse, France) by SalI based cloning. pBROAD-mcs is optimized for mouse transgenesis and ensures transgene expression ubiquitously in virtually all tissues. The plasmid also contains the human beta-globin 3′ UTR and polyadenylation sequence for efficient transgene transcription. The cloned plasmid was expanded in *E. coli* K12, purified, and analyzed by diagnostic restriction digest. Five μg of plasmid was digested with NdeI and NsiI, the restriction digest fragments (backbone and insert) were separated by agarose gel electrophoresis, the 5839 bp band, including the promoter, split-luc cDNA, and poly-A site, was cut from the gel, and the DNA was purified. The DNA construct was microinjected into a male pronucleus of fertilized eggs of C57BL/6N mice (Janvier Laboratories, Le Genest-Saint-Isle, France) and transferred to pseudo-pregnant SWISS mice as described.^33^ Three detected founder animals (nos. 739, 740, and 741, all males) were backcrossed twice to C57BL/6N-*Tyr*^*c*^ (Charles River, Wilmington (MA), USA) to obtain transgenic mice with albino background.

#### 5.12.1 PCR-Based Genotyping

For genotyping, mouse ear punches were used for DNA extraction by using the GeneJET Genomic DNA Purification Kit (K0722, ThermoFisher) following vendor’s protocol. Purified genomic DNA (gDNA) was quantified via NanoVue Plus photometer (Biochrom, Cambridge, UK), and only samples with a A260/A280 quality factor >1.7 were used. For PCR, gDNA (40 ng) was amplified in DreamTaq Green PCR Master Mix (ThermoFisher) using the following primer set: 5′-TAATGAACGTGAATTGCTCAACAGT-3′ LSP forward and 5′-TGGTAATCCGTTTTAGAATCCATG-3′ LSP reverse. Samples were analyzed in sodium borate buffer (12.5 mM sodium hydroxide, 80.5 mM boric acid, pH = 8) gel, stained with SYBRsafe (ThermoFisher), imaged with ChemiDoc XRS+ Imaging System (BioRad), exposed to detect faint signal bands, and analyzed using ImageLab Software (Biorad, v.6.0.1).

#### 5.12.2 RT-PCR-Based Organ Characterization for the Presence of pLuc 705 mRNA

##### 5.12.2.1 RNA Extraction and Quality Control

RNA was extracted as described below, and RNA quality was evaluated via gel electrophoresis. Mouse organs stabilized against nuclease degradation were taken, blotted dry, flash frozen, and ground in liquid nitrogen. Whole RNA was extracted and purified, including on-column DNA digestion, using RNeasy mini kit and RNase-Free DNase Set (both Qiagen, Hilden, Germany) following vendor’s instruction. Purified whole RNA was evaluated for degradation via both UV–vis ratios of A260/A280 and A260/A230 (NanoVue Plus photometer, Biochrom) and gel electrophoresis. Here, total RNA (0.5 μg) was loaded in 1% agarose gel in TAE buffer (2-amino-2-(hydroxymethyl)propane-1,3-diol (Tris) [400 mM], acetate [400 mM], ethylenediaminetetraacetic acid [10 mM], stained with ethidium bromide (EtBr, 30 μg) and run for 45 min at 100 V constant voltage. Images of the gels were taken using ChemiDoc XRS+ Imaging System (BioRad) at 0.5 s and analyzed utilizing ImageLab Software (Biorad, v.6.0.1). As reference, an RNA ladder (RiboRuler High Range RNA ladder, ThermoFisher) was used. For quality control, the intensity of the 28S rRNA band (between 4000 and 6000 b ladder fragments) was divided by the 18S rRNA band (between 1000 and 1500 b). All equipment employed for RNA extraction was thoroughly decontaminated using RNase AWAY (ThermoFisher) or heated to 240 °C for 4 h minimum. RT reactions were carried out as per vendor’s instructions, and then cDNA (200 ng) was amplified using the same PCR protocol. cDNA of isolated organs was used for an organ level characterization for the presence of pLuc 705 mRNA by using LSP primers.

#### 5.12.3 2D Bioluminescence Imaging-Based Organ Characterization for Background Luminescence

Based on genotyping outcome, split-luciferase transgene-positive and -negative mice were subjected to bioluminescence imaging for investigating background luminescence signals of organs of interest. Toward this, the mice were injected with D-luciferin (Intrace Medical SA, Lausanne, Switzerland, 120 mg kg^–1^ body weight) in DPBS into the right abdominal side subcutaneously (s.c.) while anesthetized with isoflurane (2–2.5%) in oxygen. Mice were euthanized by cervical dislocation and organs placed into the IVIS Spectrum CT [PerkinElmer, Waltham (MA), USA]. Images were acquired with open filter settings and subject height set to 1.5 cm. Imaging analysis was done as described recently.^34^ In brief, regions of interest (ROIs) were drawn over each organ, the radiance (photons) was selected as the measurement type, and the signal was quantified as average radiance (p/sec/cm^2^/sr) for quantification using LivingImage software (v. 4.5.2, PerkinElmer).

### 5.13 In Vivo Splice Correction in Split-Luciferase Transgenic Mice

Split-luciferase transgene-positive mice were treated with cLPEI-SSO (5 mg kg^–1^ SSO dosage) polyplexes via tail vein injection. SSO polyplexes were prepared as described above but at a final SSO concentration of 400 μg mL^–1^. After 48 h of SSO polyplex administration, the mice were euthanized and analyzed for splice correction in terms of functional luciferase protein by 2D bioluminescence ex vivo organ imaging as explained above and validated by RT-PCR for splice correction at the mRNA level, as detailed below.

#### 5.13.1 Splice Correction Validation at the mRNA Level by RT-PCR

Purified and quality controlled whole RNA was transcribed into cDNA using a High Capacity cDNA Reverse Transcription Kit (Applied Biosystems, ThermoFisher). cDNA (200 ng) was amplified using DreamTaq Green PCR Master Mix (ThermoFisher) with each following primer set individually: LSP forward/reverse, SSP forward: 5′-TTGATATGTGGATTTCGAGTCGTC-3′, SSP reverse: 5′-TGTCAATCAGAGTGCTTTTGGCG-3′ (as used in ref (36)), beta Actin (ActB) forward: 5′-GCACCACACCTTCTACAATG-3, ActB reverse: 5′-TGCTTGCTGATCCACATCTG-3′. Samples were transferred into a 2% agarose gel in SB buffer, EtBr stained, and run for 45 min at constant 100 V. Images were acquired on ChemiDoc XRS+ Imaging System (BioRad) at 0.1 s exposure time and analyzed using ImageLab Software (Biorad, v.6.0.1). Splice correction was calculated as the splice correction ratio by dividing the intensity of a corrected band (143 bp) by the aberrant splice band (290 bp) (i.e., a similar intensity of aberrant and corrected band results in a splice ratio of 1 corresponding to 50% splice correction; an aberrant band double the intensity of corrected band results in a splice ratio of 0.5 or 33.3% splice correction). The positive control used in all RT-PCR runs was prepared as previously published.^36^

### 5.14 NIR FLIT/X-ray CT-Based Spatiotemporal Tracking and 2D Fluorescence Ex Vivo Organ Imaging in Wild-Type Mice

For in vivo biodistribution of AF750-labeled SSOs, imaging workflow and analysis were followed as described previously.^14^ In brief, naked-SSO-NC-750 or cLPEI-SSO-NC-750 polyplexes (5 mg kg^–1^) were injected into anaesthetized B6(C)/Rj-*Tyr*^*c/c*^ mice (B6-albino, Janvier, 53941 Le Genest Saint Isle, France) via the tail vein as described above. Here, out of the total SSO dosage, only 20 μg of SSOs were labeled with the AF750 dye. Animals were left to recover and were released back to the cage. After 1 h, animals were again put under isoflurane anesthesia. Iopamidol (Scanlux, Sanochemia, Vienna, 120 μL), a CT contrast agent, in 5% glucose (ratio 1:2.5 v:v) was administered intraperitoneally (i.p.), and 2D FLI was performed in the epifluorescence mode. Subsequently, 3D FLI (FLIT/CT) was acquired, where FLI was executed in the transillumination mode after mice were CT scanned (low resolution). This was done in both supine and prone positions. Mice were again awakened and released in the cages. The same measurements were repeated after a total of 24 h after SSO injection. Thereafter, mice were euthanized, and ex vivo 2D fluorescence organ imaging was performed. For signal quantification, images were acquired with the following filter pairs for excitation/emission (nm): 640/680, 640/700, 640/720, 640/740, 640/760, 675/720, 675/740, 675/760, 675/780, 675/800.

### 5.15 Biodistribution in Colorectal Tumor-Bearing Animals

10^5^ CT26-Luc cells were injected i.p. into BALB/cJRj mice (Janvier, 53941 Le Genest Saint Isle, France) (D0). Cell count was measured using MACSQuant Analyzer 10 (Miltenyi Biotech, Bergisch Gladbach, Germany). Animals were injected with 6 mg kg^–1^ oxaliplatin twice (D3 and D6). Tumor presence was validated by BLI after a s.c. injection of luciferin into the neck area (Figures 10A and S15). On day 15, naked SSO-NC-750 or cLPEI-SSO-NC-750 polyplexes (5 mg kg^–1^) were injected via the tail vein into anesthetized animals. Animals were left to fully wake up and released back to the cage. After 4 h, iopamidol in 5% glucose (as described) was injected both i.p. and i.v. (150 μL each) into the anaesthetized mice. FLIT/CT was conducted as described above. Afterward, the mice received a second s.c. injection of luciferin and were euthanized. Organs were excised, and ex vivo BLI as well as FLI were conducted as stated before (Figure S16). Organs were embedded in cryomolds using Tissue-Tek O.C.T. (Sakura Finetek Europe B.V., Umkirch, Germany), frozen on dry ice, and stored at –80 °C until further processing.

Image analysis was performed following the published procedure. In brief, regarding whole-body BLI, one ROI which covered the abdominal area (from sternum to bladder) was created per mouse. Afterward, the average radiance was measured, and the data were exported. For FLIT analysis, based on the CT data, ROIs were placed around kidneys and liver before the reconstruction was loaded. Then, the fluorescent yield (pmol M^–1^ cm^–1^) was measured and exported. For the ex vivo analysis of FLI, the filter pair method was chosen. However, for both BLI and FLI ex vivo analyses, separate ROIs were drawn around individual organs; for BLI, average radiance was exported, while it was radiant efficiency ((p/sec/cm^2^/sr)/(μW/cm^2^)) for FLI.

#### 5.15.1 Histological Evaluation

Liver, lung, pancreas, and tumor were of interest for histological evaluation. Consecutive sections of mentioned organs were cut at a thickness of 6 μm and a temperature of -20 °C using a cryostat (MNT, SLEE medical GmbH, Nieder-Olm, Germany) (Figure S22B) and stored at -80 °C. Selected sections were fixated using 4% paraformaldehyde in DPBS and washed three times using DPBS.

#### 5.15.2 Histopathological Analysis

Two slides (slide 1 and 12) of each organ were stained with hematoxylin and eosin (H&E) for general histological evaluation of the tissue. After mounting in Entellan new (Merck KGaA, Darmstadt, Deutschland) mounting medium, whole-organ scans were acquired on an Olympus BX53 light microscope equipped with an Olympus DP-73 color camera.

#### 5.15.3 Nuclear entry within tissue by NIR-FLM

Two additional slides (slide 2 and 13) were incubated with 100 μL of DAPI for 30 min. After rinsing them twice using DPBS, they were mounted in a VECTASHIELD Antifade mounting medium (Vector Laboratories, Newark, CA, US). The slides were subjected to z-stack acquisition to visualize the NIR dye AF750 as well as DAPI within the same FOV using a 63× oil objective on an Olympus IX73 inverted microscope with an Olympus XM10 digital microscope camera attached. For each FOV, 31 stacks with a z-spacing of 0.3 μm were recorded, and deconvolution as well as maximum intensity projection were performed employing cellSens Dimensions (Olympus K.K., Shinjuku, Tokio, Japan).

### 5.16 Statistical Analysis

In Figures 2 and 9, groups were compared using a two-tailed *t* test. To evaluate different treatments in Figures 3–6, means and standard deviations were calculated across all three experiments. The means were then compared using Welch′s *t*-Test (https://www.statskingdom.com/150MeanT2uneq.html). https://rpkgs.datanovia.com/rstatix/ For all Figures: ns = not significant, * = *p* < 0.05, ** = *p* < 0.01, *** = *p* < 0.001, **** = *p* < 0.0001.

## Data Availability

Data will be made available on request.

## Supporting Information Available

Gel retardation data, NTA, serum stability data, FLM images of endosomal escape assay, FLM images and image-based quantification of SSO delivery, confocal laser scanning microscopy images, firefly luciferase assay data, flow cytometry analysis, evaluation of split-luciferase transgenic reporter mice for gene expression, basal bioluminescence signals and mRNA expression, workflow for RT-PCR and splice ratio/splice correction calculation, experimental setup of tumor biodistribution study and associated data for fluorescence imaging/X-ray absorption computed tomography, ex vivo fluorescence imaging, and NIR-FLM-based histological analysis (PDF)

## References

[ref1] Hanson B.; Wood M. J. A.; Roberts T. C. Molecular correction of Duchenne muscular dystrophy by splice modulation and gene editing. RNA Biol. 2021, 18, 1048–1062. 10.1080/15476286.2021.1874161. 33472516 PMC8216187

[ref2] Aartsma-Rus A.; Goemans N. A Sequel to the Eteplirsen Saga: Eteplirsen Is Approved in the United States but Was Not Approved in Europe. Nucleic Acid Ther 2019, 29, 13–15. 10.1089/nat.2018.0756. 30526286

[ref3] Frank D. E.; Schnell F. J.; Akana C.; El-Husayni S. H.; Desjardins C. A.; Morgan J.; Charleston J. S.; Sardone V.; Domingos J.; Dickson G.; Straub V.; Guglieri M.; Mercuri E.; Servais L.; Muntoni F.; Increased dystrophin production with golodirsen in patients with Duchenne muscular dystrophy. Neurology 2020, 94, e2270–e2282. 10.1212/WNL.0000000000009233. 32139505 PMC7357297

[ref4] Bost J. P.; Barriga H.; Holme M. N.; Gallud A.; Maugeri M.; Gupta D.; Lehto T.; Valadi H.; Esbjorner E. K.; Stevens M. M.; El-Andaloussi S. Delivery of Oligonucleotide Therapeutics: Chemical Modifications, Lipid Nanoparticles, and Extracellular Vesicles. ACS Nano 2021, 15, 13993–14021. 10.1021/acsnano.1c05099. 34505766 PMC8482762

[ref5] Crooke S. T.; Baker B. F.; Crooke R. M.; Liang X. H. Antisense technology: an overview and prospectus. Nat. Rev. Drug Discov 2021, 20, 427–453. 10.1038/s41573-021-00162-z. 33762737

[ref6] Roberts T. C.; Langer R.; Wood M. J. A. Advances in oligonucleotide drug delivery. Nature reviews. Drug discovery 2020, 19, 673–694. 10.1038/s41573-020-0075-7. 32782413 PMC7419031

[ref7] Wang Y.; Ye M.; Xie R.; Gong S. Enhancing the In Vitro and In Vivo Stabilities of Polymeric Nucleic Acid Delivery Nanosystems. Bioconjug Chem. 2019, 30, 325–337. 10.1021/acs.bioconjchem.8b00749. 30592619 PMC6941189

[ref8] Godbey W. T.; Wu K. K.; Mikos A. G. Size matters: molecular weight affects the efficiency of poly(ethylenimine) as a gene delivery vehicle. J. Biomed Mater. Res. 1999, 45, 268–275. 10.1002/(SICI)1097-4636(19990605)45:3<268::AID-JBM15>3.0.CO;2-Q. 10397985

[ref9] Hall A.; Lachelt U.; Bartek J.; Wagner E.; Moghimi S. M. Polyplex Evolution: Understanding Biology, Optimizing Performance. Mol. Ther 2017, 25, 1476–1490. 10.1016/j.ymthe.2017.01.024. 28274797 PMC5498806

[ref10] Zhang L.; Yu M.; Wang J.; Tang R.; Yan G.; Yao W.; Wang X. Low Molecular Weight PEI-Based Vectors via Acid-Labile Ortho Ester Linkage for Improved Gene Delivery. Macromol. Biosci 2016, 16, 1175–1187. 10.1002/mabi.201600071. 27106866

[ref11] Breunig M.; Lungwitz U.; Liebl R.; Goepferich A. Breaking up the correlation between efficacy and toxicity for nonviral gene delivery. Proc. Natl. Acad. Sci. U. S. A. 2007, 104, 14454–14459. 10.1073/pnas.0703882104. 17726101 PMC1964826

[ref12] Karimov M.; Appelhans D.; Ewe A.; Aigner A. The combined disulfide cross-linking and tyrosine-modification of very low molecular weight linear PEI synergistically enhances transfection efficacies and improves biocompatibility. Eur. J. Pharm. Biopharm 2021, 161, 56–65. 10.1016/j.ejpb.2021.02.005. 33582186

[ref13] Lin Y.; Wilk U.; Pohmerer J.; Horterer E.; Hohn M.; Luo X.; Mai H.; Wagner E.; Lachelt U. Folate Receptor-Mediated Delivery of Cas9 RNP for Enhanced Immune Checkpoint Disruption in Cancer Cells. Small 2023, 19, e2205318 10.1002/smll.202205318. 36399647

[ref14] Rui Y.; Wilson D. R.; Tzeng S. Y.; Yamagata H. M.; Sudhakar D.; Conge M.; Berlinicke C. A.; Zack D. J.; Tuesca A.; Green J. J. High-throughput and high-content bioassay enables tuning of polyester nanoparticles for cellular uptake, endosomal escape, and systemic in vivo delivery of mRNA. Sci. Adv. 2022, 8, eabk2855 10.1126/sciadv.abk2855. 34985952 PMC8730632

[ref15] Kang S. H.; Cho M. J.; Kole R. Up-regulation of luciferase gene expression with antisense oligonucleotides: implications and applications in functional assay development. Biochemistry 1998, 37, 6235–6239. 10.1021/bi980300h. 9572837

[ref16] Geyer A.; Lorenzer C.; Gehrig S.; Simlinger M.; Winkler J.; Sami H.; Ogris M. Fluorescence- and computed tomography for assessing the biodistribution of siRNA after intratracheal application in mice. Int. J. Pharm. 2017, 525, 359–366. 10.1016/j.ijpharm.2017.02.025. 28213277

[ref17] El-Andaloussi S.; Johansson H. J.; Lundberg P.; Langel U. Induction of splice correction by cell-penetrating peptide nucleic acids. J. Gene Med. 2006, 8, 1262–1273. 10.1002/jgm.950. 16900561

[ref18] Bauman J. A.; Li S. D.; Yang A.; Huang L.; Kole R. Anti-tumor activity of splice-switching oligonucleotides. Nucleic Acids Res. 2010, 38, 8348–8356. 10.1093/nar/gkq731. 20719743 PMC3001088

[ref19] Ezzat K.; Andaloussi S. E.; Zaghloul E. M.; Lehto T.; Lindberg S.; Moreno P. M.; Viola J. R.; Magdy T.; Abdo R.; Guterstam P.; Sillard R.; Hammond S. M.; Wood M. J.; Arzumanov A. A.; Gait M. J.; Smith C. I.; Hallbrink M.; Langel U. PepFect 14, a novel cell-penetrating peptide for oligonucleotide delivery in solution and as solid formulation. Nucleic Acids Res. 2011, 39, 5284–5298. 10.1093/nar/gkr072. 21345932 PMC3130259

[ref20] Saher O.; Lehto T.; Gissberg O.; Gupta D.; Gustafsson O.; Andaloussi S.E.; Darbre T.; Lundin K.E.; Smith C. I. E.; Zain R. Sugar and Polymer Excipients Enhance Uptake and Splice-Switching Activity of Peptide-Dendrimer/Lipid/Oligonucleotide Formulations. Pharmaceutics 2019, 11, 666 10.3390/pharmaceutics11120666. 31835435 PMC6955847

[ref21] Zaghloul E. M.; Viola J. R.; Zuber G.; Smith C. I.; Lundin K. E. Formulation and delivery of splice-correction antisense oligonucleotides by amino acid modified polyethylenimine. Mol. Pharmaceutics 2010, 7, 652–663. 10.1021/mp900220p. 20128628

[ref22] Wang Y.; Ma B.; Abdeen A. A.; Chen G.; Xie R.; Saha K.; Gong S. Versatile Redox-Responsive Polyplexes for the Delivery of Plasmid DNA, Messenger RNA, and CRISPR-Cas9 Genome-Editing Machinery. ACS Appl. Mater. Interfaces 2018, 10, 31915–31927. 10.1021/acsami.8b09642. 30222305 PMC6530788

[ref23] Dirisala A.; Uchida S.; Tockary T. A.; Yoshinaga N.; Li J.; Osawa S.; Gorantla L.; Fukushima S.; Osada K.; Kataoka K. Precise tuning of disulphide crosslinking in mRNA polyplex micelles for optimizing extracellular and intracellular nuclease tolerability. J. Drug Target 2019, 27, 670–680. 10.1080/1061186X.2018.1550646. 30499743

[ref24] Frohlich T.; Edinger D.; Russ V.; Wagner E. Stabilization of polyplexes via polymer crosslinking for efficient siRNA delivery. Eur. J. Pharm. Sci. 2012, 47, 914–920. 10.1016/j.ejps.2012.09.006. 23000380

[ref25] Chiper M.; Tounsi N.; Kole R.; Kichler A.; Zuber G. Self-aggregating 1.8 kDa polyethylenimines with dissolution switch at endosomal acidic pH are delivery carriers for plasmid DNA, mRNA, siRNA and exon-skipping oligonucleotides. J. Controlled Release 2017, 246, 60–70. 10.1016/j.jconrel.2016.12.005. 27956144

[ref26] Crooke S. T.; Wang S.; Vickers T. A.; Shen W.; Liang X. H. Cellular uptake and trafficking of antisense oligonucleotides. Nat. Biotechnol. 2017, 35, 230–237. 10.1038/nbt.3779. 28244996

[ref27] Kopatz I.; Remy J. S.; Behr J. P. A model for non-viral gene delivery: through syndecan adhesion molecules and powered by actin. J. Gene Med. 2004, 6, 769–776. 10.1002/jgm.558. 15241784

[ref28] Liang X. H.; Nichols J. G.; De Hoyos C. L.; Sun H.; Zhang L.; Crooke S. T. Golgi-58K can re-localize to late endosomes upon cellular uptake of PS-ASOs and facilitates endosomal release of ASOs. Nucleic Acids Res. 2021, 49, 8277–8293. 10.1093/nar/gkab599. 34244781 PMC8373082

[ref29] Miller C. M.; Wan W. B.; Seth P. P.; Harris E. N. Endosomal Escape of Antisense Oligonucleotides Internalized by Stabilin Receptors Is Regulated by Rab5C and EEA1 During Endosomal Maturation. Nucleic Acid Ther 2018, 28, 86–96. 10.1089/nat.2017.0694. 29437530 PMC5899299

[ref30] de Bruin K. G.; Fella C.; Ogris M.; Wagner E.; Ruthardt N.; Brauchle C. Dynamics of photoinduced endosomal release of polyplexes. J. Controlled Release 2008, 130, 175–182. 10.1016/j.jconrel.2008.06.001. 18585413

[ref31] van der Bent M. L.; da Silva Filho O. Paulino; Willemse M.; Hallbrink M.; Wansink D. G.; Brock R. The nuclear concentration required for antisense oligonucleotide activity in myotonic dystrophy cells. FASEB J. 2019, 33, 11314–11325. 10.1096/fj.201900263R. 31311315

[ref32] Deprey K.; Batistatou N.; Debets M. F.; Godfrey J.; VanderWall K. B.; Miles R. R.; Shehaj L.; Guo J.; Andreucci A.; Kandasamy P.; Lu G.; Shimizu M.; Vargeese C.; Kritzer J. A. Quantitative Measurement of Cytosolic and Nuclear Penetration of Oligonucleotide Therapeutics. ACS Chem. Biol. 2022, 17, 348–360. 10.1021/acschembio.1c00830. 35034446 PMC9252293

[ref33] Rocha C. S.; Lundin K. E.; Behlke M. A.; Zain R.; El Andaloussi S.; Smith C. I. Four Novel Splice-Switch Reporter Cell Lines: Distinct Impact of Oligonucleotide Chemistry and Delivery Vector on Biological Activity. Nucleic Acid Ther 2016, 26, 381–391. 10.1089/nat.2016.0631. 27629437

[ref34] Hammond S. M.; McClorey G.; Nordin J. Z.; Godfrey C.; Stenler S.; Lennox K. A.; Smith C. I.; Jacobi A. M.; Varela M. A.; Lee Y.; Behlke M. A.; Wood M. J.; Andaloussi S. E. Correlating In Vitro Splice Switching Activity With Systemic In Vivo Delivery Using Novel ZEN-modified Oligonucleotides. Mol. Ther Nucleic Acids 2014, 3, e212 10.1038/mtna.2014.63. 25423116 PMC4459549

[ref35] Ivics Z.; Mates L.; Yau T. Y.; Landa V.; Zidek V.; Bashir S.; Hoffmann O. I.; Hiripi L.; Garrels W.; Kues W. A.; Bosze Z.; Geurts A.; Pravenec M.; Rulicke T.; Izsvak Z. Germline transgenesis in rodents by pronuclear microinjection of Sleeping Beauty transposons. Nat. Protoc 2014, 9, 773–793. 10.1038/nprot.2014.008. 24625778

[ref36] Rulicke T. Pronuclear microinjection of mouse zygotes. Methods Mol. Biol. 2004, 254, 165–194. 10.1385/1-59259-741-6:165. 15041762

[ref37] Dean J. M.; DeLong R. K. A high-throughput screening assay for the functional delivery of splice-switching oligonucleotides in human melanoma cells. Methods Mol. Biol. 2015, 1297, 187–196. 10.1007/978-1-4939-2562-9_13. 25896004 PMC5558459

[ref38] Roberts J.; Palma E.; Sazani P.; Orum H.; Cho M.; Kole R. Efficient and persistent splice switching by systemically delivered LNA oligonucleotides in mice. Mol. Ther 2006, 14, 471–475. 10.1016/j.ymthe.2006.05.017. 16854630

[ref39] Dang Y.; van Heusden C.; Nickerson V.; Chung F.; Wang Y.; Quinney N. L.; Gentzsch M.; Randell S. H.; Moulton H. M.; Kole R.; Ni A.; Juliano R. L.; Kreda S. M. Enhanced delivery of peptide-morpholino oligonucleotides with a small molecule to correct splicing defects in the lung. Nucleic Acids Res. 2021, 49, 6100–6113. 10.1093/nar/gkab488. 34107015 PMC8216463

[ref40] Juliano R. L.; Ming X.; Carver K.; Laing B. Cellular uptake and intracellular trafficking of oligonucleotides: implications for oligonucleotide pharmacology. Nucleic Acid Ther 2014, 24, 101–113. 10.1089/nat.2013.0463. 24383421 PMC3962645

[ref41] van de Steeg E.; Lappchen T.; Aguilera B.; Jansen H. T.; Muilwijk D.; Vermue R.; van der Hoorn J. W.; Donato K.; Rossin R.; de Visser P. C.; Vlaming M. L. H. Feasibility of SPECT-CT Imaging to Study the Pharmacokinetics of Antisense Oligonucleotides in a Mouse Model of Duchenne Muscular Dystrophy. Nucleic Acid Ther 2017, 27, 221–231. 10.1089/nat.2016.0649. 28418733

[ref42] Crooke R. M.; Graham M. J.; Martin M. J.; Lemonidis K. M.; Wyrzykiewiecz T.; Cummins L. L. Metabolism of antisense oligonucleotides in rat liver homogenates. J. Pharmacol Exp Ther 2000, 292, 140–149. 10.1016/S0022-3565(24)35270-X. 10604941

[ref43] He C.; Migawa M. T.; Chen K.; Weston T. A.; Tanowitz M.; Song W.; Guagliardo P.; Iyer K. S.; Bennett C. F.; Fong L. G.; Seth P. P.; Young S. G.; Jiang H. High-resolution visualization and quantification of nucleic acid-based therapeutics in cells and tissues using Nanoscale secondary ion mass spectrometry (NanoSIMS). Nucleic Acids Res. 2021, 49, 1–14. 10.1093/nar/gkaa1112. 33275144 PMC7797060

[ref44] Groza D.; Gehrig S.; Kudela P.; Holcmann M.; Pirker C.; Dinhof C.; Schueffl H. H.; Sramko M.; Hoebart J.; Alioglu F.; Grusch M.; Ogris M.; Lubitz W.; Keppler B. K.; Pashkunova-Martic I.; Kowol C. R.; Sibilia M.; Berger W.; Heffeter P. Bacterial ghosts as adjuvant to oxaliplatin chemotherapy in colorectal carcinomatosis. Oncoimmunology 2018, 7, e1424676 10.1080/2162402X.2018.1424676. 29721389 PMC5927527

[ref45] Zaslavsky A.; Chen C.; Grillo J.; Baek K. H.; Holmgren L.; Yoon S. S.; Folkman J.; Ryeom S. Regional control of tumor growth. Mol. Cancer Res. 2010, 8, 1198–1206. 10.1158/1541-7786.MCR-10-0047. 20736295 PMC3044487

[ref46] Smrekar B.; Wightman L.; Wolschek M. F.; Lichtenberger C.; Ruzicka R.; Ogris M.; Rodl W.; Kursa M.; Wagner E.; Kircheis R. Tissue-dependent factors affect gene delivery to tumors in vivo. Gene Ther. 2003, 10, 1079–1088. 10.1038/sj.gt.3301965. 12808438

[ref47] Boeckle S.; von Gersdorff K.; van der Piepen S.; Culmsee C.; Wagner E.; Ogris M. Purification of polyethylenimine polyplexes highlights the role of free polycations in gene transfer. J. Gene Med. 2004, 6, 1102–1111. 10.1002/jgm.598. 15386739

[ref48] Bozoglu T.; Lee S.; Ziegler T.; Jurisch V.; Maas S.; Baehr A.; Hinkel R.; Hoenig A.; Hariharan A.; Kim C. I.; Decker S.; Sami H.; Koppara T.; Oellinger R.; Muller O. J.; Frank D.; Megens R.; Nelson P.; Weber C.; Schnieke A.; Sperandio M.; Santamaria G.; Rad R.; Moretti A.; Laugwitz K. L.; Soehnlein O.; Ogris M.; Kupatt C. Endothelial Retargeting of AAV9 In Vivo. Adv. Sci. 2022, 9, e2103867 10.1002/advs.202103867. PMC8895123 35023328

[ref49] Taschauer A.; Polzer W.; Alioglu F.; Billerhart M.; Decker S.; Kittelmann T.; Geppl E.; Elmenofi S.; Zehl M.; Urban E.; Sami H.; Ogris M. Peptide-Targeted Polyplexes for Aerosol-Mediated Gene Delivery to CD49f-Overexpressing Tumor Lesions in Lung. Mol. Ther Nucleic Acids 2019, 18, 774–786. 10.1016/j.omtn.2019.10.009. 31734558 PMC6861568

[ref50] Vetter V. C.; Wagner E. Targeting nucleic acid-based therapeutics to tumors: Challenges and strategies for polyplexes. J. Controlled Release 2022, 346, 110–135. 10.1016/j.jconrel.2022.04.013. 35436520

[ref51] Decker S.; Taschauer A.; Geppl E.; Pirhofer V.; Schauer M.; Poschl S.; Kopp F.; Richter L.; Ecker G. F.; Sami H.; Ogris M. Structure-based peptide ligand design for improved epidermal growth factor receptor targeted gene delivery. Eur. J. Pharm. Biopharm 2022, 176, 211–221. 10.1016/j.ejpb.2022.05.004. 35584718

[ref52] Casper J.; Schenk S. H.; Parhizkar E.; Detampel P.; Dehshahri A.; Huwyler J. Polyethylenimine (PEI) in gene therapy: Current status and clinical applications. J. Controlled Release 2023, 362, 667–691. 10.1016/j.jconrel.2023.09.001. 37666302

[ref53] Lecocq M.; Wattiaux-De Coninck S.; Laurent N.; Wattiaux R.; Jadot M. Uptake and intracellular fate of polyethylenimine in vivo. Biochem. Biophys. Res. Commun. 2000, 278, 414–418. 10.1006/bbrc.2000.3809. 11097851

[ref54] Bonnal S. C.; Lopez-Oreja I.; Valcarcel J. Roles and mechanisms of alternative splicing in cancer - implications for care. Nat. Rev. Clin Oncol 2020, 17, 457–474. 10.1038/s41571-020-0350-x. 32303702

[ref55] Godfrey C.; Desviat L. R.; Smedsrod B.; Pietri-Rouxel F.; Denti M. A.; Disterer P.; Lorain S.; Nogales-Gadea G.; Sardone V.; Anwar R.; El Andaloussi S.; Lehto T.; Khoo B.; Brolin C.; van Roon-Mom W. M.; Goyenvalle A.; Aartsma-Rus A.; Arechavala-Gomeza V. Delivery is key: lessons learnt from developing splice-switching antisense therapies. EMBO Mol. Med. 2017, 9, 545–557. 10.15252/emmm.201607199. 28289078 PMC5412803

[ref56] Prabst K.; Engelhardt H.; Ringgeler S.; Hubner H. Basic Colorimetric Proliferation Assays: MTT, WST, and Resazurin. Methods Mol. Biol. 2017, 1601, 1–17. 10.1007/978-1-4939-6960-9_1. 28470513

