# Supplementary material for: Accelerated Endosomal Escape of Splice-Switching Oligonucleotides Enables Efficient Hepatic Splice Correction
Source: ACS Appl Mater Interfaces. 2025 Jan 28;17(6):9000–18. doi: 10.1021/acsami.4c19340 (PMC11826512; doi:10.1021/acsami.4c19340)
Supplement: Supplementary file 1 — am4c19340_si_001.pdf [file am4c19340_si_001.pdf]

Supporting Information

Accelerated endosomal escape of splice-switching oligonucleotides enables efficient hepatic splice correction

*Silvia Weiss [a #], Simon Decker [a #], Christoph Kugler [a], Laura Bocanegra Gómez [a], Helene Fasching [a], Denise Benisch [a], Fatih Alioglu [a], Levente Ferencz [a], Theresa Birkfeld [a], Filip Ilievski [a $], Volker Baumann [a], Alina Duran [a], Enes Dusinovic [a], Nadine Follrich [a], Sandra Milenkovic [a], Dajana Mihalicokova [a], Daniel Paunov [a], Karla Singeorzan [a], Nikolaus Zehetmayer [a], Dejan Zivanonvic [a], Ulrich Lächelt [a], Auke Boersma [b], Thomas Rülicke [c], Haider Sami [a *], Manfred Ogris [a *]*

[a] University of Vienna, Faculty of Life Sciences, Department of Pharmaceutical Sciences, Laboratory of Macromolecular Cancer Therapeutics (MMCT), Josef-Holaubek-Platz 2, 1090 Vienna, Austria

[b] Institute of In-vivo and In-vitro Models, Biomodels Austria, Department of Biomedical Sciences, University of Veterinary Medicine Vienna, Veterinärplatz 1, A-1210 Vienna Austria

[c] Department of Biomedical Sciences and Pathobiology, University of Veterinary Medicine Vienna and Ludwig Boltzmann Institute for Hematology and Oncology, Veterinärplatz 1, A-1210 Vienna, Austria

*Correspondence should be addressed to:

Manfred Ogris
Phone: +43 1 4277 55551
Email: m.ogris@univie.ac.at

Haider Sami
Phone: +43 1 4277 55599

Email: haider.sami@univie.ac.at

[#]These authors contributed equally to this work

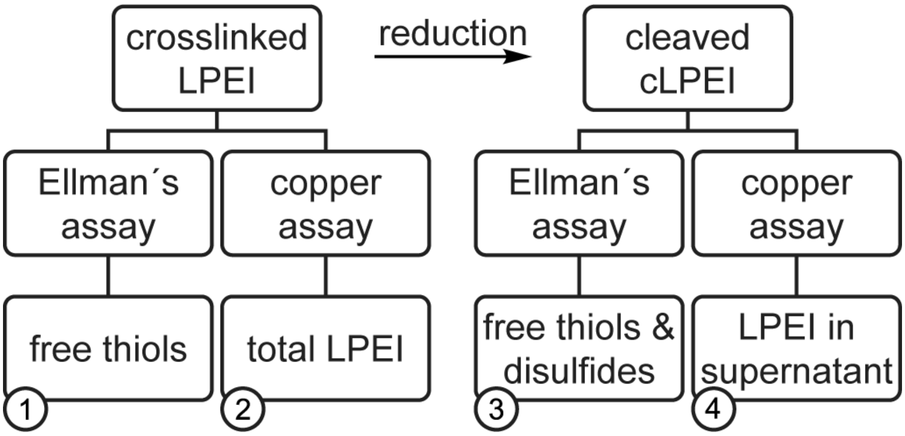

Figure S1. Schematic illustration of characterization of cross-linking of low molecular weight linear polyethylenimine by homo-bifunctional dithiobis(succinimidyl propionate) (Lomant's reagent) and estimation of cross-linking ratio. After reductive cleavage of disulfide bridges, thiols are quantified in supernatant (3). This value is corrected for free thiols present before reduction (1). Due to significant sample loss during reduction (sample bound to resin) (1) is multiplied by the ratio of sample present before reduction (2) to sample in supernatant afterwards (4). Finally, (1) corrected to sample loss (4 ÷ 2), is subtracted from (3). Molar ratio of disulfides to LPEI in supernatant is expressed as % crosslinked, which was estimated at 8.3 % i.e. one out of twelve LPEI molecules is crosslinked.

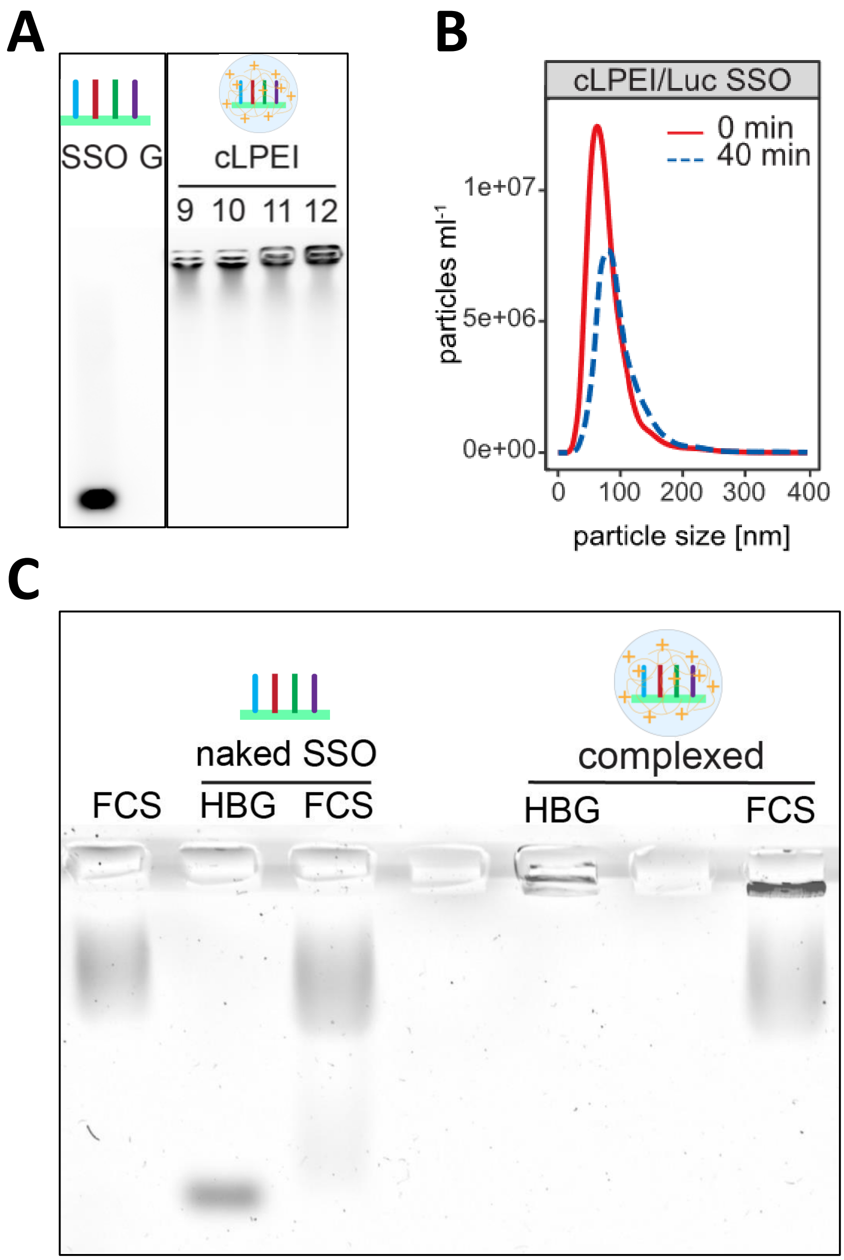

Figure S2. Biophysical testing. (**A**) Gel retardation of AF647 labelled SSO after complexation using cLPEI at indicated N/P ratios (9, 10, 11, 12) on a 1.5 % agarose gel. Naked SSO and glycerol (G) were used as controls. (**B**) NTA based stability of of cLPEI-SSO polyplexes generated in HBG buffer for in vivo application (SSO concentration of 400 µg ml-1, cLPEI-SSO-Luc, N/P 9) and measured immediately or after 40 min incubation at 25° C. Representative particle size to concentration profile are shown for both time points. (**C**) Stability of cLPEI-complexed SSO (N/P 9) against serum: Gel retardation of naked versus cLPEI-complexed SSO after incubation with HBG buffer or serum (FCS) for four hours and subsequent running on a 1.5 % agarose gel. 400 ng SSO were administered per well; pure FCS was used as a control.

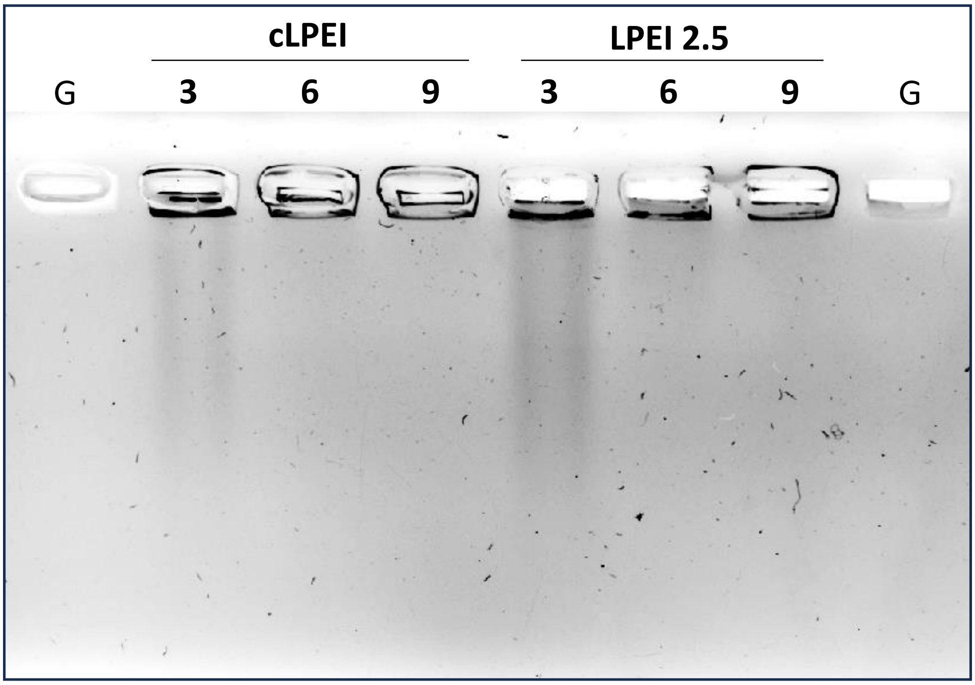

Figure S3. Comparison of SSO complexation by cLPEI and LPEI 2.5. Gel retardation of NC SSO after complexation with cLPEI or LPEI 2.5 at indicated N/P ratios of 3, 6 and 9 on a 0.75 % agarose gel (stained with EtBr). 400 ng SSO were loaded per well. Glycerol (G) was used as loading control. Representative gel shown from two experiments.

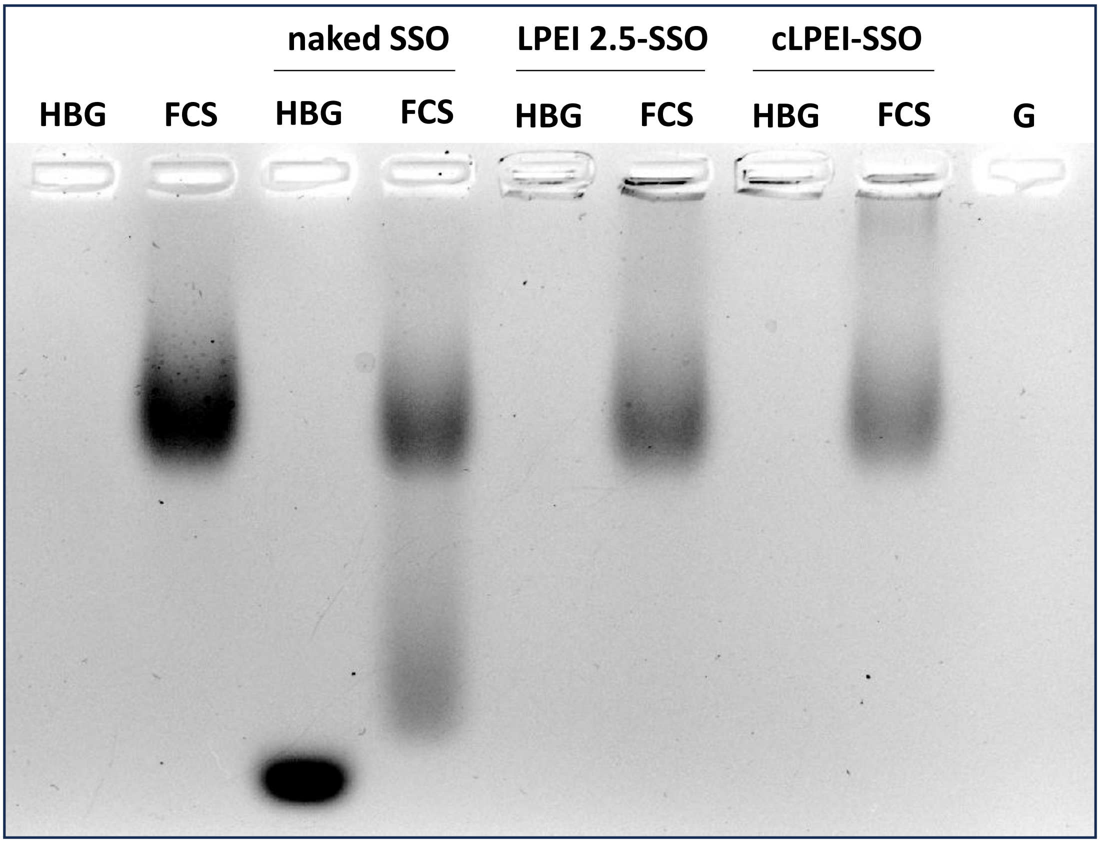

Figure S4. Comparison of serum stability between cLPEI and LPEI 2.5 polyplexes. Gel retardation of LPEI 2.5-complexed SSOs versus cLPEI-complexed SSOs (both at N/P 9) after incubation with HBG buffer (HBG) or serum (FCS) for 30 minutes and subsequent running on a 0.75 % agarose gel (stained with EtBr). 400 ng SSO were loaded per well. HBG buffer (HBG), pure serum (FCS) and glycerol (G) were used as controls. Representative gel shown from two experiments.

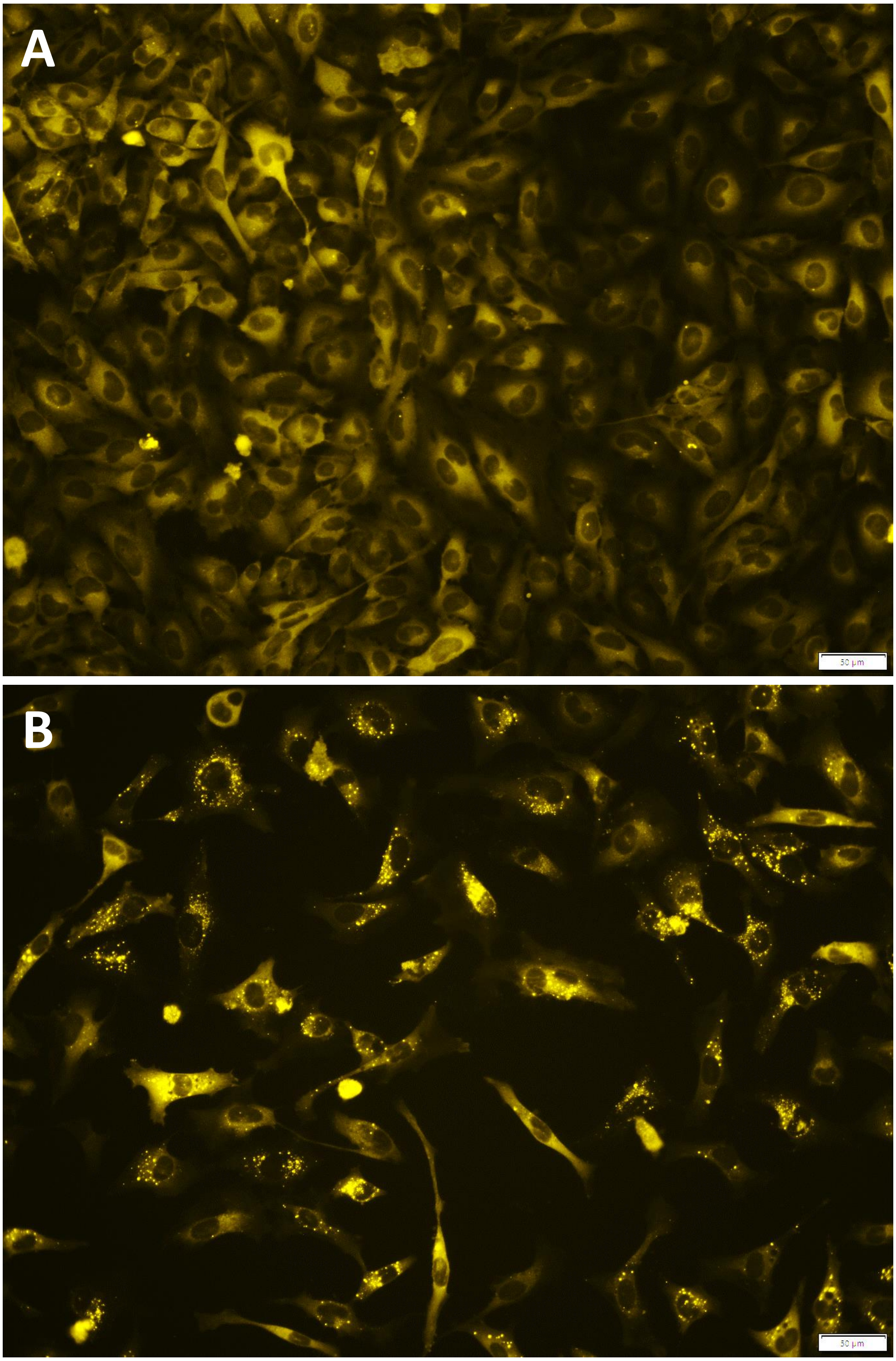

Figure S5. Positive control treatment optimization for endosomal escape assay using Hela mRuby-3 galectin 8 cells. (**A**) Untreated cells with a homogenous cytoplasm, which is due to dispersed Gal8, are shown. The bright spots depict dead cells. (**B**) Cells were treated with 40 µM chloroquine for 24 hours. Endosomal escape is clearly visible by the clusters of Gal8 within the cytoplasm of cells. Both images were acquired using 1 s of exposure and a 20x objective; scale bar: 50 µm, n = 8.

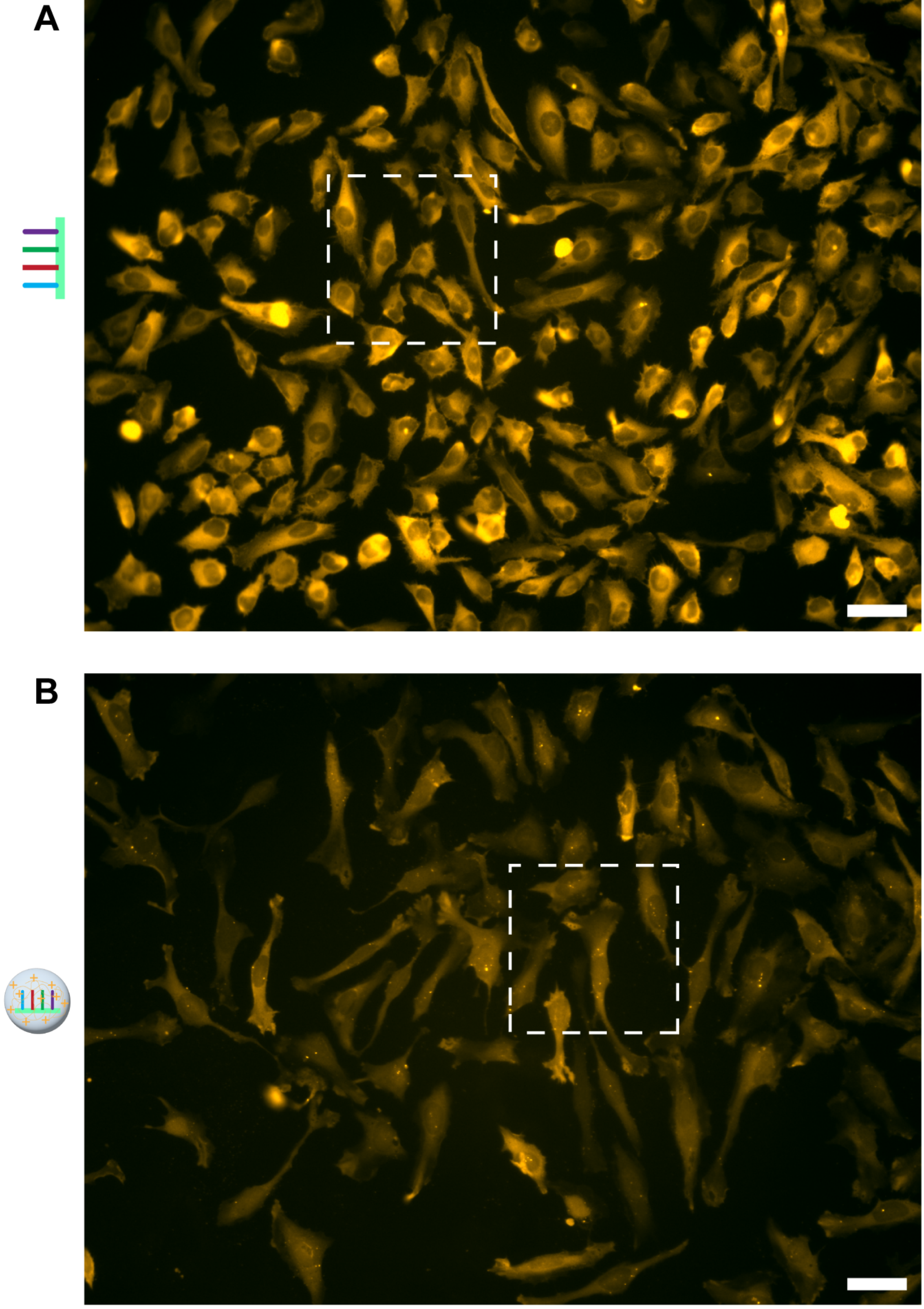

Figure S6. Endosomal escape using Hela mRuby-3 galectin 8 cells after 4 hours. (**A**) Cells treated with 160 pmol naked SSO-NC are shown. Individual scattered clusters of Gal8 are visible. (**B**) cLPEI-SSO-NC treated cells with accumulations of Gal8-clusters. Both images were acquired using 2 s of exposure time and a 20x objective; scale bar: 50 µm, n = 18. The white squares depict the magnifications shown in Fig 3.

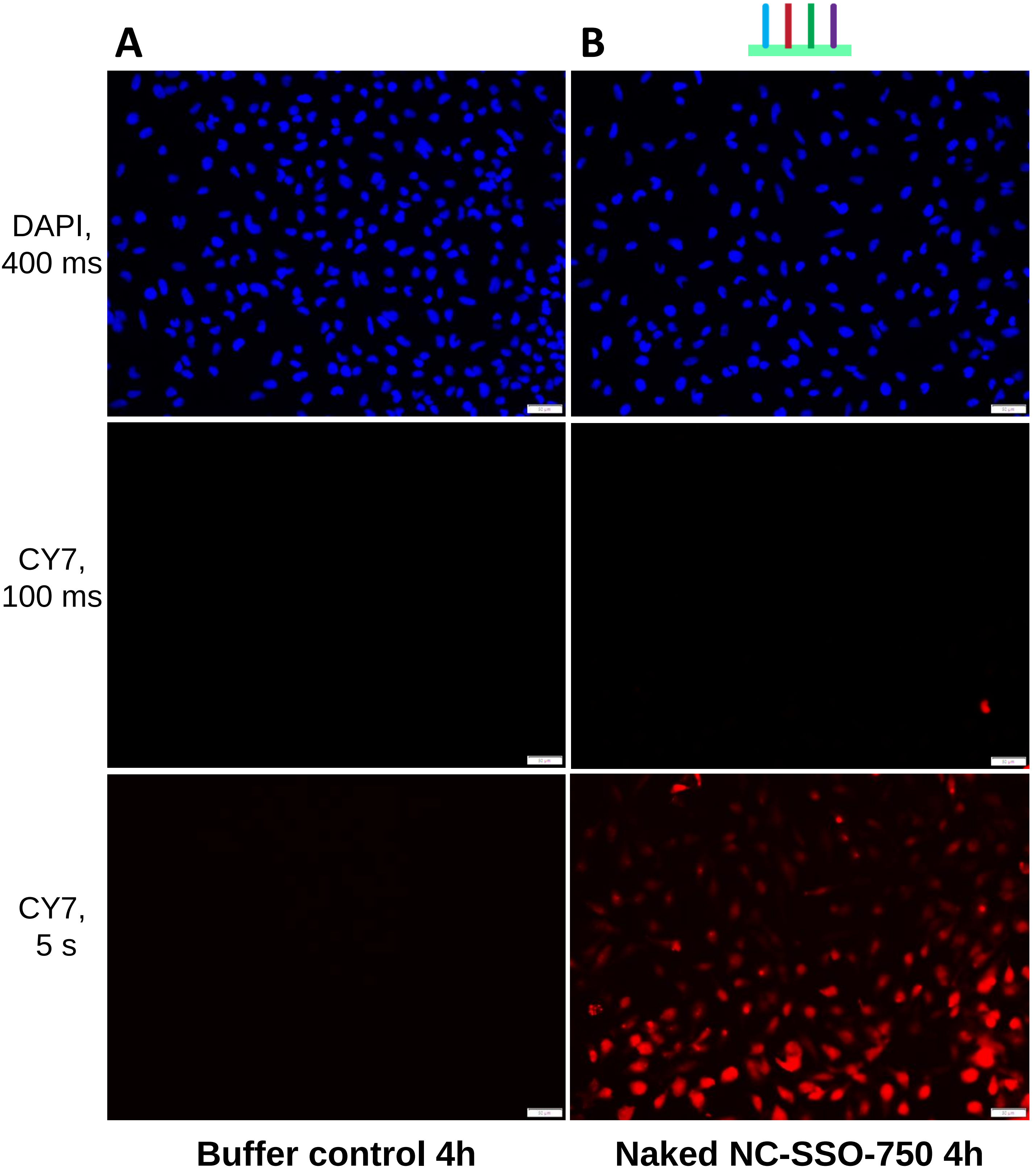

Figure S7. Nuclear entry using Hela mRuby-3 galectin 8 cells. (**A**) Cells treated with buffer show no nuclear signal within the Cy7 filter irrespective of the exposure time. (**B**) Cells treated with 160 pmol naked SSO-NC-AF750 for four hours and nuclei co-stained with DAPI. These cells show barely any signal for 100 ms of exposure. However, when increasing it to the maximum of 5 s, a clear nuclear signal can be observed in all cells. Images were acquired using a 20x objective; scale bar: 50 µm, n = 18; Cy7 filter set was used to image AF750 signal.

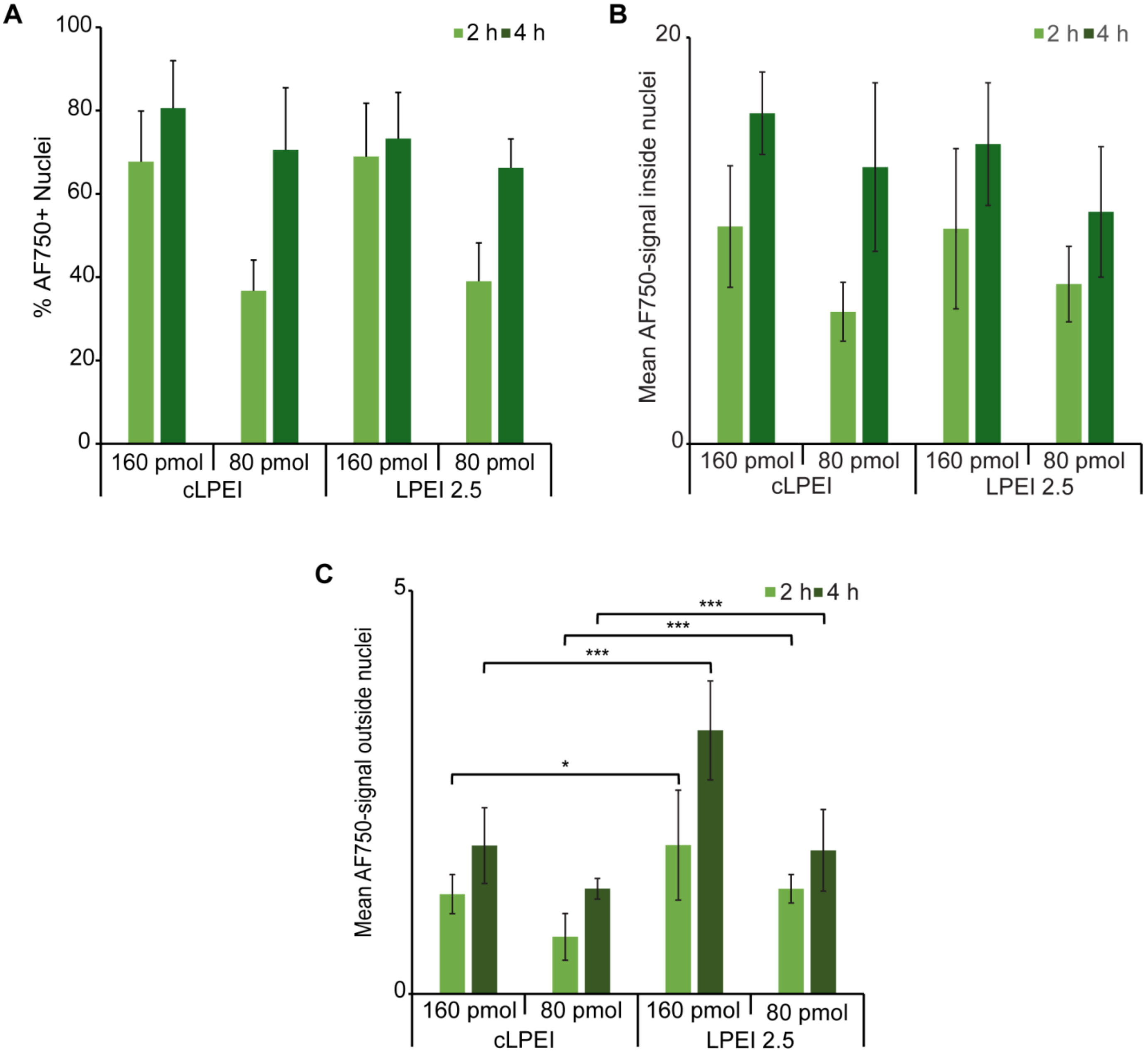

Figure S8. Image based quantification of nuclear delivery of SSOs by (A) percentage of AF750 positive nuclei, (B) AF750-signal intensity inside, and (C) AF750-signal intensity outside nuclei after treatment with SSO-polyplexes. Hela mRuby-3 galectin 8 cells were treated with either 80 or 160 pmol complexed SSO-NC-AF750 (N/P 9). For complexation cLPEI or LPEI 2.5 was used, and cells were incubated for two or four hours. Nuclei were stained using DAPI. Imaging and analysis were performed as described. n = 18 for cLPEI, n = 12 for LPEI 2.5. Vales are not statically different using a Welch's T-test (two-tailed, $p > 0.05$) for A and B, hower * $p < 0.05$, and *** $p < 0.001$ in C, data shown as mean ±SD.

cLPEI 4h
SSO-750,
100ms

LPEI 2.5 4h
SSO-750,
100ms

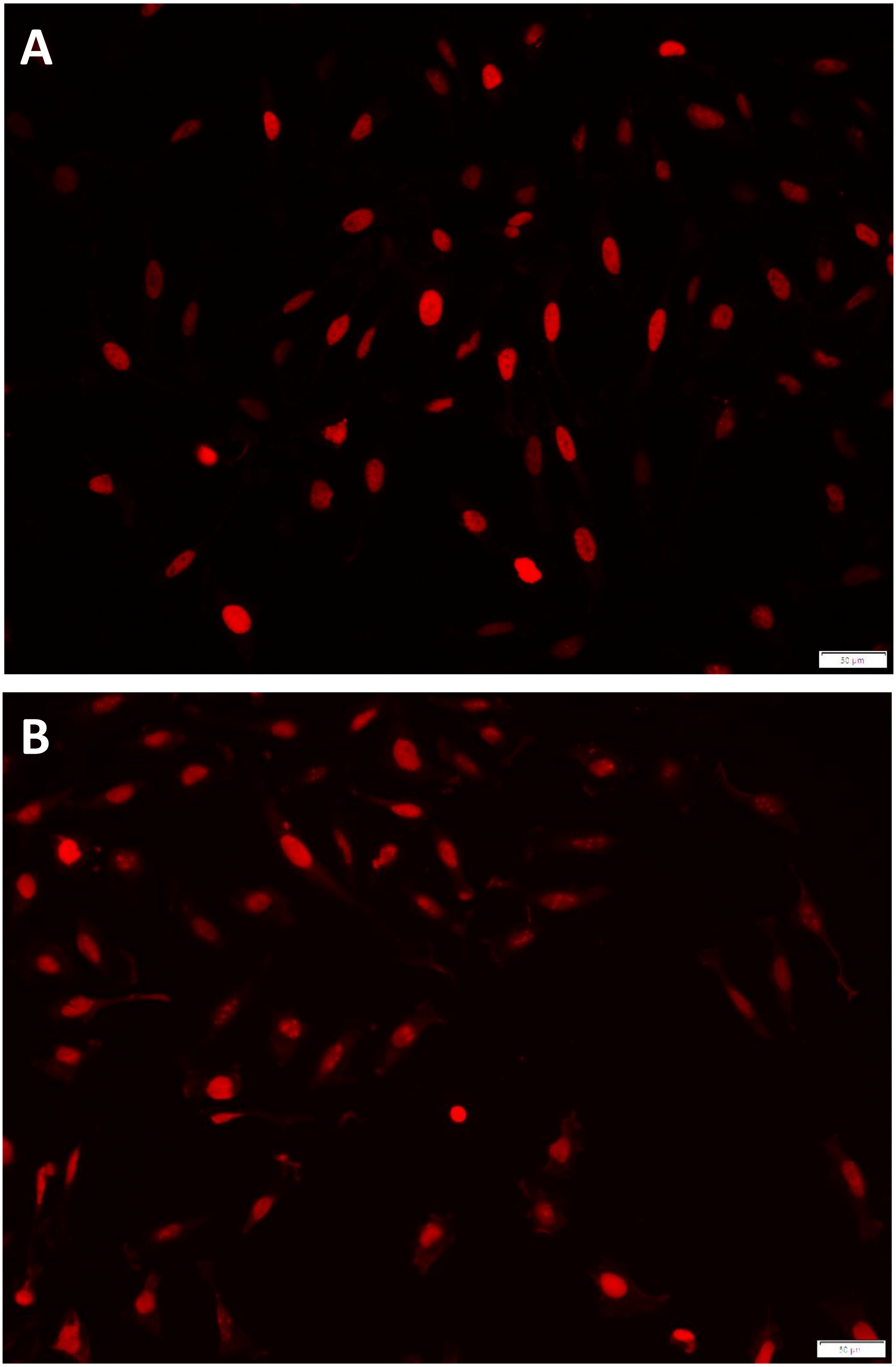

Figure S9. FLM-images of Hela-Gal8 cells after four hours of incubation with 160 pmol of AF750-labelled SSOs as (**A**) cLPEI-SSO-NC-750 N/P 9 polyplex or (B) LPEI-SSO-NC-750 N/P 9 polyplex for imaging intracellular localization of SSOs. Images were acquired using 100 ms of exposure time and a 20x objective; scale bar: 50 µm, n = 18, Cy7 filter set was used to image AF750 signal.

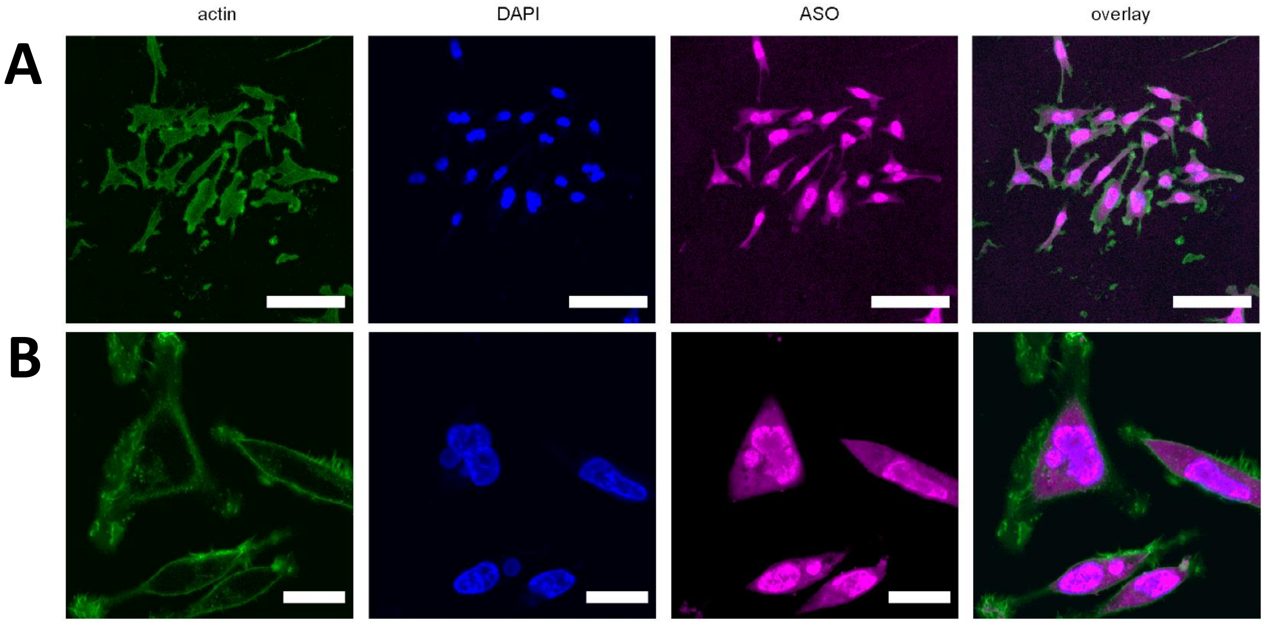

Figure S10. Intracellular SSO delivery to target organelle via cLPEI polyplexes. HeLa pLuc 705 cells were treated with 160 pmol of cLPEI-SSO-Luc-647 (N/P 9, magenta) for 24 hours. Cells were then stained with phalloidin (green) and DAPI (blue). (A) Confocal laser scanning microscopy (CLSM) maximum intensity projection of z-stacks acquired using a 20x objective; scale bar: 100 µm. (B) CLSM middle slice of z-stacks acquired using a 63xOil objective. The same field of view as in Figure 4D is shown; scale bar: 25 µm.

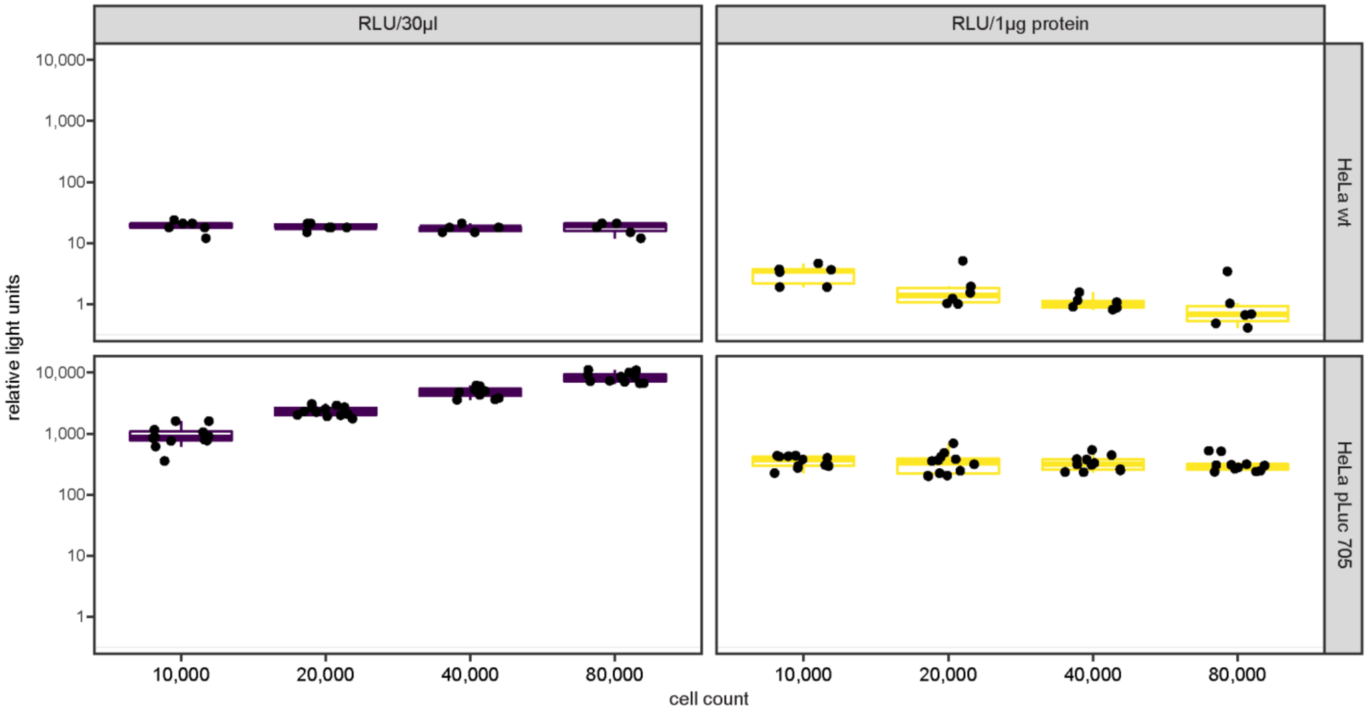

Figure S11. Characterization of the basal level of luminescence in HeLa pLuc 705 cell line used for in vitro splice correction assay. Cells were seeded in increasing cell counts and expression of luciferase measured 24 h thereafter. Results shown are from 4 independent experiments measured in triplicates, data shown as mean ±SD.

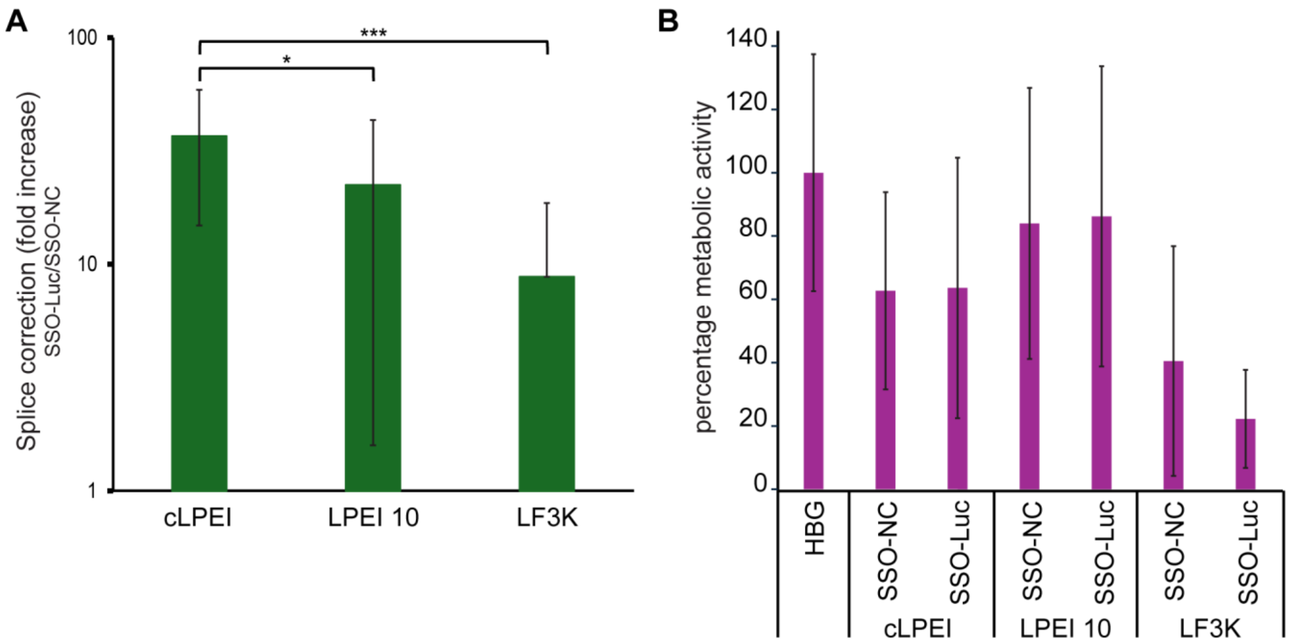

Figure S12. Comparison of cLPEI polyplexes with LPEI 10 and lipofectamine 3000 based formulations for transfection ability and biocompatibility profile. (A) Presented data is splice correction shown in figure 6B but here calculated as fold increase by dividing the luminescence from Luc-SSO treatments by that of NC-SSO treatments. Luminescence was measured from HeLa pLuc 705 cells 24 h after treatment with different SSO polyplexes. Dose of SSO, either NC SSO or Luc SSO, was kept constat at 160 pmol while different complexing agents were used: cLPEI, LPEI 10 and lipofectamine 3000 (LF3K), n = 12. (B) Resazurin assay based cell viability showing percentage metabolic activity of HeLa pLuc 705 cells 24 h after treatment with different SSO polyplexes indicated and normalized to HBG ctrl. Statistics were calculated using a Welch's T-test (two-tailed, *: $p < 0.05$, ***: $p < 0.001$), data shown as mean ±SD.

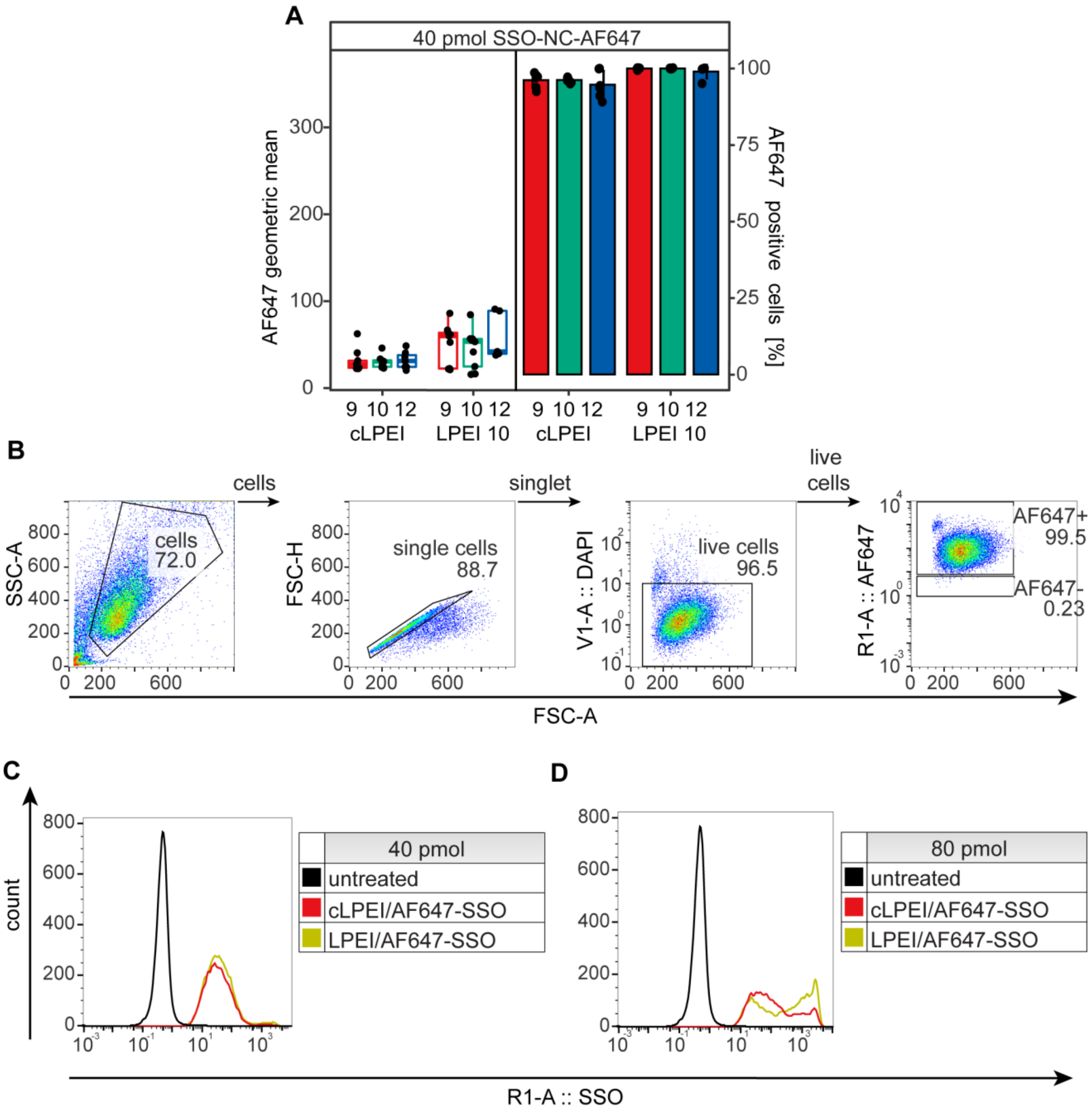

Figure S13. Cell association and uptake efficiency of cLPEI and LPEI 10 based SSO polyplexes measured by flow cytometry. (A) Cellular association of SSO-NC-647 polyplexes with HeLa pLuc 705 cells 24 h after treatment at 40 pmol SSO-NC-647. Both cLPEI and LPEI 10 were used at different N/P ratios (N/P 9, 10, and 12) and geometric mean of AF647 (left) as well as percentage of AF647 positive cells (right) are shown. (B) Gating strategy used for processing the flow cytometry data presented in Fig. 6D and S13A. Numbers indicate percentage of gated cells. (C) Representative histograms of data shown in Fig. S13A. (D) Representative histograms of data shown in Fig. 6D. n = 5, data shown as mean ±SD.

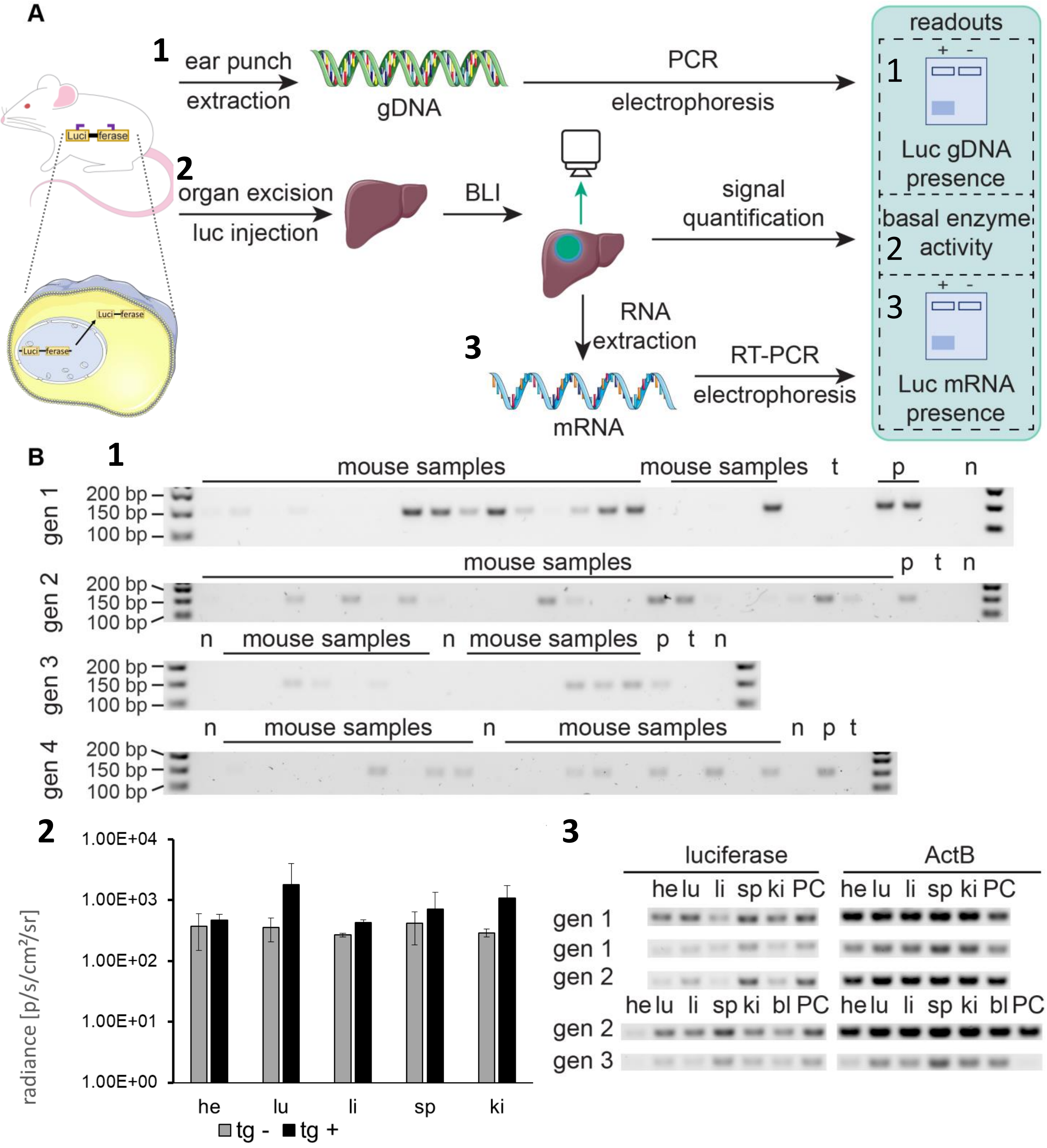
A
1
ear punch
extraction
gDNA
PCR
electrophoresis
readouts
1
+ -
Luc gDNA presence
2
organ excision
luc injection
BLI
signal quantification
basal enzyme activity
2
Luci ferase
3
RNA extraction
mRNA
RT-PCR
electrophoresis
3
+ -
Luc mRNA presence
B
1
gen 1
200 bp
150 bp
100 bp
mouse samples
mouse samples
t
p
n
gen 2
mouse samples
p t n
gen 3
n mouse samples n mouse samples p t n
gen 4
n mouse samples n mouse samples n p t
2
radiance [p/s/cm²/sr]
1.00E+04
1.00E+03
1.00E+02
1.00E+01
1.00E+00
he lu li sp ki
tg - tg +
3
luciferase
ActB
he lu li sp ki PC
gen 1
gen 1
gen 2
he lu li sp ki bl PC
gen 2
gen 3

Figure S14.

Figure S14. Evaluation of split-luciferase transgenic reporter mice for expression of split-luciferase gene, basal bioluminescence signals and split-luciferase mRNA within different organs. (A) Schematic overview of methods applied for the characterization of novel transgenic reporter mice for split-luciferase based splice correction. (A1) Ear punches were used for extraction of genomic DNA (gDNA) and PCR with subsequent gel electrophoreses performed. Luciferase sensitive primers (LSP) were used for the PCR, gels were accordingly analyzed for the presence of the bands specific for Luc gDNA. (B1) Representative gels of different generations of mice (gen 1-4) tested for the presence of Luc gDNA are shown. T: negative control template (wild type mouse), n: negative control (water instead of gDNA in mastermix), p: positive control (plasmid DNA). (A2) Animals of different generations were euthanized, organs excised after luciferin injection and ex vivo BLI performed to detect the baseline enzyme activity of each individual organ. (B2) Baseline bioluminescence signal of non-transgenic (tg -) versus transgenic (tg +) mice. No statistically significant difference was detectable (two-sided t-test, ($p > 0.05$), $n = 3$ for both tg - and tg +. (A3) RNA was extracted from organs measured in A2 and RT-PCR with subsequent gel electrophoresis performed. LSP as well as beta actin primer (ActB) were used for the PCR, gels were analyzed for the presence of both bands. (B3) Representative RT-PCR based gels from organs of different generations of mice (gen 1-3) are shown. He: heart, lu: lung, li: liver, sp: spleen, ki: kidney, bl: bladder.

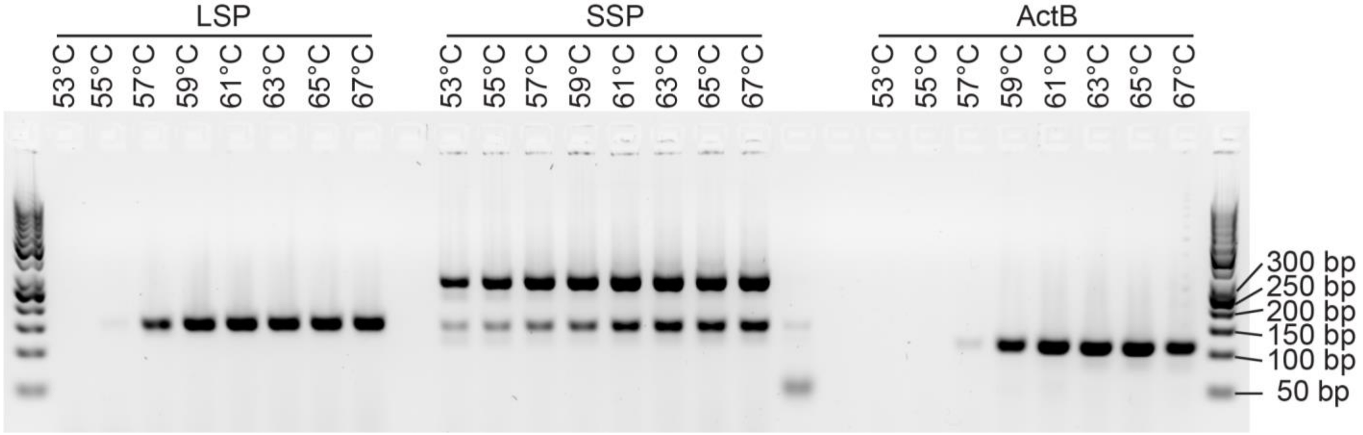

Figure S15. Establishment and optimization of the RT-PCR workflow for primer annealing temperatures. Three primer sets were designed and tested, namely, luciferase sensitive primer (LSP), splice sensitive primer (SSP), and beta Actin (ActB). HeLa pLuc 705 cells were transfected with commercial polyethylenimine based transfection Luc SSO polyplexes and whole RNA extracted after transfection by using RNeasy mini (Qiagen) kit according to manufacturer's protocol. The produced cDNA was then used for optimizing the annealing temperature for all three primers sets for 40 cycles at 53 – 67 °C annealing temperature, in 2°C increments, on the Thermal Cycler XT96 (VWR, UK).

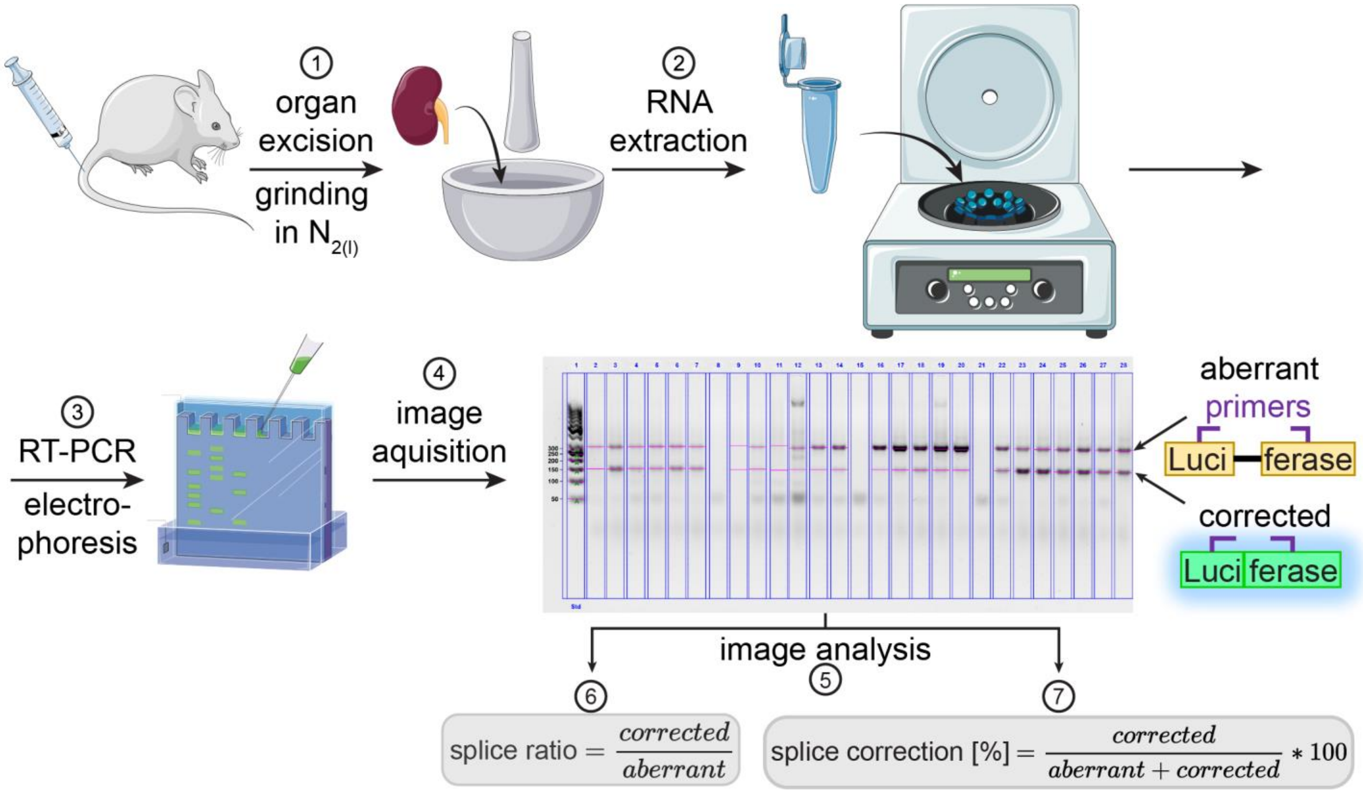

Figure S16. Workflow for calculating splice ratio and splice correction % in-vivo. (1) Mice were treated with SSO via tail vein injection for 48 h, then terminated, organs excised. and pulverized using mortar and pestle in liquid nitrogen ($N_{2(l)}$) . (2) Whole RNA was extracted from organ powder and purified on silica spin-columns. (3) mRNA was transcribed into cDNA and amplified using splice sensitive primers. (RT-PCR) Resulting amplicons were separated via agarose gel-electrophoresis and (4) whole gels imaged. (5) Gel images were analyzed (representative picture shown here) and bands integrated. The upper (aberrant) band corresponds to aberrantly spliced luciferase, the lower band (corrected) to correctly spliced luciferase. (6) Splice ratio and (7) splice correction were calculated as per shown formulae (grey boxes). The Figure was partly generated using Servier Medical Art, provided by Servier, licensed under a Creative Commons Attribution 3.0 unported license.

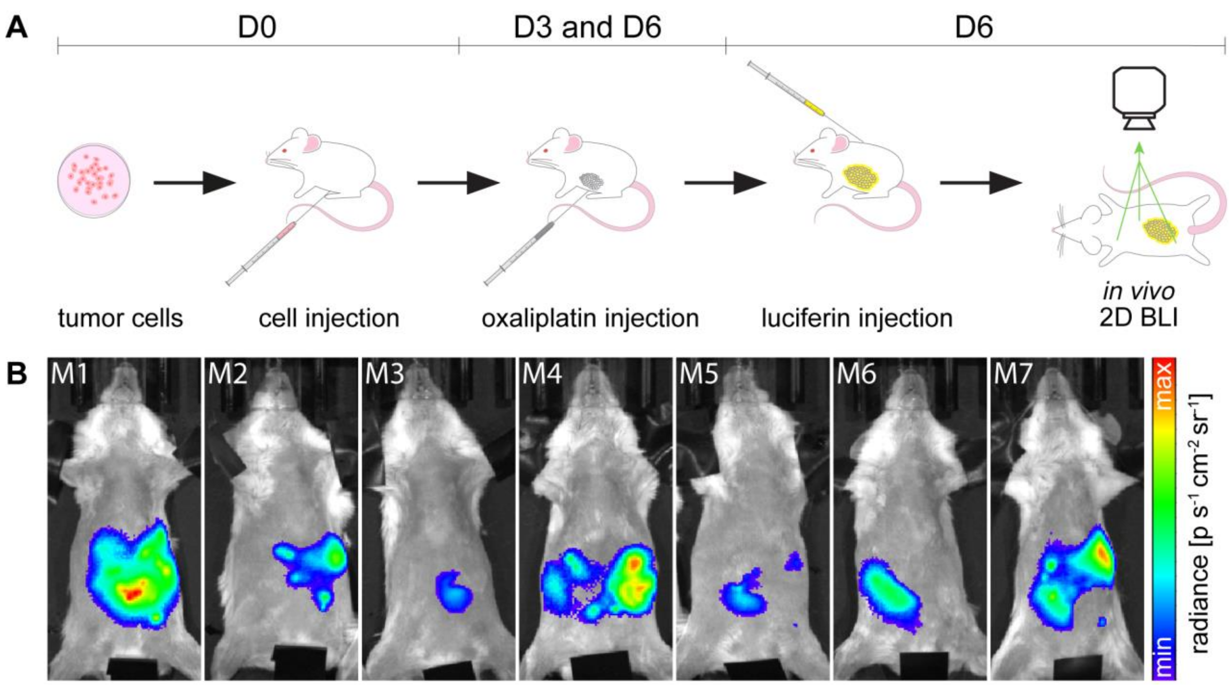

Figure S17. Tumor implantation, oxaliplatin treatment and bioluminescence imaging schedule. (A) 1 x 10^5 CT26-Luc tumor cells were injected i.p. into 9-week-old female Balb/c mice; on days 3 and 6 oxaliplatin (6 mg kg^{-1}, i.p.) was administered. On day 6, luciferin (30 mg kg^{-1}, s.c.) was injected additionally and 2D-BLI conducted. Thereby, (B) tumor growth was measured. Mx: mouse number. Mice M1-M7, bearing intraperitoneal CT26Luc colorectal tumors, were then used for SSO biodistribution study on day15, as described in figure S18.

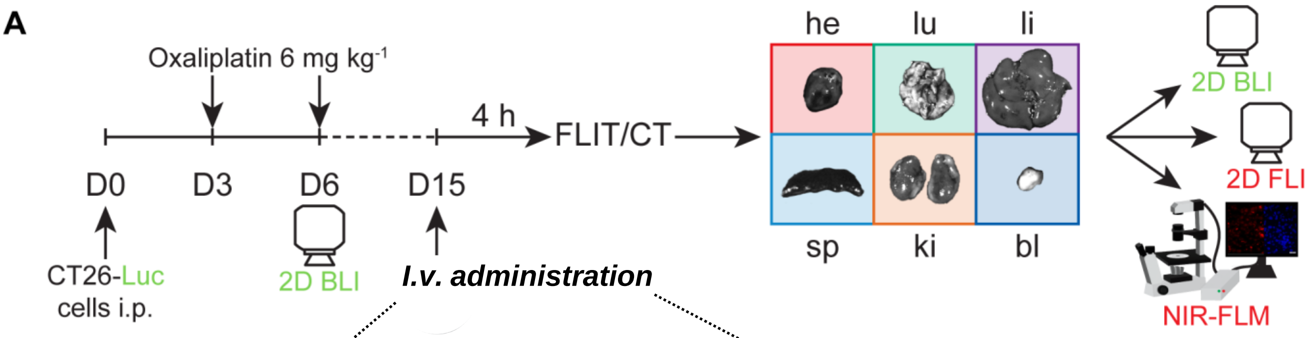

B ***Treatment groups for intravenous administration based biodistribution studies***

CT26-tumor bearing and untreated (UT)	Mouse M1
CT26-tumor bearing and treated with naked SSO-NC-750 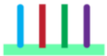	Mice M2, M3 and M4
CT26-tumor bearing and treated with cLPEI-SSO-NC-750 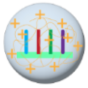	Mice M5, M6 and M7

Figure S18. Biodistribution of SSO in colorectal metastasis tumor bearing mice after systemic administration of cLPEI-SSO polyplex nanocarrier or naked SSOs. (**A**) Schematic showing tumor implantation and treatment schedule including CT26-Luc tumor imaging by firefly luciferase bioluminescence imaging (2D BLI). On day15, CT26-Luc tumor bearing mice received i.v. injection of different treatments and SSO biodistribution followed non-invasively after 4 hours using fluorescence imaging tomography/X-ray absorption computed tomography (FLIT/CT). Ex vivo organ imaging was performed to visualize tumor nodules via bioluminescence imaging (2D BLI) and SSO presence by fluorescence imaging (2D FLI) for organ-level investigation. Selected organs were then subjected to near-infrared fluorescence microscopy analysis (NIR-FLM) for visualizing nuclear delivery of SSO. (B) Different treatment groups for D15 injection and their corresponding mice numbers. Results from these treatments are presented in figure 10, S19 (FLIT/CT readout), S20 (2D BLI and 2D FLI readout) and S22-S31 (NIR-FLM based histological readout).

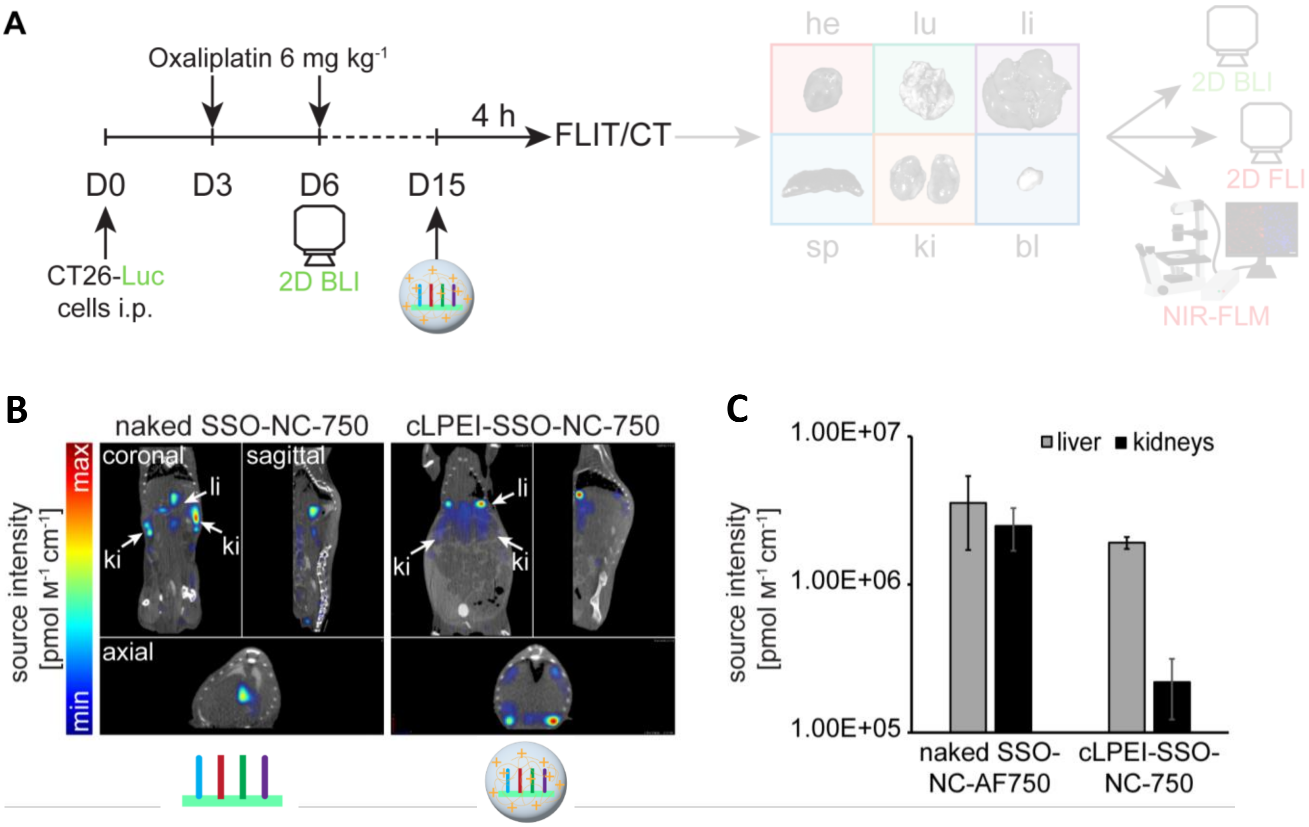

Figure S19. FLIT/CT based biodistribution of SSO in colorectal metastasis tumor bearing mice after systemic administration of cLPEI-SSO polyplex nanocarrier or naked SSOs.

Mice were treated as shown in schematic (A) and as described in figure S17-S18. On day15, CT26-Luc tumor bearing mice received i.v. injection of N/P 9 cLPEI-SSO polyplex or naked SSOs based on AF750-labelled SSOs. AF750-SSO biodistribution was then followed non-invasively after 4 hours using (B) fluorescence imaging tomography/X-ray absorption computed tomography (FLIT/CT) and (C) quantification of AF750 signal from FLIT/CT.

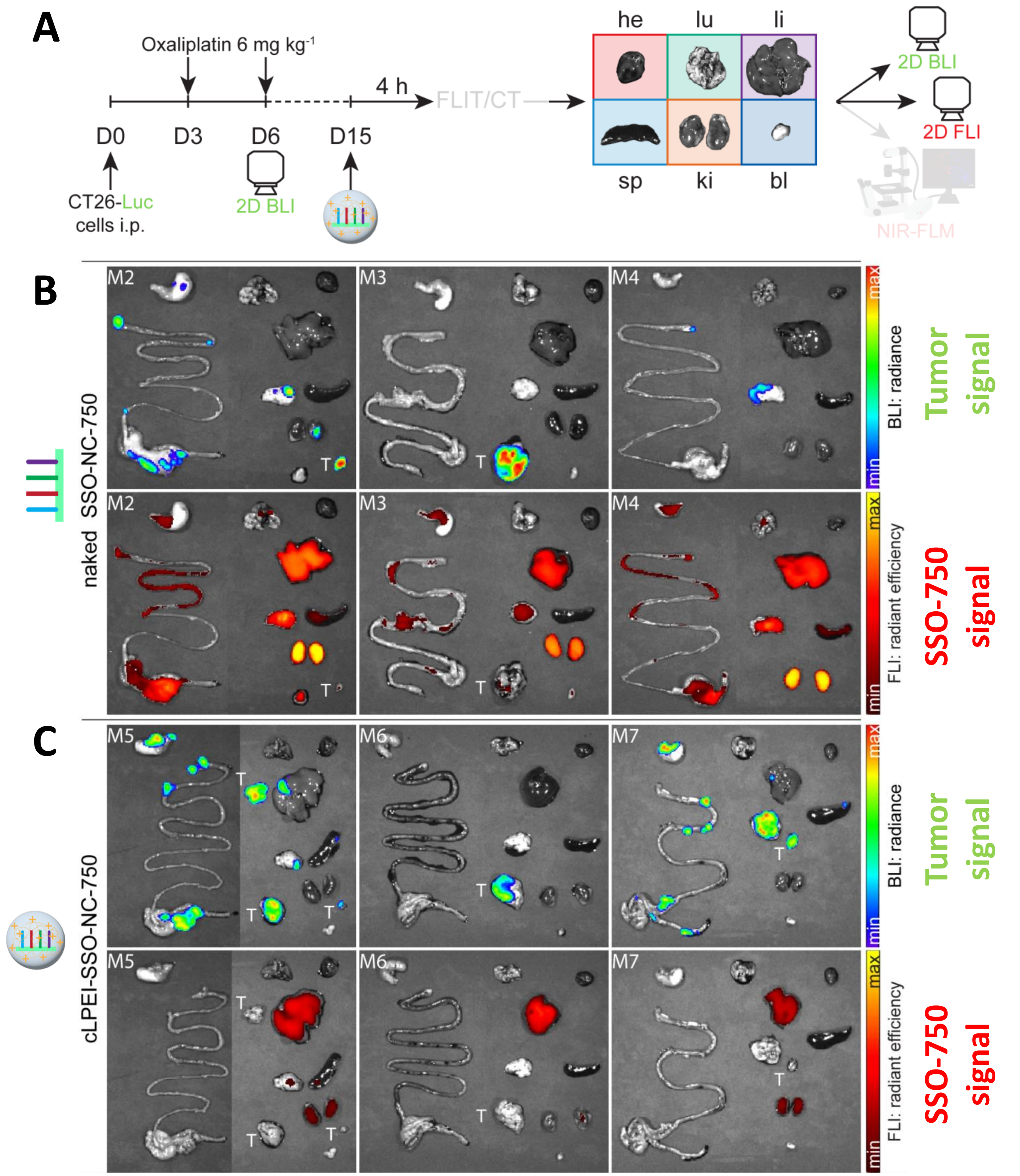

Figure S20. 2D FLI based biodistribution of SSO in colorectal metastasis tumor bearing mice after systemic administration of cLPEI-SSO polyplex nanocarrier or naked SSOs. Mice were treated as shown in schematic (A) and as described in figure S17-S18. On day15, CT26-Luc tumor bearing mice received i.v. injection of (B) naked SSOs or (C) N/P 9 cLPEI-SSO polyplex based on AF750-labelled SSOs. Post FLIT/CT imaging, first tumor nodule presence in organs was visualized by 2D BLI ex vivo organ imaging (tumor signal), which was followed by AF750-SSO biodistribution in organs by 2D FLI ex vivo organ imaging (AF750 SSO signal) of same set of organs. T: tumor; tumors are placed next to the organ they were attached to. M2-M4: naked SSO-NC-750; M5-M7: cLPEI-SSO-NC-750. Values were measured in radiance ($ps^{-1}cm^{-2}sr^{-1}$) for BLI; threshold used for images: min: 2.06e5, max: 1.43e7. Unit for FLI is radiant efficiency ($ps^{-1}cm^{-2}sr^{-1}(\mu Wcm^{-2})^{-1}$), threshold used for images: min: 1.00e8, max: 1.22e9. Mx: mouse number. M1: untreated control mouse is show in figure S21.

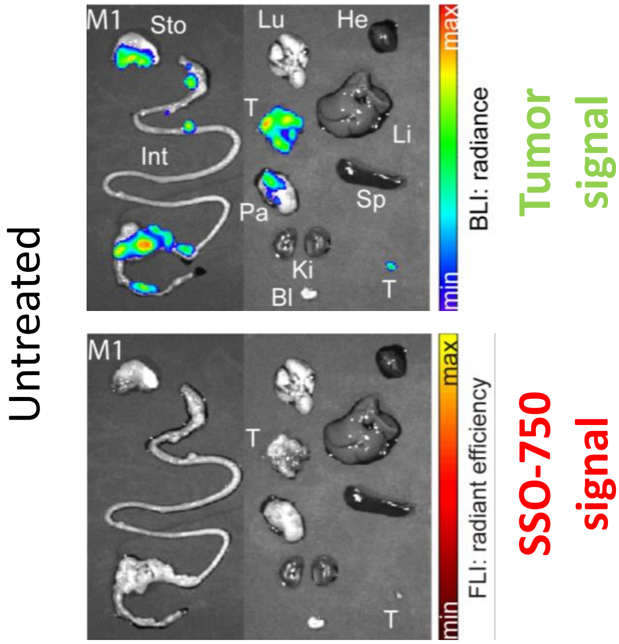

Figure S21. Colorectal metastasis tumor bearing mouse M1, which was employed as an untreated control mouse i.e. not treated with any AF750-SSO formulation, as explained in figure S18.

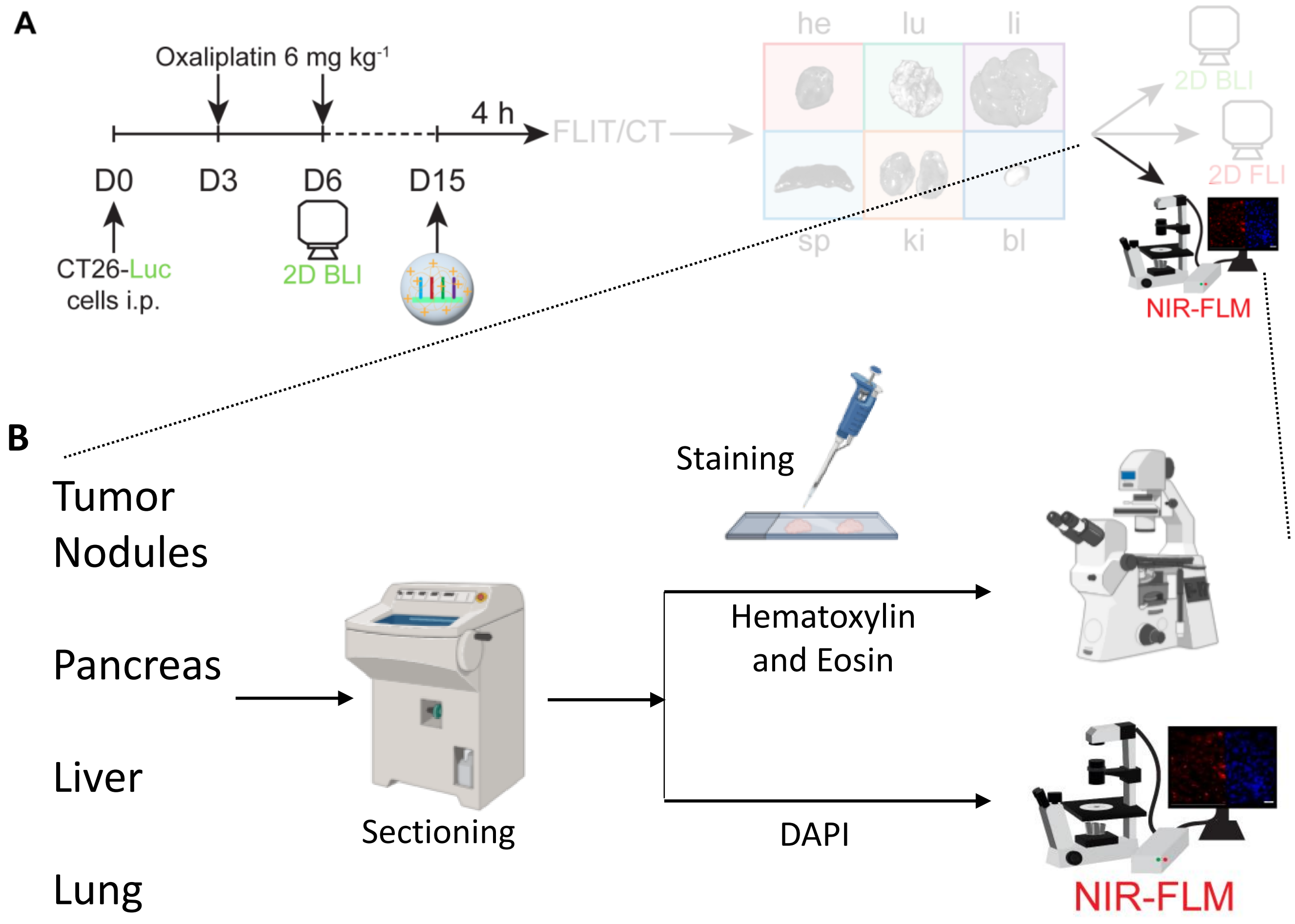

Figure S22. Near-infrared fluorescence microscopy analysis (**NIR-FLM)** based biodistribution of SSO in colorectal metastasis tumor bearing mice after systemic administration of cLPEI-SSO polyplex nanocarrier or naked SSOs.

Mice were treated as shown in schematic (A) and as described in figure S17-S18. On day15, CT26-Luc tumor bearing mice received i.v. injection of naked SSOs or N/P 9 cLPEI-SSO polyplex based on AF750-labelled SSOs. Post FLIT/CT and 2D FLI/BLI imaging, selected organs (B) were then subjected to cryo-sectioning, followed by near-infrared fluorescence microscopy analysis (NIR-FLM, for visualizing nuclear delivery of SSO) and histological analysis. Results from NIR-FLM and histological analysis are presented in figures from S23 to S31.

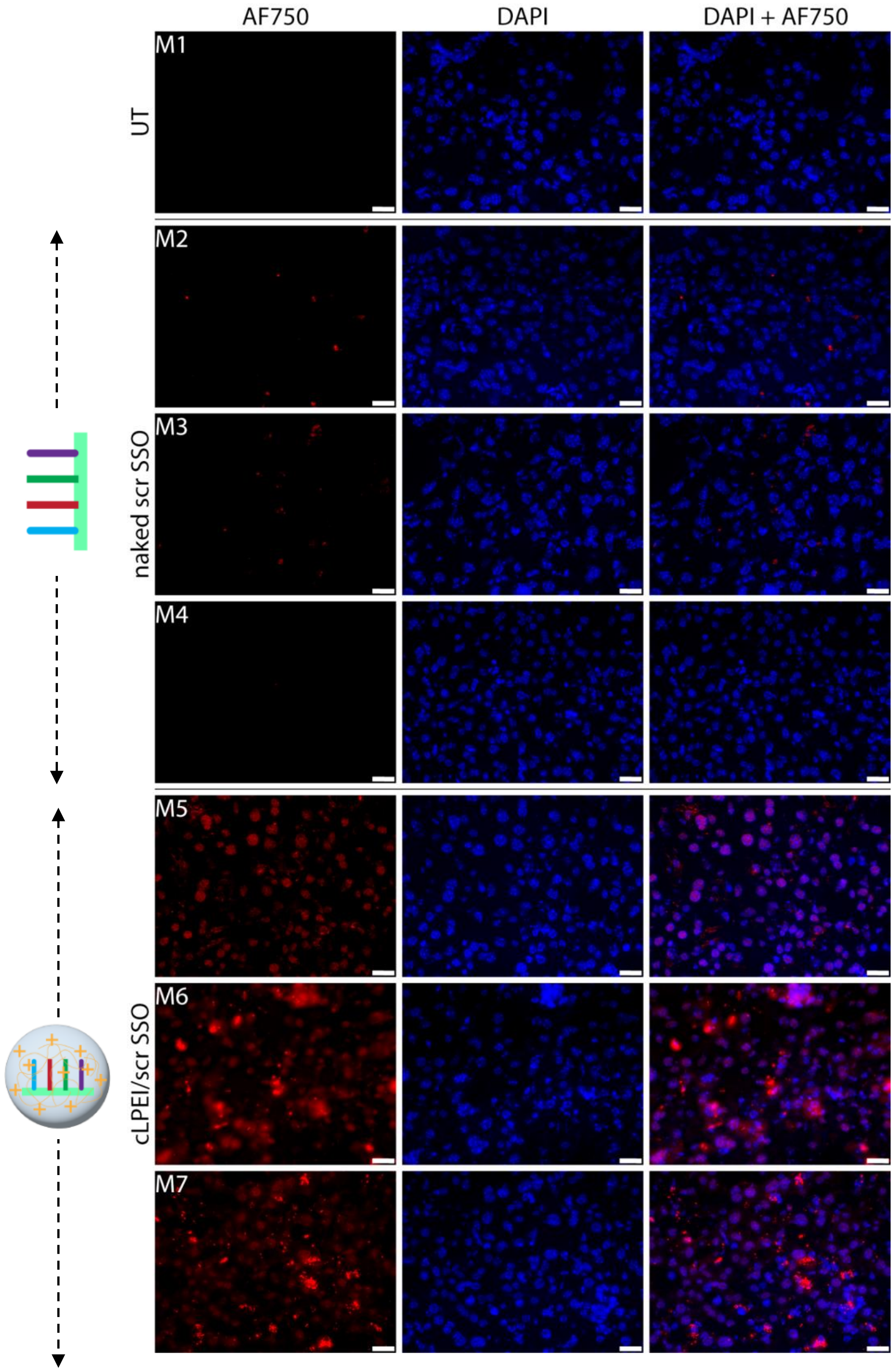

Figure S23. SSO accumulation and nuclear entry in **liver** by NIR-FLM. Mice were treated as described in figure S17-S18 and organs processed as described in figure S22. After euthanasia livers were explanted, cryosectioned and nuclei counterstained with DAPI (blue). Microscopy was performed on an Olympus IX73 inverted microscope equipped for fluorescence imaging with a 63xOil objective. Z-stacks (31 layers) with a z-spacing of 0.3 µm were acquired. Deconvolution and maximum projection were performed using cellSens Dimensions. M1: UT, M2-M4: naked SSO-NC-750; M5-M7: cLPEI-SSO-NC-750. One field of view is shown per animal: left column shows SSO-NC-750 (red), middle column DAPI and right column an overlay. Scale bar: 20 µm.

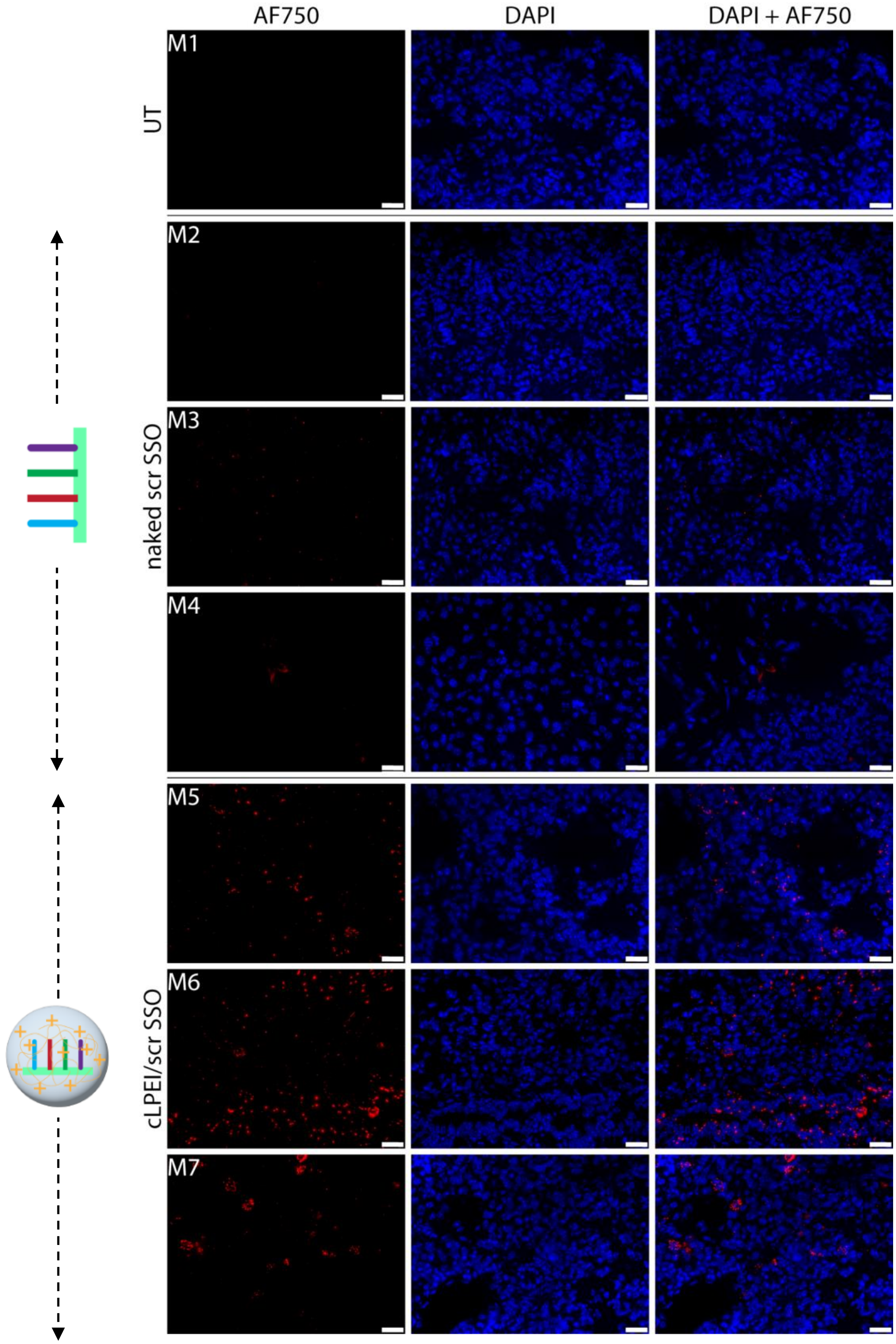

Figure S24. SSO accumulation in **lung** by NIR-FLM. Mice were treated as described in figure S17-S18 and organs processed as described in figure S22. After euthanasia lungs were explanted, cryosectioned and nuclei counterstained with DAPI (blue). Microscopy was performed on an Olympus IX73 inverted microscope equipped for fluorescence imaging with a 63xOil objective. Z-stacks (31 layers) with a z-spacing of 0.3 µm were acquired. Deconvolution and maximum projection were performed using cellSens Dimensions. M1: UT, M2-M4: naked SSO-NC-750; M5-M7: cLPEI-SSO-NC-750. One field of view is shown per animal: left column shows SSO-NC-750 (red), middle column DAPI and right column an overlay. Scale bar: 20 µm.

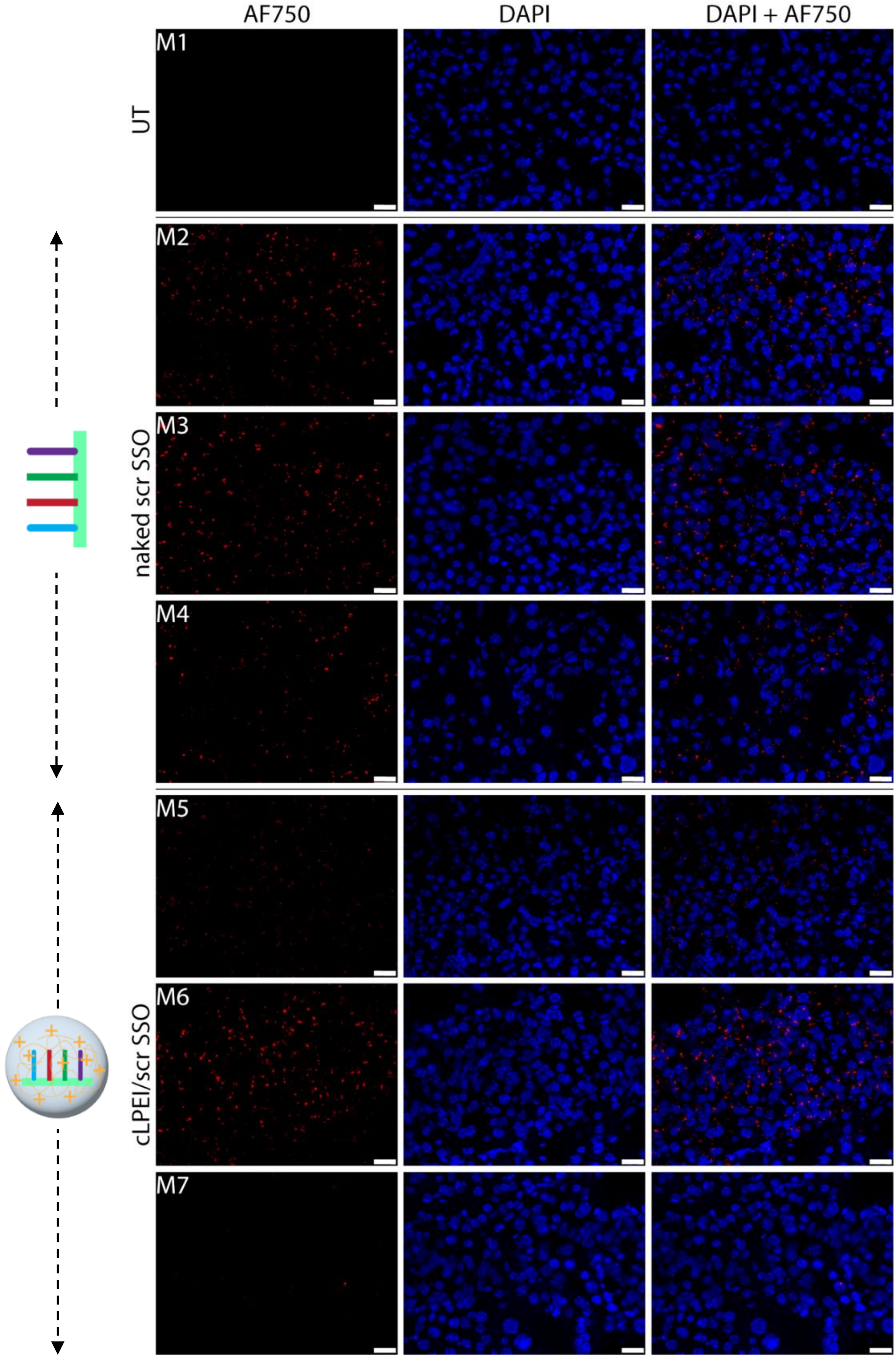

Figure S25. SSO accumulation in **non-tumorigenic areas of the pancreas** by NIR-FLM. Mice were treated as described in figure S17-S18 and organs processed as described in figure S22. After euthanasia pancreases and adjacent tumors were explanted, cryosectioned and nuclei counterstained with DAPI (blue). Microscopy was performed on an Olympus IX73 inverted microscope equipped for fluorescence imaging with a 63xOil objective. Z-stacks (31 layers) with a z-spacing of 0.3 µm were acquired. Deconvolution and maximum projection were performed using cellSens Dimensions. M1: UT, M2-M4: naked SSO-NC-750; M5-M7: cLPEI-SSO-NC-750. One field of view is shown per animal: left column shows SSO-NC-750 (red), middle column DAPI and right column an overlay. Scale bar: 20 µm.

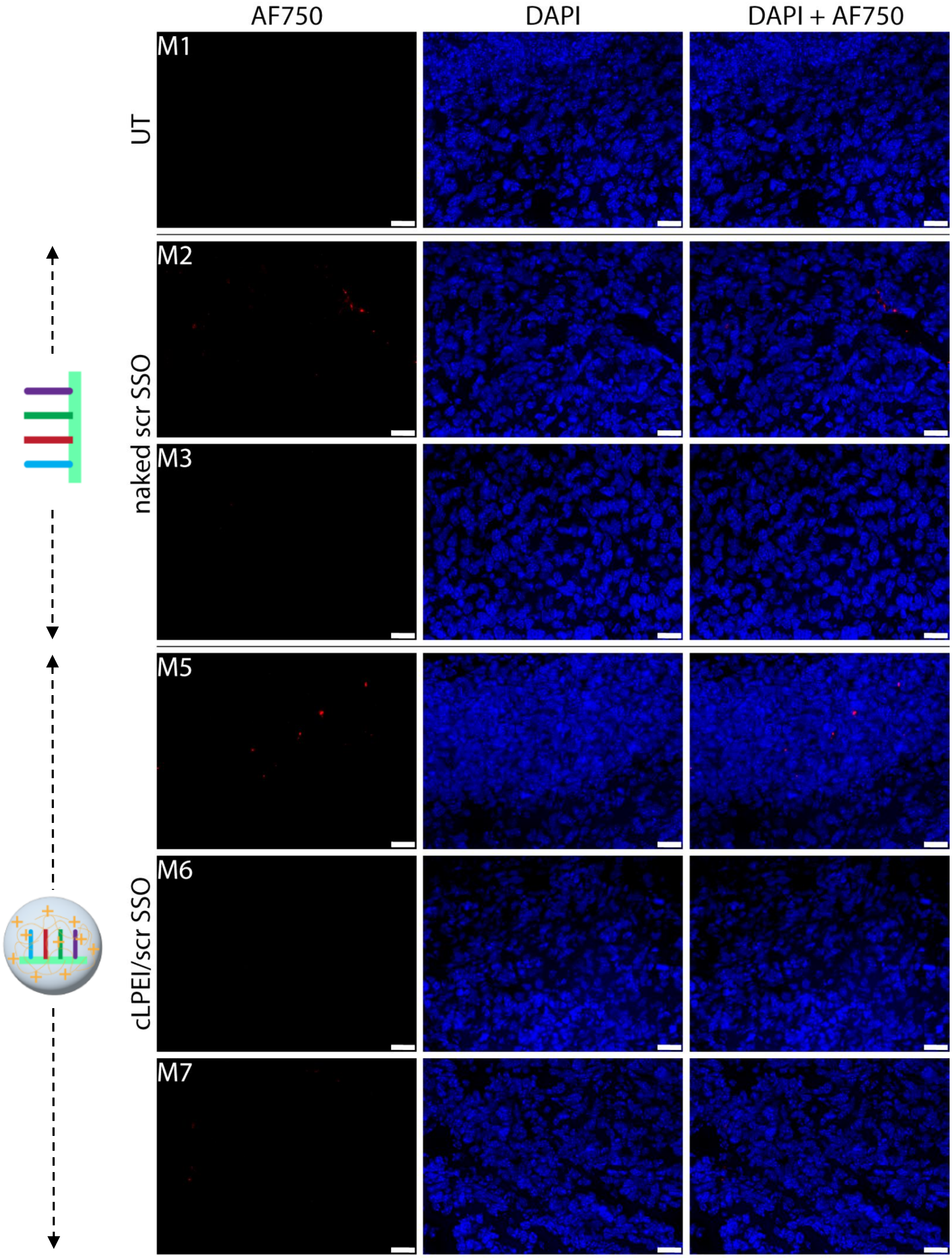

Figure S26. SSO accumulation in **individual tumor nodules** by NIR-FLM. Mice were treated as described in figure S17-S18 and organs processed as described in figure S22. After euthanasia tumors were explanted, cryosectioned and nuclei counterstained with DAPI (blue). Microscopy was performed on an Olympus IX73 inverted microscope equipped for fluorescence imaging with a 63xOil objective. Z-stacks (31 layers) with a z-spacing of 0.3 µm were acquired. Deconvolution and maximum projection were performed using cellSens Dimensions. M1: UT, M2-M4: naked SSO-NC-750; M5-M7: cLPEI-SSO-NC-750. One field of view is shown per animal: left column shows SSO-NC-750 (red), middle column DAPI and right column an overlay. Scale bar: 20 µm.

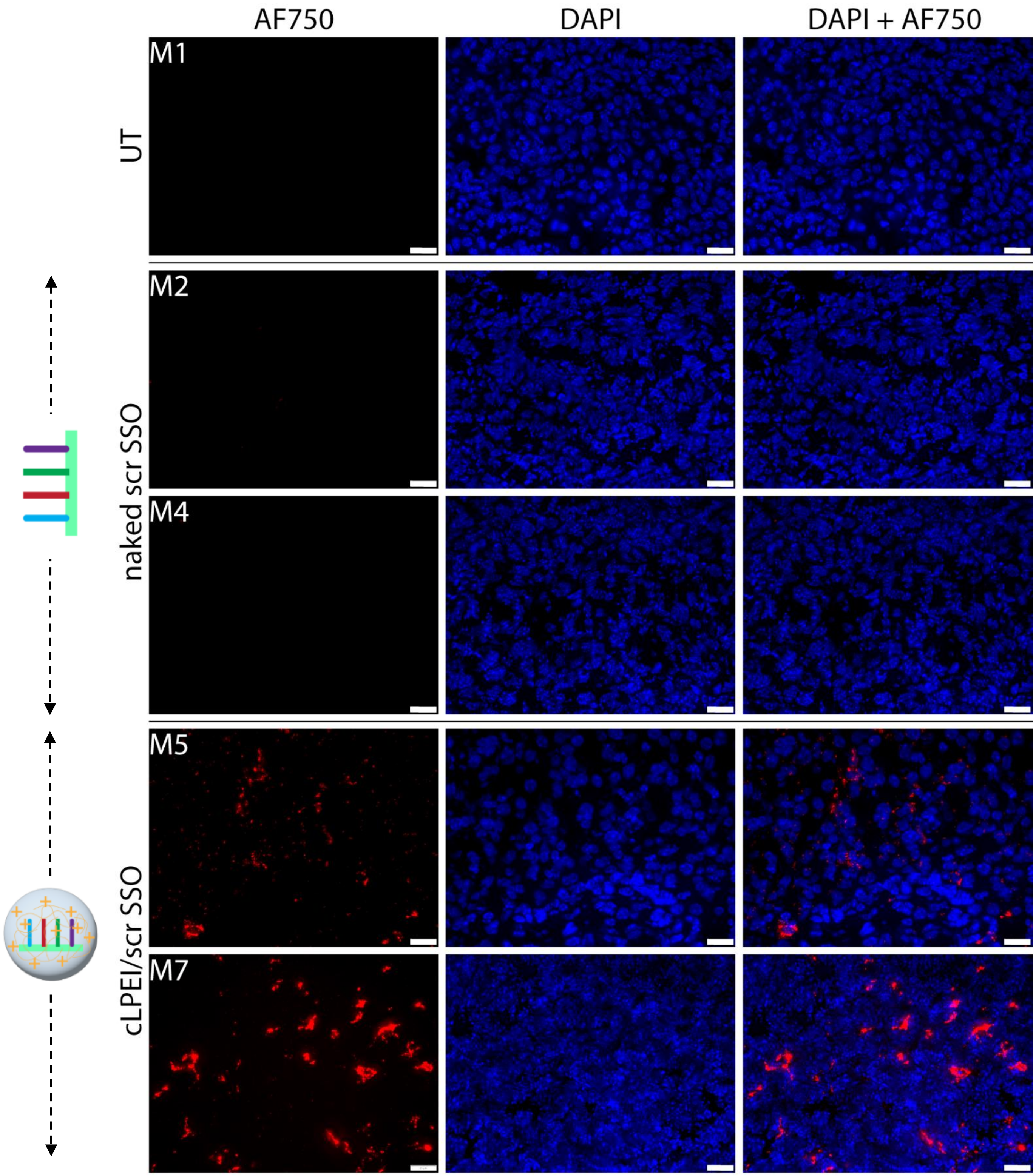

Figure S27. SSO accumulation in **pancreatic tumor nodules** by NIR-FLM. Mice were treated as described in figure S17-S18 and organs processed as described in figure S22. After euthanasia pancreases and adjacent tumors were explanted, cryosectioned and nuclei counterstained with DAPI (blue). Microscopy was performed on an Olympus IX73 inverted microscope equipped for fluorescence imaging with a 63xOil objective. Z-stacks (31 layers) with a z-spacing of 0.3 µm were acquired. Deconvolution and maximum projection were performed using cellSens Dimensions. M1: UT, M2-M4: naked SSO-NC-750; M5-M7: cLPEI-SSO-NC-750. One field of view is shown per animal: left column shows SSO-NC-750 (red), middle column DAPI and right column an overlay. Scale bar: 20 µm.

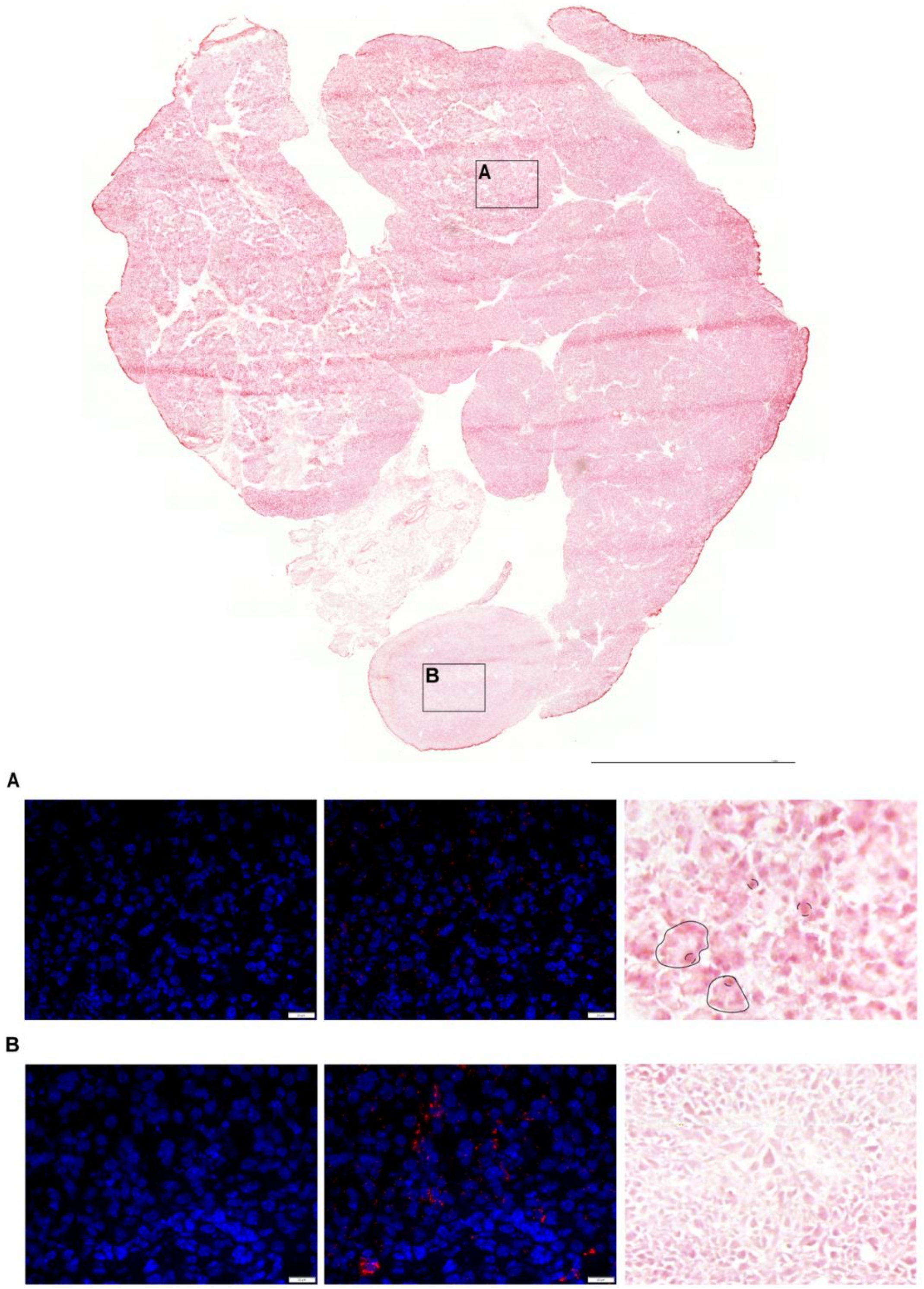

Figure S28. SSO accumulation in **pancreatic tumor nodules** by NIR-FLM and histological analysis. Mice were treated as described in figure S17-S18 and organs processed as described in figure S22. Sections were stained with DAPI for NIR-FLM and hematoxylin and eosin for microscopy. Area in pancreas (A) without tumor nodules and (B) with tumor nodules. SSO-NC-750 signal- red and DAPI signal- blue.

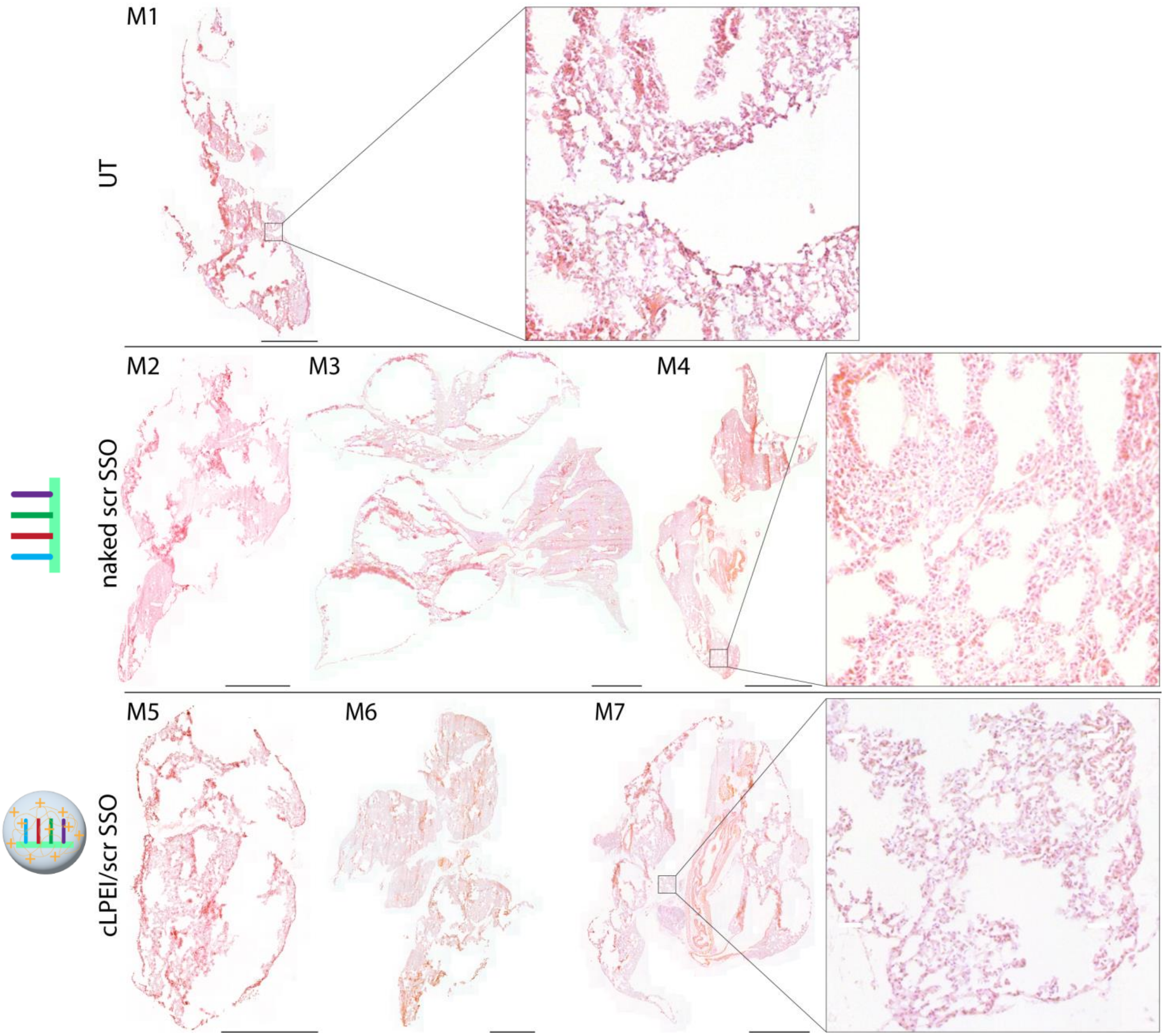

Figure S29. Lung morphology. Mice were treated as described in figure S17-S18 and organs processed as described in figure S22. After euthanasia lungs were explanted, cryosectioned and H&E stained. Whole specimens were scanned on an Olympus BX53 light microscope (Olympus, Japan) equipped with an Olympus DP-73 color camera. Magnifications for individual mice show the liver morphology in more detail. M1: UT, M2-M4: naked SSO-NC-750; M5-M7: cLPEI-SSO-NC-750. Scale bar: 2000 µm

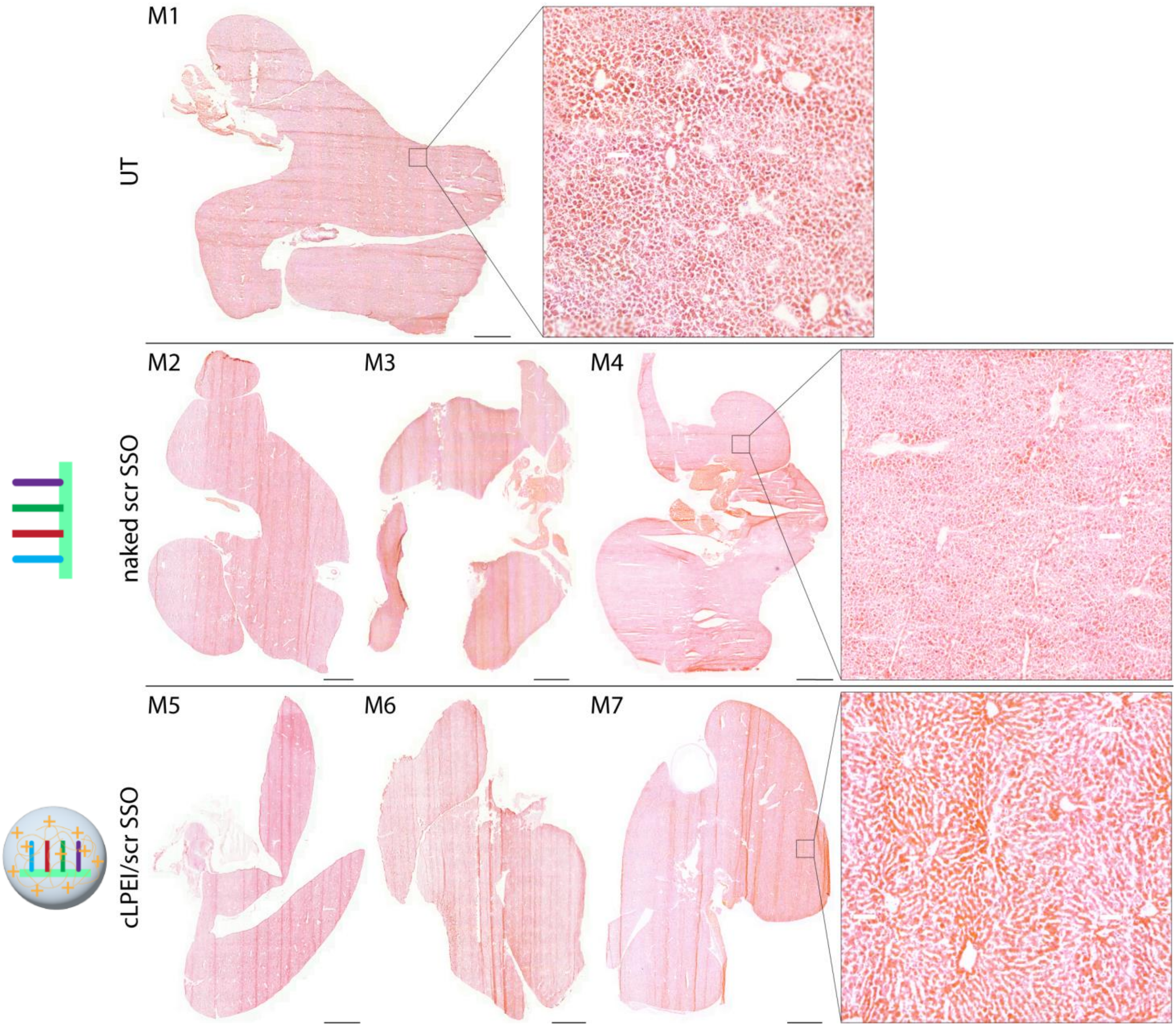

Figure S30. Liver morphology. Mice were treated as described in figure S17-S18 and organs processed as described in figure S22. After euthanasia livers were explanted, cryosectioned and H&E stained. Whole specimens were scanned on an Olympus BX53 light microscope (Olympus, Japan) equipped with an Olympus DP-73 color camera. Magnifications for individual mice show the liver morphology in more detail. M1: UT, M2-M4: naked SSO-NC-750; M5-M7: cLPEI-SSO-NC-750. Scale bar: 2000 µm

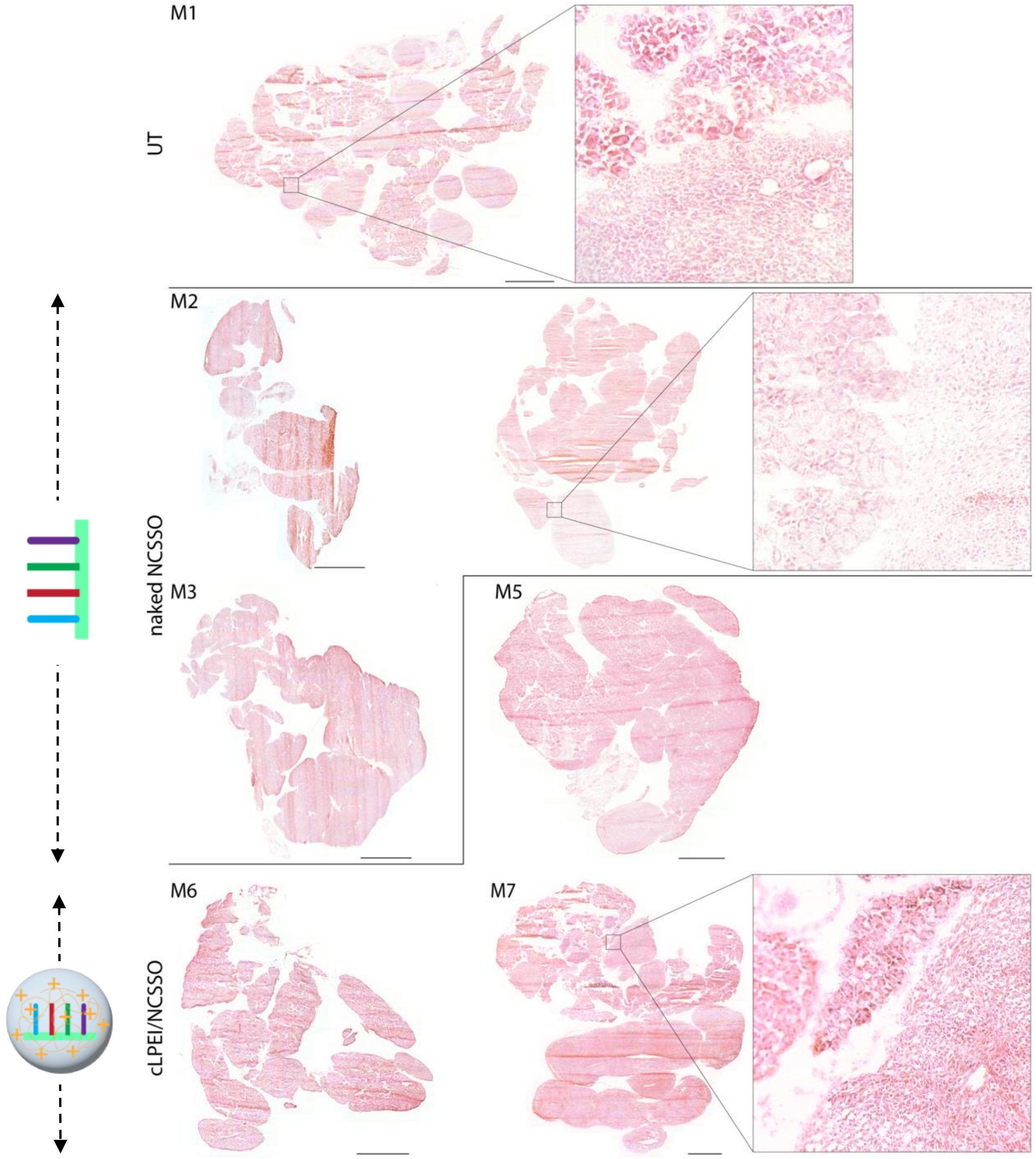

Figure S31. Pancreas morphology. Mice were treated as described in figure S17-S18 and organs processed as described in figure S22. After euthanasia pancreases were explanted, cryosectioned and H&E stained. Whole specimens were scanned on an Olympus BX53 light microscope (Olympus, Japan) equipped with an Olympus DP-73 color camera. Magnifications for individual mice show the liver morphology in more detail. M1: UT, M2-M4: naked SSO-NC-750; M5-M7: cLPEI-SSO-NC-750. Scale bar: 2000 µm
